# Patient Empowerment Improved Perioperative Quality of Care in Cancer Patients Aged ≥ 65 Years – A Randomized Controlled Trial

**DOI:** 10.1371/journal.pone.0137824

**Published:** 2015-09-17

**Authors:** Maren Schmidt, Rahel Eckardt, Kathrin Scholtz, Bruno Neuner, Vera von Dossow-Hanfstingl, Jalid Sehouli, Christian G. Stief, Klaus-Dieter Wernecke, Claudia D. Spies, PERATECS Group

**Affiliations:** 1 Department of Anesthesiology and Intensive Care Medicine, Campus Charité Mitte and Campus Virchow-Klinikum, Charité-Universitaetsmedizin Berlin, Berlin, Germany; 2 Charité Research Group on Geriatrics, Charité- Universitaetsmedizin Berlin, Berlin, Germany; 3 Department of Anesthesiology, University Hospital Munich-Grosshadern, Ludwig-Maximilians-University Munich, Munich, Germany; 4 Department of Gynecology, European Competence Center of Ovarian Cancer, Charité Campus Virchow-Klinikum, Charité- Universitaetsmedizin Berlin, Berlin, Germany; 5 Department of Urology, University Hospital Munich-Grosshadern, Ludwig-Maximilians-University Munich, Munich, Germany; 6 Charité-Universitätsmedizin Berlin and SoStAna GmbH, Berlin, Germany; University Hospital Oldenburg, GERMANY

## Abstract

**Purpose:**

This randomized controlled, clinical prospective interventional trial was aimed at exploring the effect of patient empowerment on short- and long-term outcomes after major oncologic surgery in elderly cancer patients.

**Methods:**

This trial was performed from February 2011 to January 2014 at two tertiary medical centers in Germany. The study included patients aged 65 years and older undergoing elective surgery for gastro-intestinal, genitourinary, and thoracic cancer. The patients were randomly assigned to the intervention group, i.e. patient empowerment through information booklet and diary keeping, or to the control group, which received standard care. Randomization was done by block randomization in blocks of four in order of enrollment. The primary outcome were 1,postoperative length of hospital stay (LOS) and 2. long-term global health-related quality of life (HRQoL) one year postoperatively. HRQoL was assessed using the EORTC QLQ C30 questionnaire. Secondary outcomes encompassed postoperative stress and complications. Further objectives were the identification of predictors of LOS, and HRQoL at 12 months.

**Results:**

Overall 652 patients were included. The mean age was 72 ± 4.9 years, and the majority of patients were male (68.6%, n = 447). The ^median of postoperative length of stay was 9 days (IQR 7–14 day). There were no significant differences between the intervention and the control groups in postoperative LOS (p = 0.99) or global HRQoL after one year (women: p = 0.54, men: p = 0.94). While overall complications and major complications occurred in 74% and 24% of the cases, respectively, frequency and severity of complications did not differ significantly between the groups. Patients in the intervention group reported significantly less postoperative pain (p = 0.03) than the control group. Independent predictors for LOS were identified as severity of surgery, length of anesthesia, major postoperative complications, nutritional state, and pre-operative physical functional capacity measured by the Timed Up and Go-test by multiple robust regressions.

**Conclusion:**

Patient empowerment through information booklet and diary keeping did not shorten the postoperative LOS in elderly onco-surgical patients, but improved quality of care regarding postoperative pain. Postoperative length of stay is influenced by pre-operative nutritional state, pre-operative functional impairment, severity of surgery, and length of anesthesia.

**Trial Registration:**

Clinicaltrials.gov. Identifier NCT01278537

## Introduction

Cancer is among the leading cause of morbidity and death worldwide]. The incidence of (solid) cancer increases substantially with age [1]. With the increase of life expectancy in the developed countries, the incidence of cancer, and respectively volume of cancer surgery in patients older than 65 years, increases steadily. In Europe, it has been estimated that this age group accounts for approximately 58% of all cancers, and 69% of cancer deaths [1].

Surgery, as the “state of the art” therapy for most solid tumors, must now be performed in elderly patients, a major risk group with the highest perioperative mortality rate, ranging from 7% to 15% [2,3], prone to prolonged hospital stay [4,5]. Postoperative morbidity and mortality is associated with an immense socio-economic burden, making prevention of postoperative complications, improved quality of care, and reduced length of in-hospital stay a permanent interest of research [6].

As early as in 1958, Janis described that successful emotional inoculation could be achieved in patients facing severe stress by giving them preparatory information containing accurate warnings about what to expect [7]. Since then many studies investigated the influence of psycho-educational interventions on postoperative recovery [8–10]. Frequently analyzed outcomes were anxiety, pain and health-related quality of life [8,10].

In summary, providing pre-operative education as a form of patient empowerment, including the provision of preparatory information about the postoperative limitations, could improve the outcome, although evidence for this is still inconclusive [6,10–12]. Further, the studies hitherto published were conducted only for minor surgical procedures or cardiac bypass surgery comprising only small patient populations [6]. To our knowledge, the present trial is the first such study focusing on major onco-surgery in elderly patients.

This RCT was aimed at investigating whether the short- and long-term outcome of elderly onco-surgical patients can be improved by patient empowerment, with specifically designed information, compared to a standard-of-care control group. Primary clinical outcomes were in-hospital length of stay (short-term perioperative risk) and global health-related quality of life (HRQoL, long-term perioperative risk). Secondary outcomes included perioperative quality of care such as postoperative stress, e.g. level of pain and postoperative complications.

## Materials and Methods

### Ethics Statement

The study PERATECS (“Patient empowerment and risk-assessed treatment to improve outcome in the elderly after gastrointestinal, thoracic or urogenitary cancer surgery”) was conducted in compliance with the Helsinki declaration. The Institutional Review Board of Charité-Universitaetsmedizin Berlin approved the study (EA 2/241/08). The study is registered at ClinicalTrials.gov (NCT01278537). All participants provided written informed consent.

### Study design

The study was performed as a randomized, controlled, open-label, clinical prospective double-center interventional study, at two tertiary medical care university hospitals in Germany (Charité, Universitätsmedizin Berlin (3200 Beds), Klinikum der Universität München (KUM) (2244 beds)).

### Data collection and inclusion and exclusion criteria

From February 2011 to September 2012, all patients older than 65 years scheduled for surgery for gastro-intestinal, genitourinary, gynecological or thoracic cancer were screened for eligibility. Follow-ups were conducted 3 and 12 months after surgery. Last patient last visit was completed in January 2014.

Inclusion criteria were age ≥ 65 years, major onco-surgery, proficiency in the German language, a Mini Mental Score (MMSE) of 24 points or higher, as well as written informed consent.

**Fig 1:**
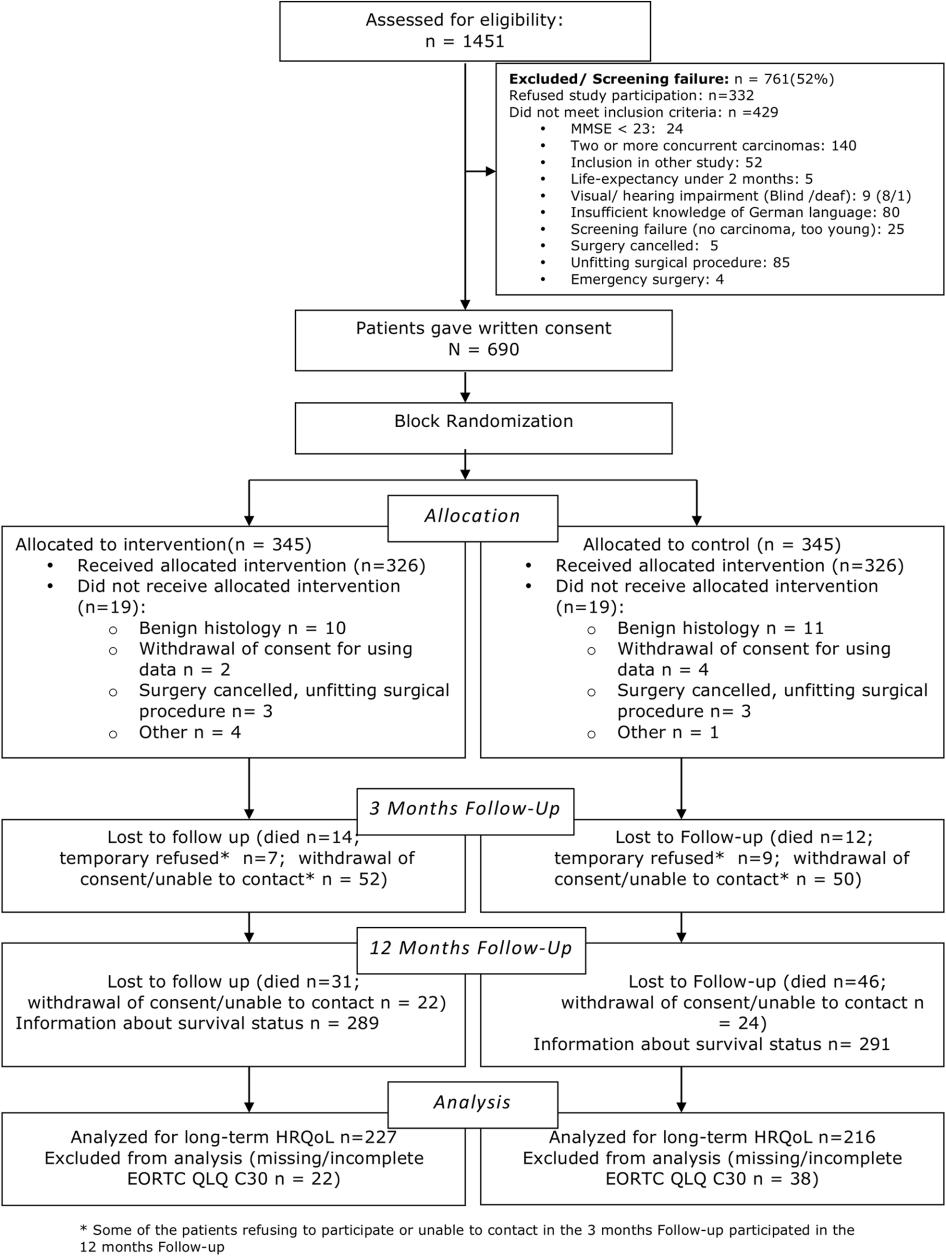
Study flow diagram.

Exclusion criteria included, two or more concurrent carcinomas, emergency surgery, life expectancy of less than 2 months, as well as current participation in another trial. Patients lacking proficiency in the German language, living in a closed institution due to an official or judicial order, as well as patients unable or unwilling to provide informed consent could not participate (Fig 1).

### Study Groups

After screening and enrollment in the study, patients were randomly allocated to either the intervention group or the control group using block randomization with a block size of four.

All participants in the trial received standard care, consisting of two separate visits from the surgeon and anesthetist one day before surgery. During these visits, the surgeon and anesthetist would respond to specific concerns of the patient or their family and obtain informed consent for the proposed surgery and general anesthesia.Several parameters were recorded daily for the first five postoperative days. Patients in the intervention group received additional information regarding the perioperative period.

### Intervention

#### Information booklet and patient diary

The patients allocated to the intervention group, i.e. patient empowerment, received a booklet with additional information and a diary at least one day before surgery.

The informational booklet and the diary were designed and reviewed by psychologists, anesthesiologists, geriatricians, and nurses, and consisted of 32 illustrated pages of large print (font size 14). In a pilot study, patients were asked to evaluate the information package and the diary, with their comments considered for the final version [13].

The booklet contained information about surgery, anesthesia and perioperative management, such as fasting time and premedication. Information was also included about acute postoperative pain therapy, mobilization, and nutrition, stressing the importance of being actively involved in the rehabilitation process. Life-style-related risks following discharge from the hospital, such as lack of physical activity, unhealthy nutrition and substance abuse were described. Furthermore, the booklet contained concrete information regarding, support groups and home care institutions offering specific assistance after discharge. Patients were repetitively encouraged to play an active role in their rehabilitation process and to ask questions regarding medication and therapy.

The diary was kept daily for seven days, starting the day before the operation and continuing until five days after. The patients were encouraged to become more aware of their situation, including pain, mobilization, nutrition, etc. They were encouraged to ask for analgesics if their pain was not properly addressed, and to become more active on their own, to get out of the bed as far as possible, and were motivated to ask questions regarding their recovery and discharge.

### Control Group

Patients in the control group were only provided with the standard information regarding surgical and anesthesiological risks and procedures.

### Health-Related Quality of Life (HRQoL)

Quality of life as assessed via HRQoL scores from the European Organisation for Research and Treatment of Cancer 30-Item Core Quality of Life Questionnaire, version 3.0 (EORTC QLQ-C30), a validated questionnaire [14]. Patients completed questionnaires prior to surgery, as well as 3 and 12 months after surgery, using questionnaires sent by mail.

The EORTC QLQ-C30 questionnaire is a 30–item questionnaire incorporating nine multiple-item scales and six single items. Global health-related quality of life is a two-item score using a 7-point Likert scale. The score was linearly transformed to a score from 0 to 100 and missing items were handled as in the manual described [15]. Higher scores in global HRQoL imply a higher health-related quality of life. A difference of 5–9 point in the scores represents a small change, 10–20 points a moderate change, and more than 20 points a large change in HRQoL [16]. The EORTC QLQ-C30 is a reliable and valid instrument for assessing HRQoL in cancer patients [14,17].

In 2013, Waldmann et al.[18] published reference data for health related quality of life assessed by EORTC QLQ C-30 for the German population.

### Pre-operative Data Collection

Demographic and clinical data were recorded at baseline. The collected data included age, gender, marital, socio-demographical status, and comorbidities. Comorbidities were evaluated with the Charlson Comorbidity Score (CCS) [19]. Furthermore, the following data were documented: Eastern Cooperative Oncology Group (ECOG) performance status [20], pre-operative risk assessment according to the classification of the American Society of Anesthesiologists (ASA) [21], severity of surgery measured by the Physiological and Operative Severity Scoring system for enUmeration of Mortality and morbidity (POSSUM) [22], cancer site and presence of metastases.

The registered intra-operative parameters included duration of surgery, type of anesthesia and length of anesthesia.

Additional screening scores for pre-operative risk assessment were elements of the Pre-operative Assessment of Cancer in the Elderly (PACE) [23]. The PACE was developed from the Comprehensive Geriatric Assessment (CGA), integrating a battery of validated instruments for the geriatric assessment, i.e. the Activity of Daily Living (ADL) [24], Instrumental Activities of Daily Living (IADL) [25], the Geriatric Depression Scale (GDS) [26], the Brief Fatigue Inventory (BFI) [27], the ECOG performance status [20], and the Mini Nutritional Assessment (MNA) [28].

Cognitive assessment included the Folstein Mini-Mental State Examination (MMSE) [29].

These measurements were amended by tests assessing the physical function of the patients: the Timed Up and Go test (TUG) [30], hand grip strength, and the Tinetti Balance and Gait test, which determines the fall risk [31].

The staff performing the interviews included study doctors, medical students and study nurses. A senior physician for geriatrics trained the staff in geriatric assessment before the start of the study, with regular refresher courses over the study period. Similarly, a psychologist trained the staff in assessment of postoperative cognitive dysfunction, including completion of the specific questionnaires. The time spent for the interviews ranged from 60 to 120 minutes.

### Perioperative Treatment

Anesthesia, surgery, perioperative treatment, as well as cancer treatment, were carried out according to the standard operating procedures in the respective university hospitals [32].

### Postoperative Data Collection

Patients in both groups received daily study-related visits up to the fifth postoperative day.

Complications were assessed daily, including operational revision and intensive care transfers. Perioperative complications were defined as any event occurring during hospital stay requiring treatment measures that are not routinely applied following the specific surgery. The recorded complications were revised by two independent reviewers in accordance with the Clavien scale [33], in which a grade of III to V were defined as major complications [34].

Postoperative stress was defined as occurrence of postoperative pain, and postoperative nausea and vomiting (PONV). Postoperative pain was assessed using the numeric rating scale (NRS) [35]. Pain therapy was documented daily, as well as occurrence of PONV, time of first enteral nutrition, intestinal paralysis, and start and duration of mobilization. Furthermore, delirium screening was conducted daily using the Confusion Assessment Method for Intensive Care Units (CAM-ICU) [36] for patients on ICU or the Nursing Delirium Scale (NUDESC) [37,38] for delirium assessment in all other patients.

Also recorded were the duration of hospitalization (LOS), defined as time in hospital after surgery, as well as morbidity and in-hospital mortality.

At discharge, length of hospital stay, length of ICU stay, and discharge mode were recorded. The Case Report Forms (CRF) for complications was also completed, as well as the HRQoL, GDS, handgrip strength, and TUG. The completeness of the diaries was also documented. At discharge, patients were asked if the information package and/or the diary were useful for their rehabilitation, and answers were given using a four point Likert scale.

### Follow-up

Follow-up time was 1 year. All patients were contacted per mail 3 and 12 months after surgery. Survival status 12 months after surgery was recorded for all patients. The participants received questionnaires including the EORTC QLQ C30 by mail. If patients did not answer within two weeks, they were contacted by telephone. If the patients could still not be reached, their general practitioner (GP) was contacted and asked about patients’ survival status. Further data about mortality was obtained from the clinical cancer registry of the Charité Comprehensive Cancer Center, Charité-Universitätsmedizin Berlin, Germany.

### Statistical Analysis

#### Sample size calculation

The short time primary endpoint was the time to fulfill hospital discharge criteria. Postoperative length of hospital stay (LOS) was defined as the time interval between the date of surgery and the date of discharge. We hypothesized that an assumed LOS of 12 days could be reduced to 10 days due to fewer postoperative complications in the intervention group.

The long time primary endpoint was the global health-related quality of life of elderly patients at 12 months follow-up. Criterion for improved HRQoL was an increase of at least 5 points in the global health-related quality of life scale of the EORTC QLQ C30 within twelve months in the intervention group.

The sample size calculation was conducted with respect to the long-term primary endpoint in a worst-case scenario. Bonferroni-adjusted error of the 1^st^ kind α = 0.025 two-sided, power 80%, and the following quantities [39]: Global health-related quality of life score according to EORTC QLQ C-30 [mean (SD)]: 68.82 (15.51) [before intervention], 73.12 (13.73) [after 12 months follow-up].

Assuming the nonparametric Wilcoxon (Mann-Whitney) rank-sum test, a sample size of 230 patients per group was to be included in the study.

With 12 days versus 10 days (time to fulfill discharge criteria from the hospital) and an effect size of 0.5 (according to own studies) for the first primary, a sample size of 83 patients per group would follow (α = 0.025 two-sided, power 80%, calculations using nQuery Advisor® Release 7.0, Stat. Solutions Ltd. & South Bank, Crosse’s Green, Cork, Ireland).

The global HRQoL values after 12 months of both groups were tested for significant differences using the Mann-Whitney *U*-test. Due to the high impact of gender on global HRQoL, the data were also analyzed separately for both genders. Values of baseline and 12 months global HRQoL were descriptively compared to published normative data for the German population [18].

Categorical variables are presented as numbers and percentages. Continuous variables are presented as mean and standard deviation (SD) and, when not normally distributed as median (interquartile range, IQR). For categorical variables, differences between groups were tested using Fisher’s exact test. Differences between groups in normally distributed continuous variables were evaluated using the students’ t-test respectively the Mann-Whitney *U*-test for continuous non-normally distributed variables. Odds ratios (OR) and regression coefficients with 95% confidence intervals (CI) were determined in robust and logistic regression analysis.

To identify predictors of prolonged LOS, univariate robust regressions followed by a multiple robust regression with the endpoint postoperative length of stay were performed. Multiple robust regressions were adjusted for patient age (years), gender, tumor site (genito-urinary vs. gastrointestinary), intervention vs. control, occurrence of major complications (yes vs. none), TUG (<20 sec vs. >20 sec), length of anesthesia (minutes), educational degree (<high school vs. > high school), nutritional state (normal vs. malnutrition), and severity of surgery (minor/moderate vs. major).

Beside variables of particular clinical interest, only variables associated with a univariate significant impact (*p*<0.10) on outcome were introduced in the multivariate models. After calculation with all the chosen variables (full model), most relevant characteristics were identified by backward feature selection.

Predictors for one year global HRQoL were identified by multiple linear regressions. Included variables were: intervention vs. control, gender, age (years), ASA state (I/II vs. III/IV), Charlson Comorbidity Score (points), tumor site (genito-urinary vs. gastrointestinary), nutrition state (MNA: normal/risk for malnutrition vs. malnutrition), Timed Up and Go-test (< 20 sec vs. > 21 sec); Severity of surgery (minor/moderate vs. major), pre-operative global HRQoL (points), major complications (yes vs. none), depressions (none vs. manifest), Fatigue (no/mild vs. severe), Activities of Daily Living (points), Mini Mental State (points).The tests for the primary outcome (postoperative length of hospital stay and global health-related quality of life) have been carried out in a confirmatory understanding, but all other tests for secondary endpoints are to be understood in the area of exploratory data analysis; therefore no adjustments for multiple testing have been made.

For analysis of the course of global HRQoL from pre- to postoperative, the difference from baseline HRQoL to 1 year HRQoL for each patient was calculated. Means of the difference score between intervention and control group were tested for significance using the Mann-Whitney *U*-test. The data were also analyzed separately for both genders. Survival data were estimated according to the Kaplan-Meier methods and compared univariately with log rank statistics.

Statistical significance was defined as p < 0.05. All statistical tests were two tailed. Statistical analyses were performed using SPSS software (version 22.0 SPSS, Inc., Chicago, IL, USA) and R 3.0.3.

## Results

### Patient recruitment

The outline of patient recruitment and follow-up is shown in Fig 1. The final sample consisted of 652 patients. Of these, 326 patients were assigned to the intervention-group and 326 patients were assigned to the control-group. After 12 months, questionnaires were sent to the patients. 103 patients (15.8%) had died. We received 450 questionnaires out of 549 (79%). Loss to follow-up was 15.2% (n = 99) after 12 months. Follow-up after 3 months was of no concern for the primary endpoints and is not considered in this analysis.

### Baseline characteristics

The socio-demographic and clinical characteristics of the patients are listed in Tables 1 and 2. The mean age of all patients was 72 ± 4.9 years. There were more men (68.6%) than women (31.4%). Distribution of tumor site is also shown in Table 1. There were no differences regarding age, gender and tumor distribution between both groups (p = 0.5; p = 0.24 and p = 0.74 respectively). All other demographic and clinical data were comparable in intervention and control group (Table 1).

**Table 1:** Demographic and clinical baseline characteristics of the study population.

	Intervention	No Intervention	P
	N = 326	N = 326	
Mean Age (SD)	71.6 (4.6)	72 (5.1)	0.50^€^
Male (%)	216 (66.3%)	231 (70.9%)	0.24^#^
BMI, mean (SD)	26 (4.2)	26 (3.6)	0.49^€^
*Tumor site*			
Genito-urinary	226 (69.3%)	221 (67.8%)	0.74^#^
Abdomino-thoracic	100 (30.7%)	105 (32.2%)	
Recurrence of cancer	47 (14.4%)	44 (13.5%)	0.82^#^
*Severity of surgery* ^$^			
Moderate	9 (2.8%)	9 (2.8%)	0.97^#^
Major	183 (56.1%)	186 (57.1%)	
Major+	134 (41.1%)	131 (40.2%)	
Pre-operative Chemotherapy	40 (12.3%)	34 (10.4%)	0.54^#^
Pre-operative Radiotherapy	11 (3.4%)	16 (4.9%)	0.43^#^
ASA I/II	219 (67.2%)	206 (63.2%)	0.32^#^
ASA III/IV	107 (32.8%)	120 (36.8%)	
*Performance State ECOG*			
0	315 (96.6%)	311 (95.4%)	0.65^#^
1	8 (2.5%)	12 (3.7%)	
2/3	3 (0.9%)	3 (0.9%)	
Comorbidities ^§^, Median (IQR)	2 (2; 4)	2 (2; 4)	0.42^⌘^
*Nutrition state (n = 629)* ^&^			
Normal	222 (70.9%)	230 (72.8%)	0.84^#^
Risk for malnutrition	83 (26.5%)	77 (24.4%)	
Malnourished	8 (2.6%)	9 (2.8%)	
*Mean hand grip strength [kg] (SD)*			
Women	23.3 (5.3)	23.1 (5.6)	0.83^⌘^
Men	39.2 (8.5)	38.9 (8.5)	0.92^⌘^
Living alone at home	64(20.8%)	62 (20.5%)	>0.99^#^
Living at home with public help	16 (5.4%)	11 (3.7%)	0.33^#^
*School degree (%; n = 589)*			
>High school degree	109 (37.2%)	100 (33.83%)	0.39^#^
Surgery: median length [minutes](IQR)	165 (105; 250)	170 (105; 260)	0,42^⌘^
Anesthesia: median length [minutes](IQR)	215 (140; 330)	220 (150; 330)	0,56^⌘^

SD = Standard deviation, BMI = Body mass index

^$^POSSUM: Physiological and Operative Severity Score for the enUmeration of Mortality and Morbidity, IQR = Interquartile Range, MMSE = Mini Mental State, EORTC = European Organisation of Research and Treatment of Cancer, ASA = American Society of Anesthesiologists, ECOG =: Eastern Cooperative Oncology Group

^§^ CCS = Charlson Comorbidity Score

^&^ Mini Nutritional Assessment (MNA)/BMI

^€^: Students’ t-test

^#^: χ2-Test (Fisher’s Exact test)

^⌘^: Mann Whitney U-Test

**Table 2:** Elements of the pre-operative Comprehensive Geriatric Assessment.

	Intervention	No Intervention	P
	N = 326	N = 326	
ADL, Median (IQR)	100 (100; 100)	100 (100; 100)	0.95^⌘^
ADL < 100 (%) (n = 645)	58 (18.0%)	57 (17.7%)	0.99^#^
IADL, Median (IQR)	8 (8;8)	8 (8;8)	0.81^⌘^
IADL < 8 (%)	53 (16.3%)	54 (16.6%)	0.95^#^
MMSE, median (IQR)	29 (28; 30)	29 (28; 30)	0.49^⌘^
MMSE <27 (%)	28 (8.6%)	32 (9.8%)	0.69^#^
*Fatigue (BFI) (n = 575)*			
Mild fatigue	136 (47.6%)	113 (39.1%)	0.10^#^
Moderate fatigue	53 (18.5%)	68 (23.5%)	
Severe fatigue	7 (2.4%)	14 (4.8%)	
*Depression (GDS) (n = 586)*			
No depression	256 (88.0%)	265 (89.8%)	0.51^#^
Risk for /Manifest depression	35 (12.0%)	30 (10.2%)	
*Timed up and go test (TUG) (n = 619)*			
< 10 sec	231 (74.5%)	229 (74.1%)	0.92^#^
11–20 sec	64 (20.6%)	68 (22.0%)	
21–30 sec	7 (2.3%)	6 (1.9%)	
> 31 sec	8 (2.6%)	6 (1.9%)	
TUG >10 sec	79 (25.5%)	80 (25.9%)	0.93^#^
TUG >20 sec	15 (4.8%)	12 (3.9%)	0.70^#^
*Risk of falls (Tinetti Score) (n = 617)*			
High (≤18 pts.)	9 (2.9%)	12 (3.9%)	0.56^#^
Moderate (19–23 pts.)	15 (4.8%)	20 (6.5%)	
Low (≥24 pts.)	287 (92.3%)	274 (89.5%)	

ADL = Activities of daily living, IADL = Instrumental activities of daily living, IQR = Interquartile Range, TUG = Timed up and go, BFI = Brief fatigue inventory, GDS = Geriatric Depression Scale, SD = Standard Deviation.

#: χ2-Test (Fisher’s Exact test)

⌘: Mann Whitney U-Test

Furthermore, there were no differences regarding functional and cognitive tests assessed by the geriatric assessment (Table 2).

In the intervention group, 193 (59%) patients rated the empowerment package as helpful or very helpful. A narrow majority of 186 (57%) kept the diary for at least 50% of the planned days, whereas 82 (25%) patients kept the diary for less than 50% of the planned days. In 58 cases (17.8%) information about diary keeping is entirely missing.

### Primary short-term outcome

#### Postoperative in-hospital stay

**Fig 2:**
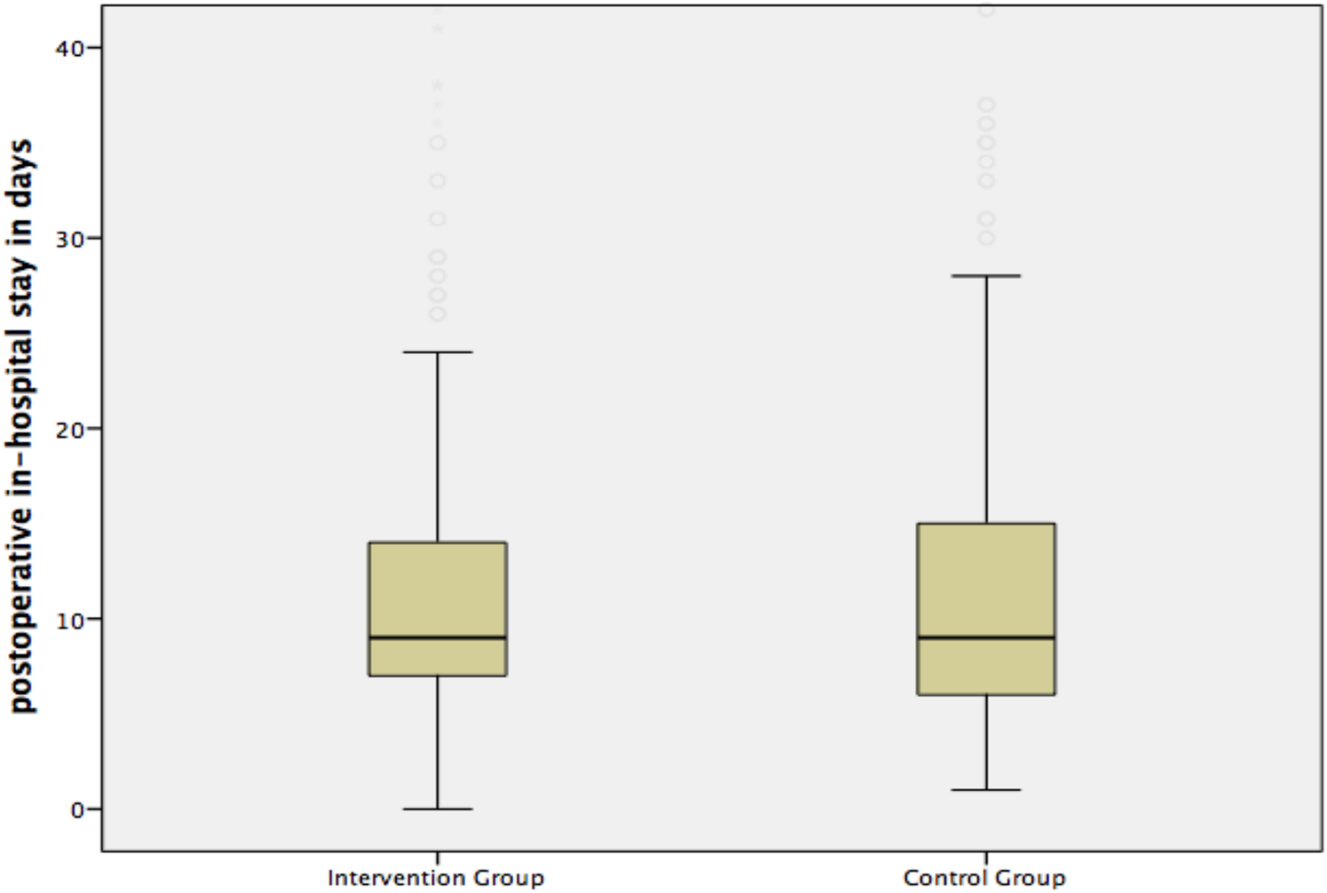
Median postoperative length of in-hospital stay. **(A)** The median length of postoperative in-hospital stay was 9 (IQR 7) days in the intervention group and 9 (IQR 9) days in the control group (p = 0.99).

Fig 2 shows the postoperative in-hospital stay: Duration ranged from 0 to 139 days, with a median of 9 days (Interquartile Range, IQR: 7) for all patients. There was no significant difference between both groups (p = 0.99).

### Primary long-term outcome

#### Health-Related Quality of Life at 12 month

At baseline mean global health-related quality of life in the intervention group was 53.95 (95% CI 49.12–58.77) in women and 68.79 (95% CI 65.90–71.69) in men. In the control group, the global HRQoL was 50.41 (95% CI 44.88–55.93) in women and 65.37 (95% CI 62.23–68.52) in men. There were no statistical differences between the groups (woman: p = 0.294; men: p = 0.196).

**Fig 3:**
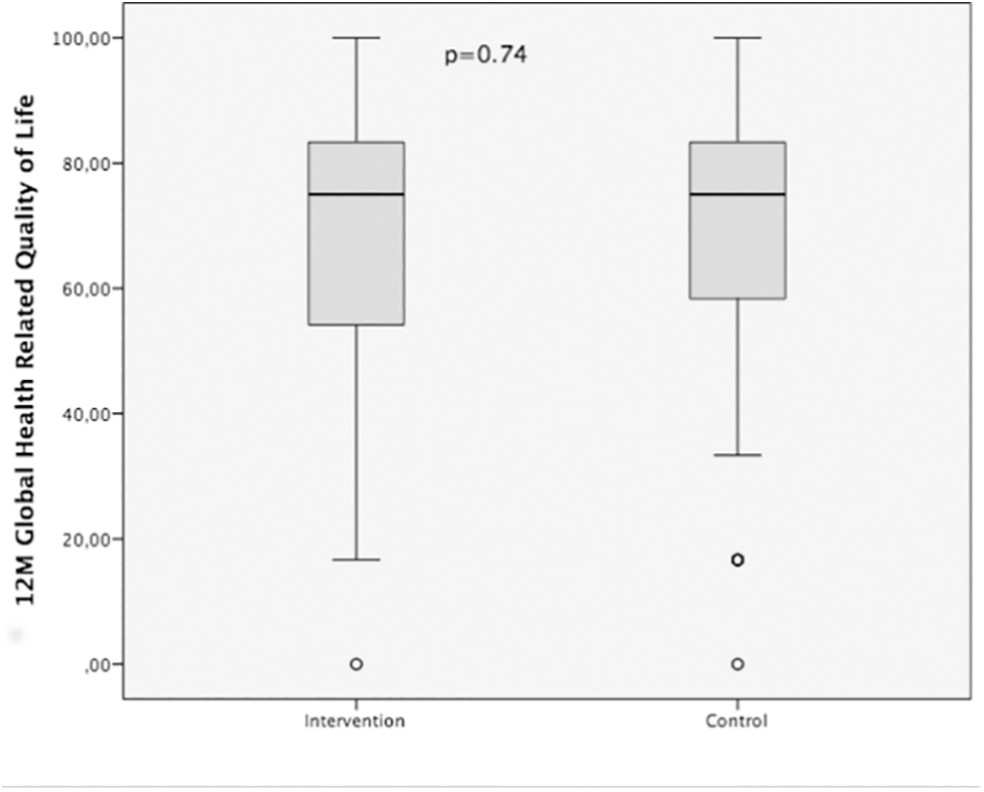
Health-related global quality of life after 12 months for intervention and control groups. HRQoL 12 months after surgery was comparable in intervention and control groups (all patients p = 0.74).

After 12 months, questionnaires were sent to the patients. In the intervention group, 229 questionnaires were sent back, versus 221 questionnaires in the control group. The EORTC QLQ_C30 questionnaire was completed in n = 227 in the intervention group and n = 216 in the control group. Mean global HRQoL after 12 months was 69.27 (95% CI 66.45–72.14) in the intervention group and 69.79 (95% CI 66.84–72.74) in the control group (p = 0.74) (Fig 3). The global HRQoL after 12 months of women in the intervention group was 61.07 (95% CI 55.42–66.71) compared to 63.27 (95% CI 58.28–68.25) in the control group (p = 0.54). Males in the intervention group had a mean global HRQoL after 12 months of 72.49 (CI95% 69.34–75.65) compared to 71.71 (95% CI 68.21–75.20) in the control group (p = 0.94). There was no significant difference between the global HRQoL 12 months after surgery in the intervention and in the control group (all patients: p = 0.74; women: p = 0.54, men: p = 0.94).

**Fig 4:**
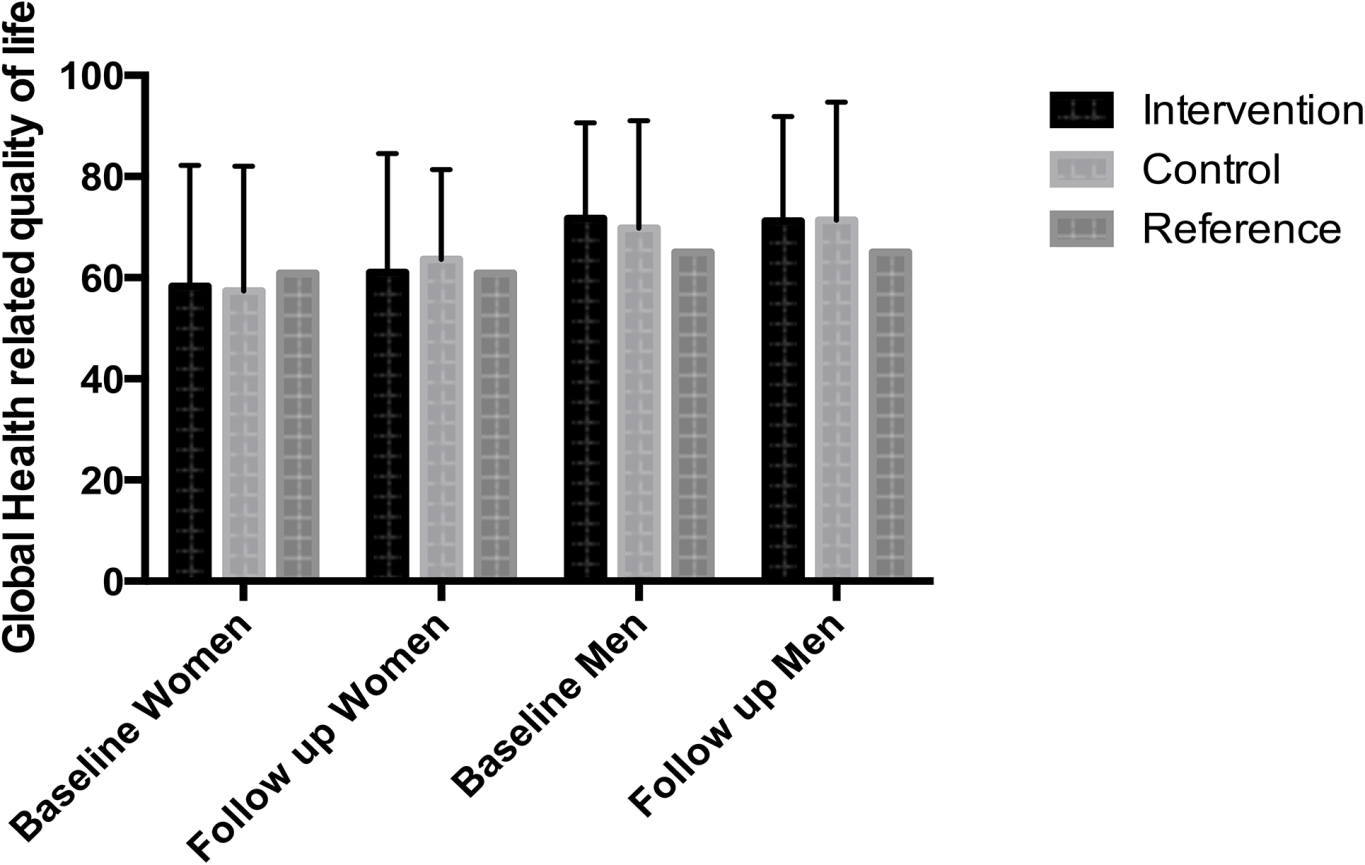
Course of HRQoL. Comparing the baseline and follow-up values for health-related quality of life only for patients who survived and answered the 12 months questionnaire (n = 418), there were neither clinical relevant nor statistical significant differences between baseline and 12 months for HRQoL (intervention women: p = 0.58; men: p = 0.49; control women: p = 0.16; men = 0.29). Compared to age and gender-adjusted reference values for the German population [18], men showed better global health-related quality of life than the reference population with clinical relevance. In contrast, women quoted a lower score of global HRQoL but without clinical relevance.

Compared to age and gender-adjusted reference values for the German population [18], men showed better global health-related quality of life than the reference population. In contrast, women quoted a lower score of global HRQoL without clinical relevance (< 5 points) (Fig 4).

### Secondary outcomes

#### Postoperative morbidity

**Fig 5:**
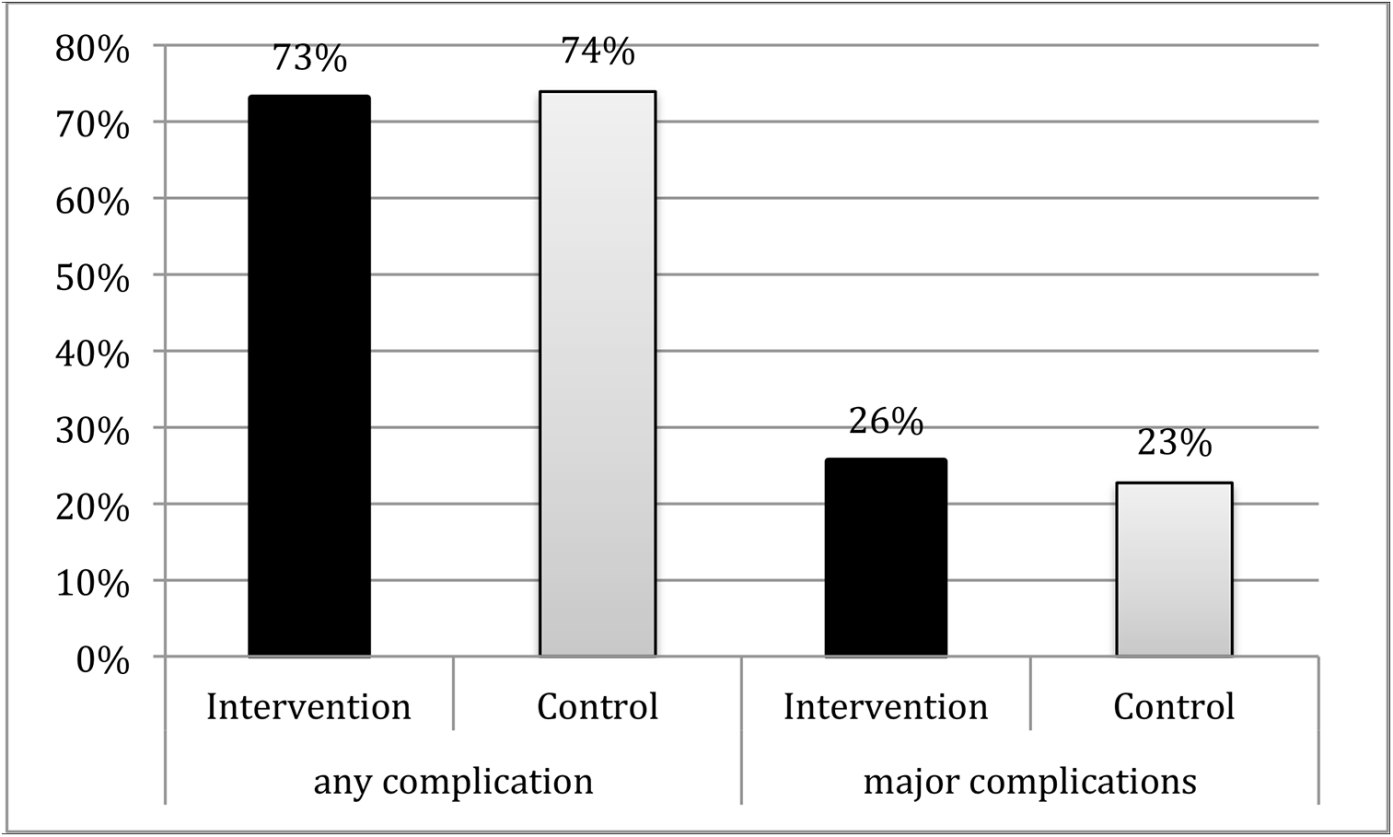
Perioperative complications. Overall complications occurred in 74% of the patients. There was no difference between intervention and control group (p = 0.79). Major (Clavien Grade III and higher) perioperative complications occurred in 24% of the patients. There was no difference between the groups (p = 0.41).

In total, 479 (74%) patients had at least one complication within postoperative hospital stay (Fig 5 and Table 3). Of these, 157 (24%) were classified as major complications according to the Clavien Scale (Fig 5).

**Table 3:** Short-term postoperative outcomes.

Surgical outcomes	Intervention	No Intervention	P
	N = 326	N = 326	
Any complications	238(73%)	241 (73.9%)	0.86^#^
Delirium	36 (11.0%)	30 (9.2%)	0.52^#^
Other neurological complications	7 (2.1%)	8 (2.5%)	1.00^#^
Acute kidney failure, oliguria	21 (6.4%)	13 (4.0%)	0.22^#^
Infections and sepsis	51 (15.6%)	46 (14.1%)	0.66^#^
Cardiopulmonary	104 (31.9%)	97 (29.8%)	0.61^#^
Hemorrhage requiring transfusion	22 (6.7%)	8 (2.5%)	***0*.*01*** ^#^
Anemia requiring transfusion	53 (16.3%)	46 (14.1%)	0.45^#^
Anastomotic leakage	15 (4.6%)	11 (3.4%)	0.42^#^
Intestinal obstruction	7 (2.1%)	9 (2.8%)	0.61^#^
Wound rupture abdominal	16 (4.9%)	8 (2.5%)	0.09^#^
Postoperative pain 1st postoperative day (n = 628)	234 (75.2%)	261 (82.3%)	***0*.*03*** ^#^
ICU stay	132 (40.7%)	124 (38.6%)	0.63^#^
ICU stay > 1 day	93 (29.6%)	89 (28.6%)	0.79^#^
Return to the OR	36 (11%)	39 (12.0%)	0.81^#^
*Depression at discharge (GDS) n = 386*			
No depression	159 (83.2%)	161 (82.6%)	0.86^#^
Risk for depression/Manifest depression	32(16.8%)	34 (17.4%)	

OR: operating room, LOS: length of hospital stay, cardiopulmonary: respiratory insufficiency, angina, myocardial infarction, arrhythmia, lung edema, pulmonary embolism; excluded: pneumonia; SD: standard deviation, IQR: interquartile range.

#: χ2-Test

The most frequent complications were cardiopulmonary complications (30.8%), followed by anemia (15.2%) and infections (14.9%) (Table 3). Occurrence and severity of complications were comparable in both groups, although severe hemorrhage occurred significantly more often in the intervention group (6.7% vs. 2.5%; p = 0.01). Delirium screening was positive in 66 (10.1%) patients with no difference between the intervention and control group (p = 0.52). Due to postoperative complications, 75 (11.5%) patients had to return to the operating theatre.

#### Postoperative stress: Mobilization, PONV and postoperative pain

Patients in the intervention group reported less pain on the first postoperative day (75.2% vs. 82.3%, p = 0.03) (Table 3). There were no differences regarding mobilization within the first 24 hours (69.2% vs. 70.4%, p = 0.73), or PONV within the first five days (52.8% vs. 56.4%, p = 0.39). Further, there was no difference in the GDS between intervention and control groups at discharge (p = 0.86).

#### Readmission

The readmission rate within 90 days was slightly higher for patients in the intervention group (62/245 = 25.3% vs. 59/248 = 23.8%, p = 0.70). In-hospital length of stay at readmission was shorter than in the standard of care group without reaching statistical significance (Median LOS 6.5; IQR: 11 days vs. 10; IQR: 10 days; p = 0.22).

#### Mortality

**Fig 6:**
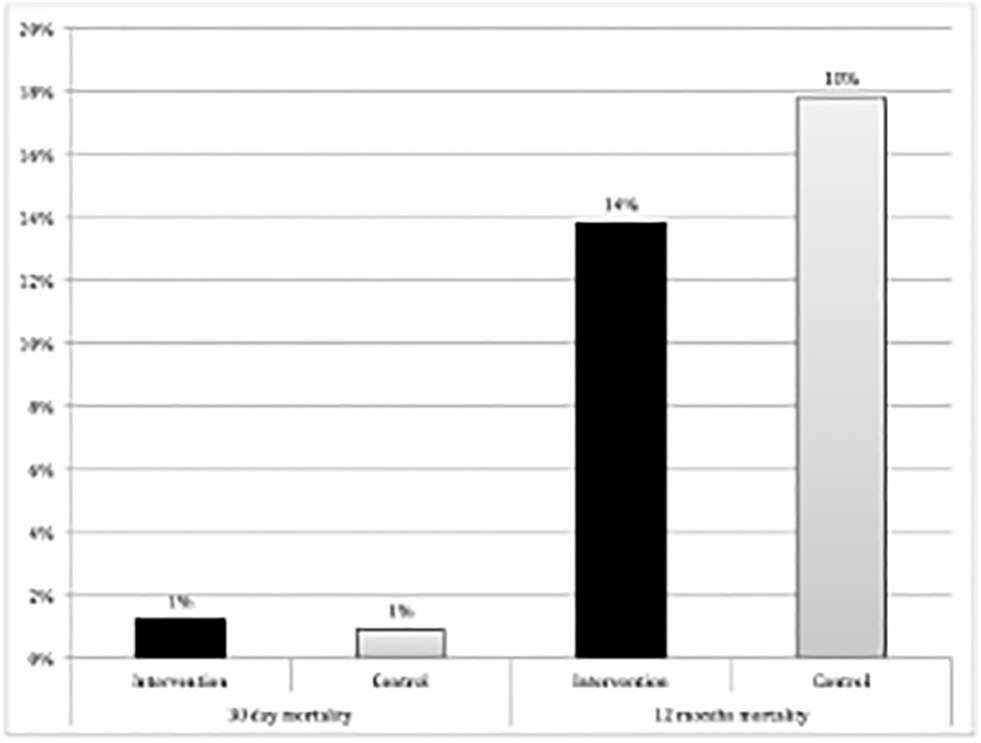
Mortality within 30 days and within one year after surgery for intervention and control groups (p = 0.75 and p = 0.19 respectively). Within 30 days after surgery, 4 patients in the intervention and 3 patients in the control group had died (p = 0.75). One year after surgery, 45 patients in the intervention group compared to 58 patients in the control group had died (p = 0.19).

Ten patients (1.5%) died before hospital discharge. Seven of these (1.1%) died within 30 days after surgery. In the intervention group, 6 (1.8%) patients died compared to 4 (1.2%) in the control group (Fig 6). At follow-up one year after surgery, 103 patients had died (15.8%).

**Fig 7:**
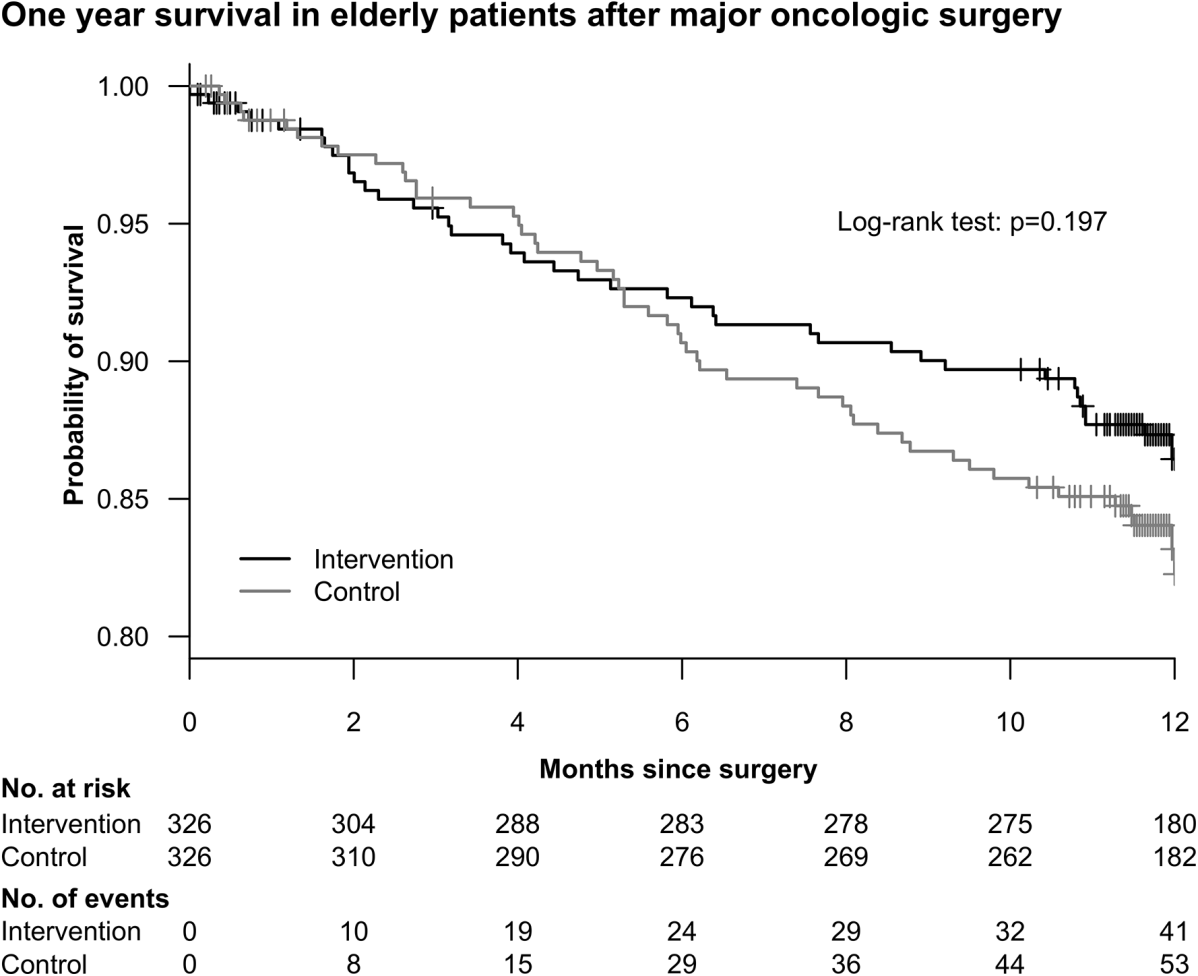
Kaplan Meier Estimation of Survival in intervention and control groups. Patients were followed up to one year after surgery. After 12 months 103 (15.8%) patients had died. Mortality did not differ between intervention and control groups (log rank p = 0.197).

The overall mortality did not differ significantly between the two groups (Log-Rank-test p = 0.197) (Fig 7).

#### Predictors for length of in-hospital stay

The intervention was not significant in robust regression analysis for primary endpoint LOS (-0.15; 95% CI -10.5–0.76; p = 0.75), whereas the pre-operative malnutrition,a delayed Time Up and Go test, increased length of anesthesia, severity of surgery, as well as postoperative major complications had a significant influence on postoperative LOS (S1 Table).

#### Predictors for HRQoL at 12 month

The intervention was not significant in multivariate analysis for primary endpoint global HRQoL (EORTC QLQ C30), whereas the pre-operative global HRQoL, CCS, ASA, MMSE, severity of surgery, as well as postoperative major complications had the most significant influence on the one-year HRQoL (S2 Table).

#### Course of HRQoL

Completed baseline and 12 months follow-up EORTC QLQ C-30 questionnaires were available for 418 patients. In women, the mean difference from baseline global HRQoL to global HRoL 12 months after surgery was -2.63 ± 27 in the intervention group, and – 6,16 ± 25 in the control group (p = 0.37). In men, the mean difference score was -0.48 ± 24 in the intervention group compared to -1.62 ± 25 in the control group (p = 0.71). Between the groups, there were no clinical or statistical significant changes. Merely the women in the control group showed a small clinical decline (5–9 points difference) [16]. Detailed information of clinical changes (small, moderate, large) is provided in S1 Fig.

## Discussion

To our knowledge, this was the first randomized controlled trial to assess the effect of patient empowerment in elderly patients undergoing major cancer surgery. In the present study, patient empowerment did not affect postoperative LOS or long-term HRQoL but did affect the patients’ short-term well-being, such as postoperative pain.

In prior investigations providing detailed information about surgical and anesthetic procedures pre-operatively could diminish perioperative fear and anxiety, and was associated with a shorter in-hospital LOS in particular study settings [8,40,41]. However, previous trials on patient education prior to surgery have provided conflicting results: Shuldham et al.[12] and Guo et al. [42] both showed an even longer LOS in cardiac-surgical patients receiving pre-operative patient education in terms of information. Other trials found a reduction in length of stay after pre-operative patient education in terms of physical training before knee and hip replacement surgery [40]. However, Cochrane meta-analyses could not find strong evidence to support the recommendation for pre-operative interventions as patient education [8,10,43,44]. In the present study, a reduction in postoperative pain on the first postoperative day was found. Postoperative pain management may reduce the surgical stress response, organ dysfunction, and improves gastrointestinal motility, thereby facilitating early mobilization [45]. Furthermore, effective postoperative pain relief without use of opioids may reduce the incidence of postoperative nausea and vomiting followed by lower incidence of wound ruptures, as well as facilitating mobilization. Postoperative delirium is also induced by postoperative pain and/or stress [46,47].

The failure to reach significance may be due to the overall short LOS in our study population. We hypothesized a reduction of in-hospital stay from 12 to 10 days due to patient empowerment. In reality, the median LOS for patients was surprisingly low with median 9 days in both groups. During the last decades, in-hospital LOS has been reduced due to various reasons, not the least based on economic pressure [48].

Occurrence and frequency of postoperative complications did not differ between the intervention and the control groups. As anticipated, complications influenced the postoperative LOS significantly. Postoperative complications influence clinical outcomes including patients’ well-being and the postoperative mortality [49]. Further, they have important effects on long-term survival and the patients’ ability to recover to pre-operative levels of independency [44].

The high rate of overall and major complications in this study is comparable to other study results with elderly cancer patients [49,50]. We believe that this is due to the comprehensiveness of our record: we prospectively recorded all events requiring treatment beyond the routine as complications by asking patients, nurses, physicians, as well as patient records. Postoperative delirium is a seldomly recognized complication [49], and in this study, the postoperative delirium rate of 10% was lower than expected. However, both centers participating in this study have well established postoperative delirium screening and therapy protocols, which has been shown to reduce delirium rates [51]. Furthermore, at enrollment all patients had a Mini Mental State of 24 or higher, and might have been less vulnerable for cognitive disturbances than frail patients with pre-existing cognitive impairment [52].

Despite the high rate of complications, the mortality rate within the first 30 days was surprisingly low. In 2012, the perioperative mortality rate in the EUSOS study was as high as 4% for an unselected inpatient surgery within the first seven days following surgery [53]. For Germany, the in-hospital rate was still 2.5%. In the present study, the patient cohort is a selected study population 65 years old or older in addition to a severe disease, i.e. cancer followed by major onco-surgery. Thus, the anticipated mortality rate was higher than the observed rate of 1.1%. Aside from a selection bias, possible reasons might include the participation in the present RCT, which was combined with daily visits of all patients.

Patient Reported Outcomes (PRO) are established outcome parameters [54]. Empowering patients can achieve a better health-related quality of life in patients [55,56]. In the present study, both study groups showed an improvement in global HRQoL close to the 5-point improvement we assumed for this study. The control group had a trend for higher decline. The intervention showed no influence on the HRQoL one year after surgery. Identifying other determinants of global as well as functional and symptom related parts of HRQoL would be subject of a sub analysis.

Regarding hospital LOS, the present study revealed that amongst others, pre-operative nutritional state and physical functional capacity were independent in elderly cancer patients [57]. Many factors are associated with in-hospital LOS after elective onco-surgery: age, severity of surgery, blood loss, surgical volume, and surgical complications have been previously described as influencing variables [49,58,59]. Interestingly, impairments in functional capacity were not reflected in ASA scores or in the performance status. This strengthens the assumption that the routinely used pre-operative assessments are unsatisfactory for elderly patients, although published data concerning this issue are still conflicting [49,52,60,61]. A pre-operatively conducted geriatric assessment could identify patients who are functionally impaired, and interventions for improving their physical status may be indicated in order to enhance their postoperative recovery [49].

### Limitations

Additional to the limitations already mentioned above, there might be a selection bias in our study population. The study protocol and battery of questionnaires, including cognitive and physical tests, were time consuming and sometimes even stressful for the participants. Further, the intervention started a day prior to surgery. Several study participants were hesitant to be enrolled in a study due to the severity of their diagnosis and the impending major surgery. A considerable number of patients expected to be completely healed after surgery and therefore saw no need in further interventions. Even more patients felt sometimes too weak, or were simply not able to keep their diary due to their postoperative physical and/ or cognitive condition. In particular, patients transferred to the ICU postoperatively were often not able to keep the diary. Hence, contrary to other studies showing beneficial effects of diary keeping [62], perioperative patient empowerment is limited in elderly patients. The patients that refused to participate could differ significantly from the study population in terms of mental and physical health, leading to another possible limitation of this study. We cannot eliminate the possibility that the daily visits and close interaction of study-staff with the participants influenced the primary endpoints, compared to patient settings in the daily routine. Due to the study protocol there was no reference group to facilitate direct comparisons.

Further, the EORTC QLQ-C30 Questionnaire was not specifically designed for elderly patients. The now available EORTC QLQ –C15-PAL, which is specifically designed for elderly patients, did not exist when the present study was designed and conducted [63].

Alternatively, the study has several strengths: the prospective design, the homogenous patient collective, the high recall in long-term follow-up, and the reasonable size of the study population.

## Conclusion

Patient empowerment in terms of additional pre-operative information failed to shorten postoperative in-hospital stay or improve global HRQoL in elderly patients undergoing major onco-surgery. Postoperative length of stay in elderly cancer patients is mainly influenced by pre-operative nutritional, cognitive and functional state, magnitude of surgery, length of general anesthesia, as well as postoperative complications. Patient empowerment can enhance quality of care in regards to pain, and since over-treatment of pain is particularly harmful for elderly patients, patient safely can thus be improved. Pre-operative information was received well by patients who were cognitively and physically fit.

Pre-operative comprehensive geriatric assessment might be beneficial for this high-risk patient group, helping to provide a tailored perioperative management aimed at reducing cognitive and physical barriers, and ultimately improving postoperative outcome.

## Supporting Information

S1 CONSORT Checklist CONSORT Checklist. (PDF)

S1 Fig Changes in global HRQoL from baseline to follow-up. Detailed clinical changes in global HRQoL from pre-operative to 12 months follow-up. There were no differences between both groups (p = 0.17). The majority showed no clinical relevant changes in global HRQoL.
(TIF)

S1 Protocol Clinical Trial Protocol (English). (PDF)

S2 Protocol Clinical Trial Protocol (German). (PDF)

S1 Table Robust regression analysis with respect to the primary endpoint postoperative length of hospital stays. Variables entered: Intervention no vs. yes, gender, age in years, tumor site (genito-urinary vs. gastrointestinary, nutrition state (MNA: manifest malnutrition vs. normal/risk for malnutrition), Timed up and go: > 21 sec vs. < 20 sec; Severity of surgery^§^ (major+ vs. moderate/major), major complications (no vs. yes), length of anesthesia in minutes, school degree (> high school vs < high school). Pre-operative malnutrition and a delayed time up and go test, increased length of anesthesia and severity of surgery as well as postoperative major complications had a significant influence on postoperative LOS.
(TIF)

S2 Table Perioperative predictors of global health-related quality of life (EORTC QLQ C30) one year postoperative. (Variables entered on step 1: Intervention yes vs. no, gender, age in years, ASA state I/II vs. III/IV, Charlson Comorbidity Score per point, tumor site (genito–urinary vs. gastrointestinary), nutrition state (MNA: normal/risk for malnutrition vs. manifest malnutrition), Timed up and go: < 20 sec vs. > 21 sec; Severity of surgery^§^ (moderate/major vs. majorplus), pre-operative global health-related quality of life per point, major complications (no vs. yes), Depressions (none vs. manifest), Fatigue (no/mild vs. severe), Activities of daily living per point, Mini Mental State per point). Pre-operative global HRQoL, CCS, ASA, and MMSE, intraoperative severity of surgery as well as postoperative major complications had the most significant influence on the one-year HRQoL.
(TIF)

## Abbreviations

ADL: Activities of Daily Living
ASA: American Association of Anesthesiologists
BFI: Brief Fatigue Inventory
CAM-ICU: Confusion Assessment Method for intensive Care Units
CGA: Comprehensive Geriatric Assessment
ECOG: Eastern Cooperative Oncology Group
EORTC: European Organisation of Research and Treatment of Cancer
EORTC QLQ-C30: European Organisation of Research and Treatment of Cancer 30 –Item Core Quality of Life Questionnaire, version 3.0
GDS: Geriatric Depression Scale
HRQoL: Health-Related Quality of Life
IADL: Instrumental Activities of Daily Living
ICU: Intensive Care Unit
LOS: Length of in-hospital stay
MMSE: Mini Mental State Examination
MNA: Mini Nutritional Assessment
NRS: Numeric Rating Scale
NUDESC: Nursing Delirium Scale
PACE: Preoperative Assessment of Cancer in the Elderly
PONV: Postoperative Nausea and Vomiting
POSSUM: Physiological and Operative Severity Scoring system for enumeration of Mortality and morbidity
PRO: Patient Reported Outcomes
RCT: Randomized Controlled Study
TUG: Timed Up and Go—test
