# Supplementary figures and images for: Patient Empowerment Improved Perioperative Quality of Care in Cancer Patients Aged ≥ 65 Years – A Randomized Controlled Trial
Source: PLoS One. 2015 Sep 17;10(9):e0137824. doi: 10.1371/journal.pone.0137824 (PMC4574984; doi:10.1371/journal.pone.0137824)

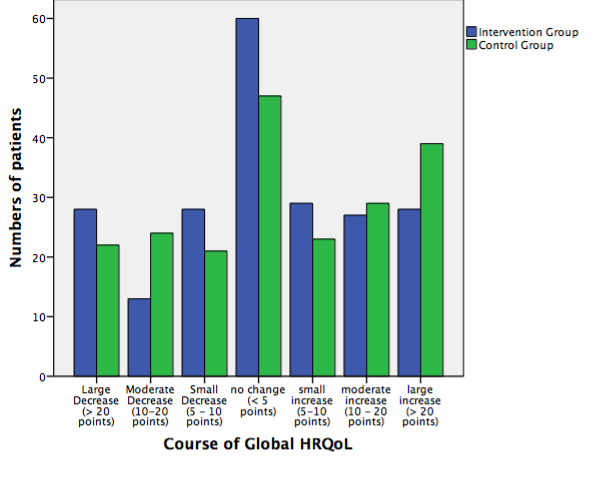

Supplement: S1 Fig — Detailed clinical changes in global HRQoL from pre-operative to 12 months follow-up. There were no differences between both groups (p = 0.17). The majority showed no clinical relevant changes in global HRQoL. (TIF) [file pone.0137824.s002.tif]

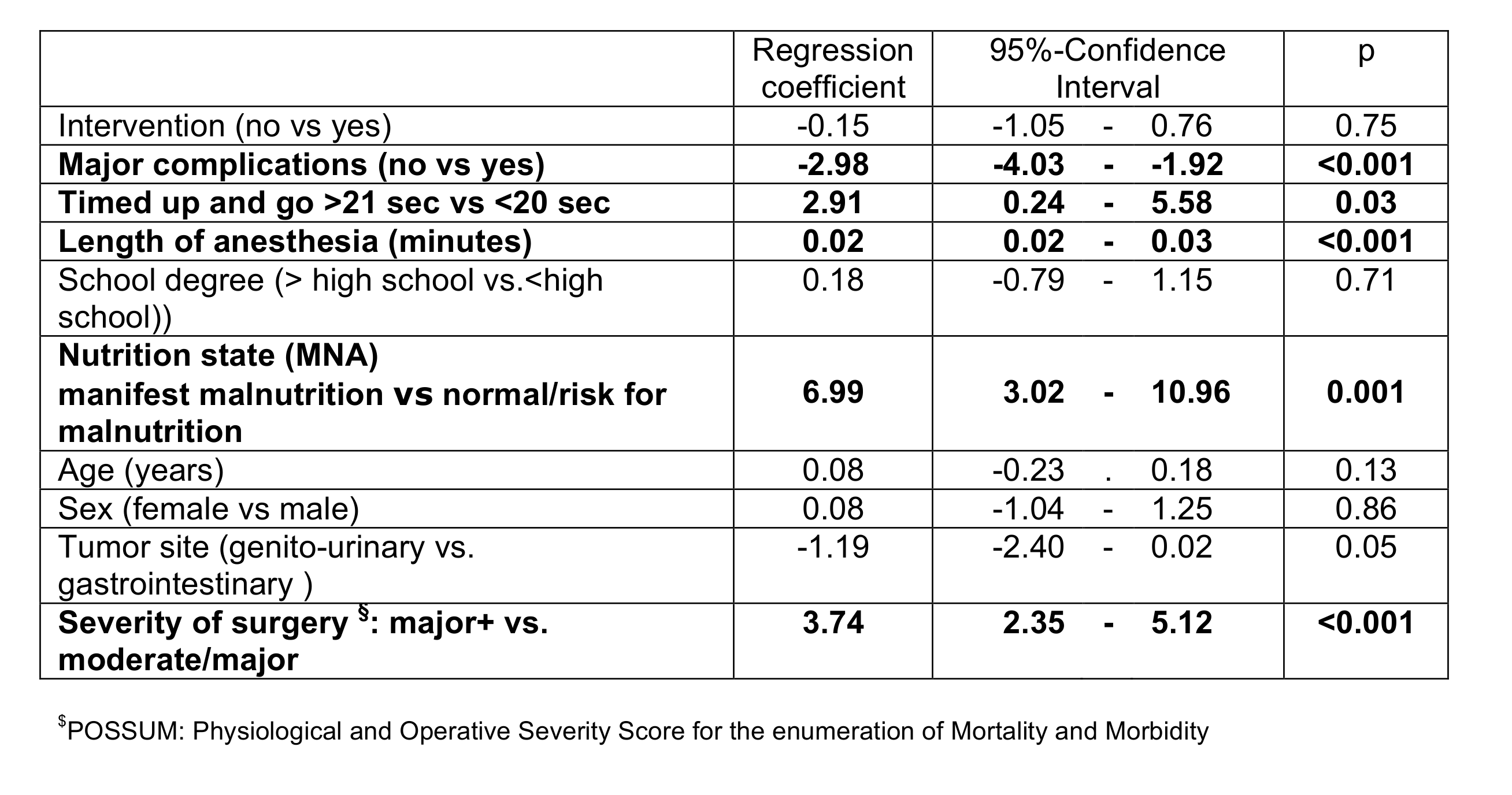

Supplement: S1 Table — Variables entered: Intervention no vs. yes, gender, age in years, tumor site (genito-urinary vs. gastrointestinary, nutrition state (MNA: manifest malnutrition vs. normal/risk for malnutrition), Timed up and go: > 21 sec vs. < 20 sec; Severity of surgery§ (major+ vs. moderate/major), major complications (no vs. yes), length of anesthesia in minutes, school degree (> high school vs < high school). Pre-operative malnutrition and a delayed time up and go test, increased length of anesthesia and severity of surgery as well as postoperative major complications had a significant influence on postoperative LOS. (TIF) [file pone.0137824.s005.tif]

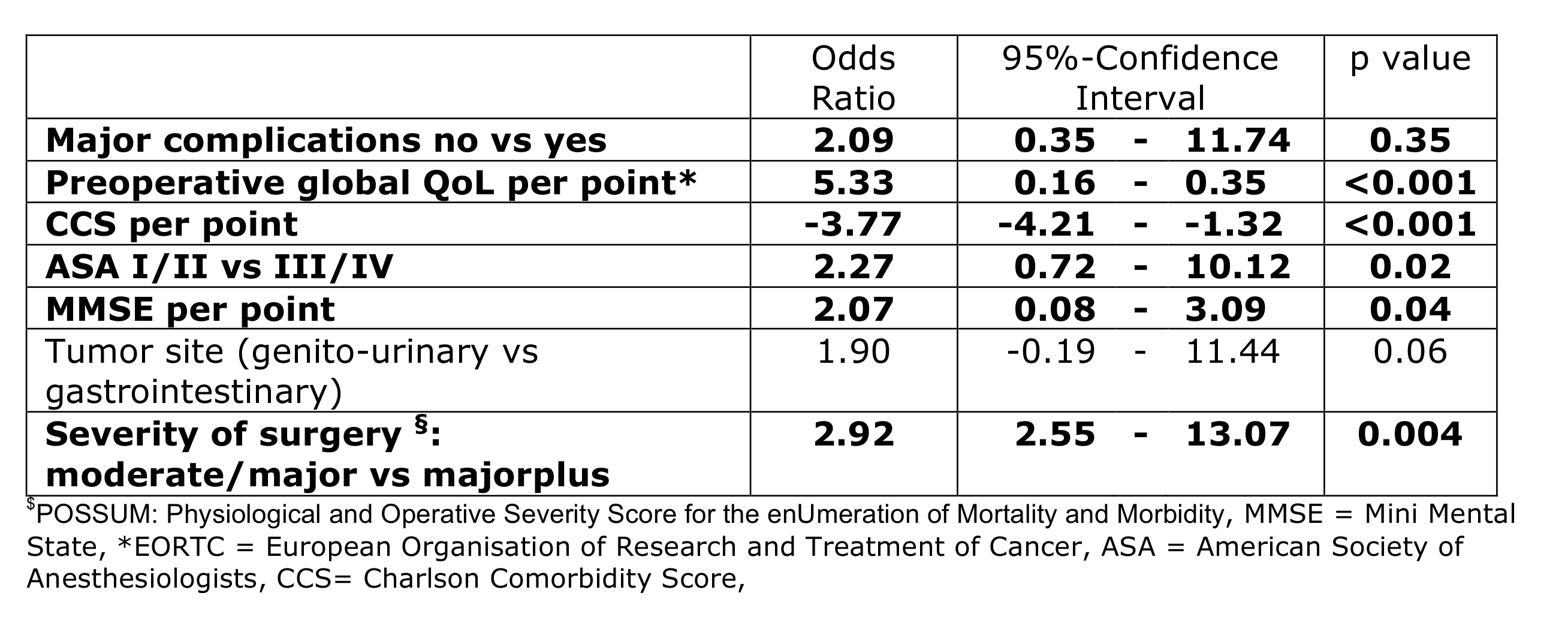

Supplement: S2 Table — (Variables entered on step 1: Intervention yes vs. no, gender, age in years, ASA state I/II vs. III/IV, Charlson Comorbidity Score per point, tumor site (genito–urinary vs. gastrointestinary), nutrition state (MNA: normal/risk for malnutrition vs. manifest malnutrition), Timed up and go: < 20 sec vs. > 21 sec; Severity of surgery§ (moderate/major vs. majorplus), pre-operative global health-related quality of life per point, major complications (no vs. yes), Depressions (none vs. manifest), Fatigue (no/mild vs. severe), Activities of daily living per point, Mini Mental State per point). Pre-operative global HRQoL, CCS, ASA, and MMSE, intraoperative severity of surgery as well as postoperative major complications had the most significant influence on the one-year HRQoL. (TIF) [file pone.0137824.s006.tif]
