# Supplementary material for: Patient Empowerment Improved Perioperative Quality of Care in Cancer Patients Aged ≥ 65 Years – A Randomized Controlled Trial
Source: PLoS One. 2015 Sep 17;10(9):e0137824. doi: 10.1371/journal.pone.0137824 (PMC4574984; doi:10.1371/journal.pone.0137824)
Supplement: S1 Protocol — (PDF) [file pone.0137824.s003.pdf]

Approval for consultation by the Ethics Committee for the conduction of a medical-scientific project, which does not include the clinical testing of a drug.

1. TITLE OF STUDY	Patient empowerment and risk-adjusted treatment to improve outcome in the elderly after gastrointestinal, thoracic or urogenital cancer surgery (PERATECS)<br>A prospective, randomized, controlled trial
2. IRB application number	(Assigned by IRB)
3. Decisions of other IRB in the same concern	Nonexistent
4. The study and its aims (hypotheses, separated into main and secondary hypotheses).	The objectives of this randomized, controlled study are to avoid physical, psychological and social unfavorable postoperative limitations through active involvement of the patient (patient empowerment) and thus to enhance the rehabilitation process and to achieve a postoperative improved quality of life for the elderly.<br><br>**<u>Main objectives</u>**<br>The aims of this study are to investigate whether patient empowerment, including information and decision-aids adapted to the risk and the need of the elderly patients, can improve outcome in an evidence-based modular pathway in terms of<br>a) Shortened hospital stay by a reduced postoperative complication rate (short-term perioperative risk)<br>b) Quality of life (long-term perioperative risk)<br>in elderly patients compared to control patients receiving standard of care.<br><br>**<u>Secondary objectives</u>**<br>A risk-adapted patient empowerment can improve outcome in evidence-based modular pathway in terms of<br>(c) Reduced rates of short-term postoperative complications<br>(d) Reduced numbers of readmission<br>(e) Stress reduction, in terms of less postoperative pain, earlier postoperative mobilization less postoperative nausea and vomiting,<br>(f) Reduced mortality rate one year after discharge<br>(g) Improvement of the Geriatric Depression Scale (GDS)<br><br>**Primary (multiple) endpoints:**<br>1. Time to fulfill discharge criteria in days<br>2. Qualtiy of life<br><br>**Secondary endpoints.**<br>1. Postoperative complications<br>2. Readmission rate<br>3. Stress due to postoperative pain, lack of mobilization, postoperative nausea and vomiting<br>4. Mortality rate one year after discharge<br>5. Depression, assessed by the Geriatric Depression Scale (GDS)
5. Rationale	**<u>Medical rationale</u>**<br>Two-thirds of all solid tumors occur in patients aged 65 years or older and most cancer-related deaths occur in this age group. Elderly patients receive substandard perioperative care and have high perioperative mortality.<br>The aims of this study are to investigate whether patient empowerment, including information and decision-aids adapted to the risk and the need of the elderly patients, can improve outcome in an evidence-based modular pathway in terms of<br>c) Shortened hospital stay by a reduced postoperative complication rate (short-term

perioperative risk)
d) Quality of life (long-term perioperative risk)

in elderly patients compared to control patients receiving standard of care

Elderly people are the fastest growing segment of the population in the Western world[1]. Two-thirds of all solid tumors occur in patients aged 65 years or older and most cancer-related deaths occur in this age group. [2]. Cancer incidence has been reported to be 11 times higher in this age group than in people aged less than 65 years[2]. Although surgical techniques have improved, the high perioperative morbidity remained unchanged over the last decade[3]. Further, fewer elderly patients undergo resection for i.e. colorectal cancer than their younger counterparts[4, 5], The outcome of major surgery in elderly patients could be similar to that in younger people if careful selection and appropriate attention to detail is exercised. The importance of appropriate treatment does not diminish with age, because inadequate treatment in older cancer patients is associated with poor survival and poor quality of life[6-9].

Reasons for the increased mortality after major abdominal surgery are various. Host responses to surgical stress and infection are altered with aging and age-related increases in susceptibility to postoperative complications and morbidity have been noted [10] resulting in prolonged hospitalization and worse convalescence [11-15].

It was only in the last two decades that the prognostic importance of increased co-morbidities in elderly patients and risk factors as powerful prognostic indicators were realized [16]. The lack of a reliable instrument for risk assessment is crucial [17] as there is evidence that fit elderly cancer patients are frequently under-treated on the one hand and a number of "poor surgical risk" candidates are offered an ineffective, painful and unnecessary surgical procedure on the other hand. [18].

Patient Empowerment

During the past 40 years view on the patient changed from passive recipient of health – care to a consumer who demands autonomy. Theoretical benefits of involving well-informed patients in health care decisions include better compliance with treatment and increased satisfaction in both, the patient and the health-care professional [19].

The WHO defines empowerment as a " prerequisite for health" [20]. Evidence shows that health outcomes in patient empowerment strategies take place through several pathways: directly- through improvement in individal decision-making efficacy, diesease complication management and improved health behaviors – and indirectly through strengthened support groups, caregiver empowerment, enhanced satisfaction with provider/patient relationships and better access and efficient use of health services, enhanced self-education and improved mental health outcomes [11-15]. Intervention based on evidence-based medicine could improve the postoperative outcome and the quality of life after surgery [21].

Empowerment interventions, i.e. consisting of support groups, educational opportunities, patient decision-making, have been actively pursued in diabetes care and other chronic diseases as chronic obstructive pulmonary disease, end-stage renal disease and in cancer [22-24]. Elderly cancer patients are under representated [25]

Information and social support

Elderly patients´ desire for information and shared-decision making is not taken serious enough[26]. Therefore, it is urgently required to improve information adequately prepared for elderly patients prior and after surgery to motivate the patients to participate actively in the process of healing [19, 27].

Patients with high **social support** reported better emotional functioning and less serious stress reactions than patients with a low degree of social support[28]. A limited social support structure often increases the risk of readmissions and disparity in outcomes [28]. Possibilities to increase the social support are a better and earlier incorporation of self-help association or honorary working persons.

Quality of life(QoL)

	Advances in surgical, anesthetic and intensive care techniques have allowed the treatment of septuagenarians, octogenarians and even centenarians. But very little is known about the **quality of life (QoL)** of these patients.<br>Undergoing surgery has an immediate impact on several aspects of HRQoL. Patients experience pain, nausea, vomiting, and constipation that diminish physical function and the ability to work or carry out social activities. The long-term consequences of surgery vary according to the type and extent of procedure, For example, after rectal surgery and formation of a stoma, patients experience sexual, urinary, and gastrointestinal dysfunction In patients with advanced malignancy, global QoL, and social functioning subscale, were found to be independently prognostic of survival after controlling for age, performance status, and metastatic site [29-34].<br>A reduced QoL or poor functional status prior to surgery has found to be correlated with a poor QoL after ICU stay [35]. Additional, up to 30% of elderly patients developed depression and anxiety [36]<br>Stress reduction exercise respectively mindfulness-based stress reduction and guided imagery reduced depression as well as fatigue and pain in other studies[22, 37-39] [40, 41]. Studies on professionally –lead support groups showed also improvement in quality of life[42-44]. However, the majority of these studies were conducted in middle-aged women with breast cancer, particularly in early-stage[39].<br><br>**Methods:**<br>All patients > 65 years fulfilling the inclusion criteria will be screened. After decision for surgery in established tumor conferences, patient will be randomized to an intervention or standard of care group in a modular evidence-based clinical pathway. In all patients an early risk evaluation (PACE/GDS/EORTC.QLQ 30) will be performed and patients´ motivation to actively support the rehabilitation in the hospital will be checked. Postoperatively, patients were visited daily up to and including the fifth postoperative day to document pain, mobilization, nutrition and possibly delirium. On the day of discharge, patients are visited once more.<br><br>In the intervention group, patients will be empowered to participate actively in the process. The patient will receive information and decision-aids Patients will be empowered to participate in preoperative (brief advice on health- and lifestyle-related risk) and postoperative (pain management, delirium prevention, mobilization), as well as post-discharge intervention strategies (contact information for physical, psychological, and social support).<br>.<br>Three and twelve months later, the patients will be questioned again regarding quality of life and depression.<br><br>**Number of patients:**<br>The trial will be conducted at Charité University-Medicine Berlin, University Hospital of the LMU Munich, and at the University Hospital Heidelberg/Mannheim. Director of the study is Univ.-Prof. Dr. C. Spies, Department of Anesthesiology and Intensive Care Medicine, Charité University-Medicine Berlin, CCM/CVK.<br><br>Each Center should recruit 230 patients each year to reach 690 patients altogether.<br><br>**Trial duration:**<br>Overall 2 years , Duration per patient: 1 year.
6. Which of the following provisions shall apply<br>a) Medical Devices Act<br>b) Radiation	e) Data Protection Act

Protection Ordinance<br>c) X-Ray Ordinance<br>d) Genetic Engineering Act<br>e) Data Protection laws	
7. If appropriate: Identification and characterization of investigational products	N/A
8. Main results of pre-tests or reasons for not implementing these	N/A
9. Main topic and results of previous studies/applications of the product to be tested in the study	The positive influence of patient empowerment through social and informational support in diseases such as diabetes mellitus and other serious chronic diseases such as COPD, renal failure and cancer has been shown in previous studies. In the previous studies, patient aged 65 years and older are under-represented and shared decision-making not adequately investigated.<br>Likewise there were no specific risk surveys in patients ≥ 65 years. However, several studies approved by the ethics committee were conducted in our department, including:<br>• „Preventing nicotine-associated diseases by smoking cessation“ [45, 46]<br>• “Importance of lifestyle as a risk for surgery”[47-49]<br>• „PART (Patient Active Role Training)”
10. Description of the proposed measures /methods of investigations and any deviations from the normal clinical routine	The perioperative treatment for all patients is carried out after the published and certified (according to DIN EN ISO9001) standards of the participating hospitals. There is no study-specific adaption of the measures or examinations including routine blood draws. This includes the anesthetic preparation; induction and maintenance as well as postoperative monitoring in the recovery room or on the intensive care units and the indicated invasive monitoring of patients. The patients’ blood samples will be investigated in accordance of the routine diagnostics of the participating hospitals. The discharge for the recovery room or the intensive care units is based on the fulfillment of discharge criteria according to the Aldrete score.<br>For the study, patients will be filling out additional questionnaires:<br>The preoperative and postoperative screening in the control group include the completion of several questionnaires: preoperatively the completion of various questionnaires regarding physical performance, comorbidities, nutritional status, quality of life, depression and life style risks (nicotine, alcohol disease). The total time is 40 minutes. Postoperatively, patients were visited daily up to and including the fifth postoperative day to document pain, mobilization, nutrition and possibly delirium. These visits take approximately 15 minutes to complete. On the day of discharge, patients are visited once more (about 15 minutes).<br>Three and twelve months later, the patients will be questioned again regarding quality of life and depression (about 20 minutes).<br>Patients in the intervention group will be visited and informed by trained members of the corresponding supporting groups at both times, pre- and postoperatively. In addition, these patients receive a kind of patient diary. The completion of the diary should not take more than 15 minutes a day to complete.

11. Assessment and consideration of the foreseeable risks and inconveniences of study participation against the expected benefits for the participants and future patients	Patient safety is ensured by the doctors of the hospital involved in the clinical routine in accordance with the implemented Standard Operating Procedures (SOP), ensuring a high level of evidence-based medicine.
a. Predictable therapeutic benefit to the study participants	The foreseeable benefits for the study participants in the intervention group consist primarily in a preoperative risk analysis and a risk management through active involvement of the patient (patient empowerment) to achieve an eventual reduction of short-term complications and stress prevention. This therapeutic benefit is not yet shown sufficient in patients over the age of 65 years, but it is expected to be determined in this study, in accordance with previous results.<br>The study participants in the control group receive any disadvantages in relation to standard of care and being treated according the standards of the participating hospitals without any further additional value.
b. Foreseeable future medical benefit for ill people	The implementation of informative and social support for participatory decision-making in multimodal, multidisciplinary treatment modules would also be in the future of profit for older patients with cancer.
c. Risks and burdens to the study participants (all listed in detail).	The preoperative and postoperative screening in the control group include the completion of several questionnaires: preoperatively the completion of various questionnaires regarding physical performance, comorbidities, nutritional status, quality of life, depression and life style risks (nicotine, alcohol disease). The total time is 40 minutes. Postoperatively, patients were visited daily up to and including the fifth postoperative day to document pain, mobilization, nutrition and possibly delirium. These visits take approximately 15 minutes to complete. On the day of discharge, patients are visited once more (about 15 minutes).<br>Three and twelve months later, the patients will be questioned again regarding quality of life and depression (about 20 minutes).<br>Patients in the intervention group will be visited and informed by trained members of the corresponding supporting groups at both times, pre- and postoperatively. In addition, these patients receive a kind of patient diary. The completion of the diary should not take more than 15 minutes a day to complete.<br>From our view, there are no risks for patients arising from participation in this trial.
12. Risk control	The attending physicians of the participating hospitals will care for the patients until discharge and the study team will visit the participating patients daily after enrollment up to and including the fifth day after surgery and on the day before discharge.
13. Termination criteria	Under the following conditions occurs early withdrawal of patients from the study according to the termination criteria:<br>The following may be considered reasons:<br>• personal wish of the patient<br>• any other situation in which the study physicain thinks further participation in the trial would not be in the best interest of the patient<br>• significant protocol violations<br>• Subsequent occurrence of an exclusion criterion<br>Premature end of the study or discontinuation of the entire study can be considered because of the following circumstances:<br>• Decision of the study director at unacceptable risks under the risk-benefit balance<br>• new (scientific) (no longer exists positive benefit-risk assessment) findings during the term of the clinical trial, which could endanger the safety of study participants

14. Number, age and gender of participating subjects	The study will be carried out at 3 tertiary university hospitals (Berlin, Munich, Heidelberg) under the direction of Prof. Dr. C. Spies, Department of Anesthesiology and Intensive Care, Charité, University Medicine Berlin.<br>It is anticipated that approximately 230 patients per center, a total of 690 patients, will be enrolled. With a 30% loss during the study period a total of 480 patients (240 patients per group) is expected.<br>Participating patients are of both gender and aged 65 years and older.
15.Statistics: primary analysis plan and rationale for number of products	**Description of the primary efficacy analysis and population**<br>Exploratory data analysis of the primary and secondary endpoints: frequencies, median, 25% -75% percentiles, arithmetic mean, standard deviation SD, and corresponding 95%-confidence intervals.<br>Wilcoxon (Mann-Whitney) tests for the first and second primary endpoints (error of the 1st kind Bonferroni-adjusted). QoL: Mantel-Haenszel-tests for stages of change, Mann-Whitney U-test for Decisional Balance, Processes of Change and Self-efficacy and subjective health.<br><br>**Secondary Endpoints**<br>Kaplan-Meier estimates for survivals, Log-Rank-test to proof for differences in survivals between intervention and control, multivariate Cox' proportional hazard regression including interesting risk factors and to check for confounders, Chi-square-test in contingency tables, Mantel-Haenszel-test to proof the number of risk behaviors changed in detail between the groups.<br>In addition, logistic regression models including suspected risk factors and controlled for confounding variables will be conducted. This is particular important for mortality as response in order to proof the impact of the defined intervention multivariately. Model fit will be proved by goodness of fit as well as calibration criteria.<br>Statistical test for secondary endpoints should be understood as constituting exploratory data analysis, such that no adjustments for multiple testing will be made.<br><br>**Safety**<br>Not applicable (no pharmacological treatment)<br><br>**Sample Size**<br>Sample size calculation with respect to the second primary endpoint in a worst -case scenario. Bonferroni-adjusted error of the 1st kind α = 0.025 two-sided, power 80%, and the following quantities [1]: Global quality of life score according to EORTC QLQ C-30 [mean (SD)]: 68.82 (15.51) [before intervention], 73.12 (13.73) [after 12 months follow-up].<br>Supposing the nonparametric Wilcoxon (Mann-Whitney) rank-sum test, a sample size of 235 patients per group will be needed.<br>With 12 days versus 10 days (time to fulfill discharge criteria from the hospital) and an effect size of 0.5 (according to own studies) for the first primary, a sample size of 83 patients per group would follow (α = 0.025 two-sided, power 80%, calculations using nQuery Advisor® Release 7.0, Stat. Solutions Ltd. & South Bank, Crosse's Green, Cork, Ireland).<br><br>**Charité**<br>Preoperative anesthetic clinic: Patients / year = 40.000<br>Patients with scheduled cancer surgery ≥ 65 years<br>To be assessed for eligibility: n = 776<br>To be analyzed: n = 230 (115 per group)<br><br>**Other centers**<br>230 each centre and year<br><br>Altogether 690 patients after one year of intervention.<br><br>With ca. 30% loss to follow-up, we obtain **n = 480** patients at year 2 altogether, i.e. **n1 =**

	**n2 = 240** patients per group.
16.<br>Subject selection criteria	**Inclusion criteria:**<br>• Written informed consent by the patient<br>• Patients aged ≥ 65 years with gastrointestinal (upper gastrointestinal, colorectal), thoracic and urogenital (prostate gland, uterine) malignancies scheduled for surgery<br>• The cancer diagnosis should be confirmed by histology respectively cytology before inclusion in the study. If the diagnosis cannot be confirmed, the patient has to be excluded from the study.<br>• Mini Mental State > 23<br>• Life-expectance > 2 months<br>• No participation in another clinical study during the duration of the trial<br><br>**Exclusion criteria are:**<br>• No informed consent by the patient<br>• Failure to obtain consent<br>• Emergent patients<br>• Outpatients,<br>• Patients aged < 65 years,<br>• Participation in another clinical study<br>• Patients undergoing emergency surgery<br>• Patients with unclear history,<br>• Patients with concurrent malignancies<br>• Mini-mental state < 23<br>• Inability to give written informed consent (patients with acute, life-threatening conditions inability to understand and complete the questionnaires, severe medical condition making an interview impossible)<br>• Life-expectance < 2 months
b. **Information of participants**	see appendix nr 2<br><br>Before starting the study every patient will be informed of the nature of the study, the goals, and the possible risks orally and in written form.
c. **Informed consent form**	See appendix nr 3<br>Every patient has to declare written content to participate in the study after being informed of the nature, the goals and the possible risks of the study. To the patient has to be provided sufficiently enough time to decide and to clear out open questions. The informed written consent will be firmed by the patient and by the Investigator dated handwritten. If the patient is able to consent the participating but cannot firm handwritten it has to be confirmed handwritten by a witness after oral clarification of facts.<br>The content and form of the patient information and consent will be presented for appraisal to the Ethics Committee (see appendix). Both will be firmed two times to handout one specimen to the patient. The other one stays with the Investigator.
d. if applicable Information and written consent of legal representatives	N/A

17. Arrangements of recovery of study participants (notice? Newspaper ads etc.)	Study participants may be only patients who undergo surgery at the Charité or the other participating university hospitals. These patients are asked preoperatively to participate in the study.<br>There will be no participants recruited actively or by recruiting ads.<br>.
18. If appropriate: Reasons for inclusion and presentation of therapeutic benefit for individuals who are under age and/or not capable of giving consent.	N/A
19. Relationship between patient and investigator	The study physician is not also the attending physician, but usually employed in the same hospital. Patients are free to decide whether they will participate or not. Their decision will not influence their clinical care.
20. If appropriate, Statement to include potentially dependent on the sponsor or investigator persons	N/A
21. Methods of avoiding simultaneous Enrolment in other Trials	During screening the patient will be asked if he is participating in another clinical study. Additionally the offered information for the patient will contain the advice to announce participating in another study. If the patient participated the time before being recruited for this study the fact that inhibits participation in our trial is if the last examination of the previous study has not been realized or was realized less than three months ago.
22. If appropriate remuneration or reimbursement of study participants (Amount, reason?)	N/A
23. If necessary, plan for further treatment and medical care for patients after the end of the study	The duration of study is limited to the hospitalization of the patient. Furthermore, a survey after three and twelve months is carried out regarding quality of life. Since the patient is discharged only after the fulfillment of defined criteria discharge from the hospital, the post treatment during the study is guaranteed. Care beyond the end of the study is not provided.
24. If necessary,.: insurance of study participants (insurers, insurance coverage, duration of insurance)	The patients are insured by the treating institution as part of their clinical management. The study doctor is insured by the employer's liability insurance, Charité - University Medicine Berlin against liability claims
25. If necessary .: documentation procedures (reference to CRF sheets possible)	The documentation of the study data will be realized with an electronic Case Report Form (ECRF). The tagging of the person who reports the record will be implemented to assure the validity of the data.<br>The study software contains checks for plausibility, consistence and ranges, an audit-trail and a management system for queries.<br>Data will be recorded online and directly transferred into the data bank of the study server.

	Transfer from workplace comuter to the server will be via safe connection (SSL_encoding).<br>All patient related data will be stored pseudonymous and the study software creates for every new patient automatically a pseudonym. The unequivocal assignment of every patient to his pseudonym results from a paper print, which has to be secured safely.
26. If appropriate; Description how to document the health status of healthy indivuduals involved in the study	No healthy individuals will participate in the study. All patients will be 65 years or older and suffering from cancer. The decicion for surgery as part of the therapy was made independently before inclusion in the study.
27. If necessary: Measurement to find, to document and to communicate adverse events	N/A<br>.
28 Procedures to protect the confidentiality of the stored data, documents and, if necessary, samples, giving the encryption of the data of study participants	The records in the eCRF is stored only pseudonyms. The patient identification list that allows to connect between a patient and a record is accessible only to authorized persons and must be kept closed. The encoding scheme for the use of pseudonyms is automatically generated from the electronic system secuTrial.<br><br>The database is safeguarded against unauthorized access by established security procedures; appropriate backup copies of the database and related software files will be maintained.
29. Declaration of data protection compliance	The provisions of the Data Protection Act are complied with. See the name and signature of the / the applicant. See Appendix no. 6 written report for information the Data Protection Officer of the Charité.<br>The study doctors will use personal information for purposes of administering and conducting the study and for purposes of research and statistical analysis.<br>For any disclosure of data of participants in the study, the data will be provided with a code number (pseudonyms of the data). To the code key that allows it to bring the study-related data with the patient in combination, only the study doctor and his staff have access. All records on which patient can be identified will be kept strictly confidential.<br>All persons involved in the study are informed with respect to familiarity, medical confidentiality and the policies of the GCP.
30. Names and addresses of the devices that are integrated as a study center or laboratory in the study, and the study director and the investigators	Director of the trial<br>Prof. Dr. Claudia Spies<br>Universitätsklinik für Anästhesiologie mit Schwerpunkt operative Intensivmedizin,<br>Charité – Universitätsmedizin Berlin,<br>Charité Campus Mitte<br>Charitéplatz 1, 10117 Berlin<br>Charité Campus Virchow Klinikum<br>Augustenburger Platz 1, 13353 Berlin<br>Tel. +49 30 450 551001/02<br>Fax: +49 30 450 551909<br>anaesthesie-virchow-klinikum@charite.de<br><br>**Study doctors**<br>OÄ Dr. Maren Schmidt<br>Klinik für Anästhesiologie und operative Intensivmedizin,<br>Charité – Universitätsmedizin Berlin,

	Charité Campus Virchow Klinikum<br>Augustenburger Platz 1, 13353 Berlin<br>Tel: +49 (0) 30 450 651197<br>Fax: +49 (0) 30 450 551909<br>E-Mail: maren.schmidt@charite.de<br><br>Dr. Jan Philip Breuer<br>Klinik für Anästhesiologie und operative Intensivmedizin,<br>Charité – Universitätsmedizin Berlin,<br>Charité Campus Mitte<br>Charitéplatz 1, 10117 Berlin<br>Charité Campus Virchow Klinikum<br>Augustenburger Platz 1, 13353 Berlin<br>Tel.: +49 (0) 30 450 631093<br>Fax: +49 (0) 30 450 531 911<br>E-Mail: philip.breuer@charite.de<br><br>Biometrics<br>**Statistician**<br>Prof. Dr. Klaus-Dieter Wernecke<br>Biometrie und Statistik<br>Wildensteiner Str 27<br>10318 Berlin<br>Tel: +49 30 50158763<br>Fax: +49 30 50158906<br>E-Mail: kdwernecke@sostana.com<br><br>**Participating study centers:**<br><br>**Ruprecht-Karls-Universität Heidelberg**<br>Universitätsklinikum Heidelberg<br>Universitätsklinik für Anästhesiologie<br>Prof. Dr. med. Eike O. Martin, (geschäftsführender Direktor)<br>Im Neuenheimer Feld 110<br>69120 Heidelberg<br>Tel.: 06221 56 6350<br>Fax.: 06221 56 5345<br><br>Universitätsklinik Mannheim<br>Klinik für Anästhesiologie und operative Intensivmedizin<br>Prof. Dr. med. Dr. h.c. Klaus van Ackern (Klinikdirektor)<br>Prof. Dr. med. Grietje Beck (Stellvertreterin des Direktors)<br>Theodor-Kutzer-Ufer 1-3<br>68167 Mannheim<br>Telefon 0621/383-2614<br>Telefax 0621/383-3806<br><br>**Ludwig-Maximilians - Universität München**<br>Universitätsklinik München<br>Klinik für Anaesthesiologie der Universität München<br>Prof. Dr. Bernhard Zwissler<br>OA Prof. Dr. M. Thiel<br>Marchioninistr. 15<br>81377 München<br>Telefon ++49 - (0) 89 - 7095 - 4551<br>Telefax ++49 - (0) 89 - 7095 – 8885<br>.

31. About the suitability of the inspection body, particularly on the appropriateness of existing resources and facilities there as well as to the conduct of the trial staff available and experience in conducting similar studies	The Charité and the other participating university hospitals are maximum care hospitals with a 24-hour call to patient care. The participating departments have extensive experience in conducting clinical trials
32. Agreement on access by the auditor / principal investigator / principal investigator of the data and the principles about the publication 32.	The records of patient data is kept strictly confidential and transfer of data is carried out only in pseudonymous form.<br>The study doctors only have access to personal data, as it is necessary to conduct the study.<br>The head of the clinical trial has access to all the data.<br>The publications are aimed regardless of the outcome of the study in a peer-reviewed journal.
33. Study financing	Grant: German Cancer Aid DKH-108474<br>Duration: 2 years<br>The clinical routine is by the patient's health insurance.
a. Funding Organization	Deutsche Krebshilfe e. V.; Buschstr. 32; 53113 Bonn
b. Amount of estimated costs per participant and total	The amount of costs is determined by the German Cancer Aid in the review process. Third-party account is set up..
c.Amount of reimbursement per patient and total	N/A

References:

1. http://www.destatis.de/basis/d/gesu/DRG *Statistik.php*.
2. Coebergh JW, *Epidemiology in Europe*. Eur J Cancer, 2001. **37**(Suppl 7): p. 175 - 227.
3. Janssens JP, K.K., *Pneumonia in the very old*. Lancet Infect Dis, 2004. **4**: p. 112 - 124.
4. Iscoe N., T.P., Gort E, Wu K,, *Cancer surgery in the elderly*. Ann Oncol, 1998. **9**(suppl 3): p. absr 109.
5. Irvin TT, *Prognosis of colorectal cancer in the elderly*. Br J Surg, 1988. **75**: p. 419-421.
6. Goodwin J.S., S.J.M., Hunt W.C., *Determinants of Survival in Older Cancer Patients*. J Natl Cancer Inst, 1996. **88**(15): p. 1031- 1038.
7. Mangano DT, *Perioperative Cardiac Morbidity*. Anesthesiology, 1990. **72**: p. 155-184.
8. Seymour DG, P.R., *Postoperative complications in the elderly surgical patient*. Gerontology, 1983. **29**: p. 262- 270.
9. Audisio RA, *The surgical risk of elderly patients with cancer*. Surg Oncol, 2004. **13**(4): p. 169 - 173.

10. Ono S, A.S., Tsujimoto H, Ueno C, Mochizuki H. , *Increased monocyte activation in elderly patients after surgical stress*. Eur Surg Res, 2001. **33**: p. 33 - 38.
11. Spies CD, E.V., Szabo G, Lau A, von Dossow V, Schoenfeld H, Althoff H, Hegenscheid K, Bohm B, Schroeder T, Pfeiffer S, Ziemer S, Paschen C, Klein M, Marks C, Miller P, Sander M, Wernecke KD, Achterberg ER, Kaisers U, lenzenhuber E, Volk HD, *Intervention of the Neuroendocrine-Immune Axis and Postoperative Pneumonia Rate in Alcoholic Patients.* . Am J Resp Crit Care 2006. **174**: p. 408 - 414.
12. Spies CD, S.C., Weiß-Gerlach E, Neuner B, Neumann T, von Dossow V, Schenk M, Wernecke KD, Elwyn G. , *Preferences for shared decision making in chronic pain patients compared to patients in premedication visit.* . Acta Anaesthesiol Scand, 2006. **50**: p. 1019 - 1026.
13. Otter H, M.J., Bäsell K, Heymann von C, Vargas Hein O, Böllert P, Pattariya J, Behnisch I, Wernecke KD, Konertz W, Loening S, Blohmer J, Spies *Validity and reliability of the DDS for severity of delirium in the ICU*. Neurocrit Care, 2005. **2**(150 - 158).
14. Heymann C von, S.H., Sander M, Ziemer S, Grubitzsch H, Spies C, *Clopidogrel-Related Refractory Bleeding after Coronary Artery Bypass Graft Surgery: A Rationale for the Use of Coagulation Factor Concentrates?* heart Surgery Forum, 2005. **8**: p. 39 - 41.
15. Martin J, P.A., Franck M, Wernecke KD, Fischer M, Spies C, *Practice of sedation and analgesia in German intensive care units: result of a national survey*. Crit Care, 2005. **9**: p. 117 - 123.
16. Ponzetto M, M.B., Maina P, Rosato R, Ciccone G, Merletti F, Rubenstein LZ, Fabris F, *Risk factors for early and late mortality in hospitalized older patients: the continuing importance of functional status*. J Gerontol A Biol Sci Med Sci, 2003. **43**: p. M59 - M63.
17. Pope D, R.H., Gennari R, Corsini G, Maffezini M, Hoekstra HJ, Mobarak D, Sunouchi K, Stotter A, West C, Audisio RA, *Pre-operative assessment of cancer in the elderly (PACE): A comprehensive assessment of underlying characteristics of elderly cancer patients prior to elective surgery*. Surgical Oncology 2007. **15**: p. 189 - 197.
18. Audisio RA, B.F., Gennari R, Jaklitsch MT, Koperna T, Longo WE, Wiggers T ,Zbar AP,, *The surgical management of elderly cancer patients: recommendations of the SIOG surgical task force*. Eur J Cancer, 2004. **40**: p. 926 - 938.
19. Gaston CM, M.G., *Information giving and decision-making in patients with advanced cancer: A systematic review*. Social Science & Medicine, 2005. **61**: p. 2252 - 2264.
20. Neuhauser D., *The coming third healthcare revolution: personal empowerment*. Qual Manag Health Care, 2003. **12**: p. 171 - 184.
21. Peterson MG, C.-M.J., Finerty E., Graziano S., King S., Sculco TP, *Effectiveness of best practice implementation in reducing hip arthroplasty length of stay*. J Arthroplasty, 20088. **23**(1): p. 69 - 73.
22. Golant M., A.T., Martin C., , *Managing cancer side effects to improve quality of life: a cancer psychoeducation program*. Cancer Nurs, 2003. **26**(1): p. 37 - 44.
23. Mishra SI et al., *Improving breast cancer control among Latinas: evaluation of a theory-based educational program*. Health Education& Behavior, 1998. **25**(5): p. 653 - 670.
24. Davison, B.J., Degner LF, *Empowerment of men newly diagnosed with prostate cancer*. Cancer Nurs, 1997. **20**(3): p. 187 - 196.
25. Wetzels R., H.M., Van Weel C., Grol R., Wensing M., *Interventions for improving older patients' involvement in primary care episodes*. Cochrane Data Base Syst Rev, 2008(4).
26. Mazur DJ., H.D., *The effect of physicians's explanation on patient's treatment preferences: five -year survival data*. Med Decis Making, 1994. **14**: p. 255 - 258.

27. Warburton DE, N.C., Bredin SS, *Health benefits of physical activity: the evidence*. CMAJ, 2006. **14**(174): p. 801 - 809.
28. Ringdal GI, R.K., Jordhoy MS, Kaasa S, *Does social support from family and friends work as a buffer against reactions to stressful life events such as terminal cancer ?* Palliat Support Care, 2007. **5**: p. 61 - 69.
29. Dancey J, Z.B., Osoba D, , *Quality of life scores: an independent prognostic variable in a general population of cancer patients receiving chemotherapy. The National Cancer Institute of Canada Clinical Trials Group*. Qual LifeRes, 1997. **6**: p. 151 - 158.
30. Kaasa S., M.A., Lund E., , *Prognostic factors for patients with inoperable non-small cell lung cancer, limited disease. The importance of patients' subjective experience of disease and psychosocial well-being*. Radiother Oncol, 1989. **15**: p. 235-242.
31. Efficace F., B.A., Smit E.F., Lianes P., Legrand C., Debruyne C., Schramel F., Smit H.J., Gaafar R., Biesma B., Manegold C., Coens C., Giaccone G., Van Meerbeck J.,, *Is a patient's self-reported health-related quality of life a prognostic factor for survival in non-small-cell lung cancer patients? A multivariate analysis of prognostic factors of EORTC study 08975*. Ann Oncol, 2006. **17**: p. 1698-1704.
32. Maisey N.R., N.A., Watson M., Allen M.J., Hill M.E., Cunningham D., *Baseline quality of life predicts survival in patients with advanced colorectal cancer*. Eur J Cancer, 2002. **38**: p. 1351-1357.
33. Camilleri-Brennan J., S.R., *Prospective analysis of quality of life and survival following mesorectal excision for rectal cancer*. Br J Surg, 2001. **88**(12): p. 1617-1622.
34. Coates A, P.F., Osoba D, *Quality of life in oncology practice: prognostic value of EORTC QLQ-C30 scores in patients with advanced malignancy*. Eur J Cancer, 1997. **33**: p. 1025 - 1030.
35. Merlani P, C.C., Mariotti N, Ricou B, *Long-term outcome of elderly patients requiring intensive care admission for abdominal pathologies: survival and quality of life*. Acta Anaesthesiol Scand, 2007. **51**: p. 530 - 537.
36. Depla MF, P.A., de Lange J, *New residents of residential care homes for the elderly. The problems of elders with mental disorders*. Tijdschr Gerontol Geriatr, 1999. **30**: p. 121 - 128.
37. Daugherty C., e.a., *Perceptions of cancer patients and their physicians involved in phase I trials*. J clin Oncol, 1995. **13**(5): p. 1062 - 72.
38. Houts PS., e.a., *The Prepared Family Caregiver: A Problem-solving Approach to Family Caregiver Education*. Patient Education & Counseling, 1996. **27**(1): p. 63 - 73.
39. Penedo FJ., D.J., Molton I., Gonzalez JS., Kinsinger D., Roos BA., Carver CS., Schneiderman N., Antoni MH., , *Cognitive-Behavioral Stress Management Improves Stress-Management Skills and Quality of Life in Men Recovering from Treatment of Prostate Carcinoma*. Cancer, 2003. **100**(192 - 200).
40. Carlson L.E., S.M., Patel K.D., Goodey E., , *Mindfulness-based stress reduction in relation to quality of life, mood, symptoms of stress and levels of cortisol, dehydroepiandrosterone sulfate (DHEAS) and melatonin in breast and prostate cancer outpatients*. Psychoneuroendocrinology, 2004. **29**(4): p. 448 - 74.
41. Antoni MH., L.J., Kilbourn KM., Boyers AE., Culver JL, Alferi SM., Yount SE., Mc Gregor BA., Arena PL., Harris SD., Price AA., Carver CS.,, *Cognitive-behavioral stress management intervention decreases the prevalence of depression and enhances benefit finding among women under treatment for early-stage breast cancer*. Health Psychol, 2001. **20**: p. 20-32.

42. Goodwin J.S., E.M., Bordelau LJ, Pritchard KI, Trudeau ME, Koo J., Hood N.,, *Health-related quality of life and psychosocial status in breast cancer prognosis: analysis of multiple variables*. J clin Oncol, 2004. **22**(20): p. 4184 - 4192.
43. Kassane DW., L.A., Hatton A., Bloch S., Smith G., Clarke DM., Miach P, Ikin J. , Ranieri N. Snyder RD., , *Effect of cognitive - existentioal group therapy on survival in early stage breast cancer*. J clin Oncol, 2004. **22**(21): p. 4255 - 60.
44. Liebermann M., G.M., Altman T.,, *Therapeutic Norms and Patient benefit: Cancer Patients in Professionally Directed Support Groups*. Group Dynamic, Theory, Research and Practice. **8**(4): p. 265-276.
45. Weiss-Gerlach, E., et al., *Motivation of trauma patients to stop smoking after admission to the emergency department*. Addictive Behaviors, 2008. **33**(7): p. 906-918.
46. Neuner, B., et al., *Socioeconomic factors, hazardous alcohol consumption, and smoking in patients with minor trauma in an inner-city emergency department*. Journal of Emergency Medicine. **In Press, Corrected Proof**.
47. Neuner, B., et al., *Hazardous alcohol consumption and sense of coherence in emergency department patients with minor trauma*. Drug and Alcohol Dependence, 2006. **82**(2): p. 143-150.
48. Schoenfeld, H., et al., *The Effect of Stress-Reducing, Low-Dose Ethanol Infusion on Frequency of Bleeding Complications in Long-Term Alcoholic Patients Undergoing Major Surgery*. The American Surgeon, 2007. **73**: p. 192-198.
49. Spies, C., Neuner B., Neumann T., et al., *Intercurrent complications in chronic alcoholic men admitted to the intensive care unit following trauma*. Intensive Care Med, 1996. **22**(4): p. 286 - 293.

Name und Unterschrift des/der Antragstellers:

Ich versichere hiermit, dass die in diesem Antrag gegebenen Informationen richtig sind. Ich bin der Auffassung, dass es möglich ist, die o.g. Studie in Übereinstimmung mit dem Protokoll, den nationalen Rechtsvorschriften durchzuführen.

Mir ist bekannt, dass ich gemäß §19 Berliner Datenschutzgesetz (BlnDSG) verpflichtet bin, für automatisierte Verarbeitungen personenbezogener und personenbeziehbarer Daten eine Datei- und Verfahrensbeschreibung zu erstellen und diese gemäß §19a dem behördlichen Datenschutzbeauftragten der Charité zur Verfügung stellen muss. Ich bin darüber informiert, dass wenn es sich um ein Verfahren handelt, mit dem Daten verarbeitet werden, die einem Berufsgeheimnis (z.B. ärztliche Schweigepflicht) unterliegen, ich gemäß §5 BlnDSG vor dem Einsatz dieses Verfahrens eine Vorabkontrolle durch den behördlichen Datenschutzbeauftragten der Charité veranlassen muss und ich das Verfahren erst bei positivem Prüfergebnis anwenden darf.

Name: Prof. Dr. Spies

Vorname: Claudia

Adresse: Augustenburger Platz 1
13353 Berlin

Position: Klinikdirektorin der Universitätsklinik für Anästhesiologie mit Schwerpunkt operative Intensivmedizin der Charité - Universitätsmedizin Berlin CCM/CVK

Datum:

Unterschrift:

Appendix:

1. Flow Chart of trial conduction
2. Information for study participants
3. Informed and written consent

Anlage 1 Flow Chart

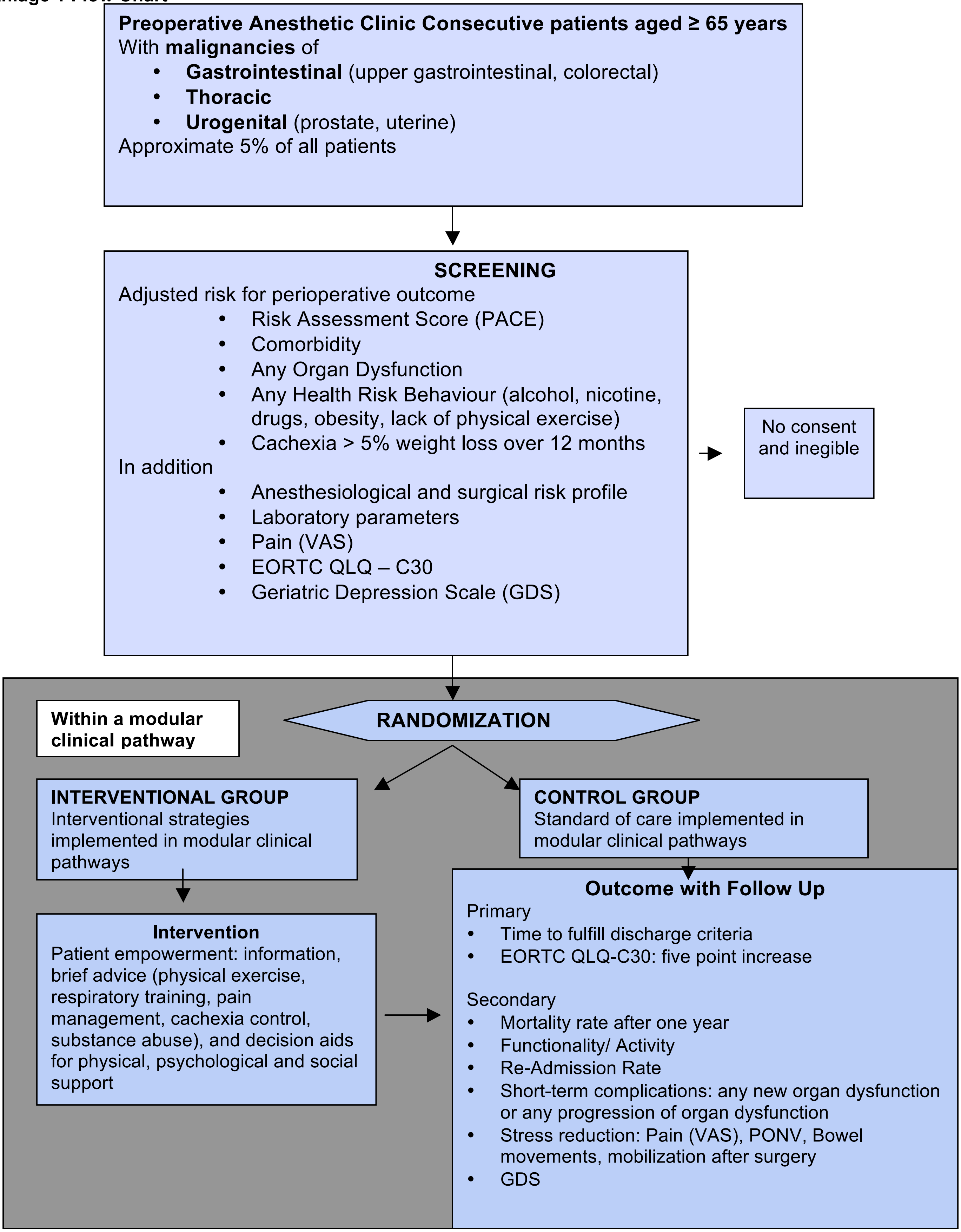

CHARITÉ

CharitéCentrum für Anästhesiologie, OP-Management und Intensivmedizin

Charité - Universitätsmedizin Berlin | D - 13344 Berli

Patientenkleber

Universitätsklinik für Anästhesiologie
mit Schwerpunkt operative Intensivmedizin
CCM / CVK

Klinikdirektorin:
Univ.-Prof. Dr. C. Spies

Zertifiziert nach DIN EN ISO 9001

Campus Virchow-Klinikum
Augustenburger Platz 1
13353 Berlin
Tel: +49 30 450 551-001
Fax: +49 30 450 551909
anaesthesie-virchow-klinikum@charite.de

Campus Charité Mitte
Charitéplatz 1
10117 Berlin
Tel: +49 30 450 531-012
Fax: +49 30 450 531911
anaesth@charite.de

http://www.charite.de/ch/anaest/

Patienteninformation

Version 1.0 vom 25.11.2008

zur Teilnahme an der Studie

„Patientenmitbestimmung und Risiko-gestützte Behandlung zur Verbesserung des Outcomes bei älteren Patienten nach gastrointestinaler, thorakaler und urogenitaler Onkochirurgie"

Kurztitel: PERATECS

Studienleiter: Univ.-Prof. Dr. C. Spies

Sehr geehrte Patientin, sehr geehrter Patient,

Ihnen ist vorgeschlagen worden, an der oben genannten Studie teilzunehmen. Ältere Menschen sind die am schnellsten wachsende Bevölkerungsgruppe in der westlichen Welt. Ebenfalls nimmt die Zahl der Operationen bei älteren Menschen zu. Als Folgen von operativen Eingriffen können bei den genannten Patienten vermehrt Komplikationen wie zum Beispiel Entzündungen im Wundgebiet oder Lungenentzündungen, Herz-Kreislauf-Komplikationen auftreten. Wichtig ist speziell bei älteren Patienten die Bedeutung der Begleiterkrankungen zu erfassen, um spezielle altersbedingte Risikofaktoren festlegen zu können.
Um ein speziell auf ältere Patienten zugeschnittenes Risikoprofil erstellen zu können, werden Ihnen verschiedene Fragebögen ausgehändigt, die zum einen nach Ihren Nebenerkrankungen, Ihrer körperlichen Leistungsfähigkeit sowie Ihrem Ernährungszustand fragen. Aber auch Ihre Lebensqualität vor und nach der Operation wird erfasst.
Eine Teil der Patienten (Studiengruppe) erhält außerdem zusätzlich ein Tagebuch, in dem alle relevanten Informationen zum Verlauf des Krankenhausaufenthaltes verzeichnet sind. Es wird

Ihnen zum Beispiel genau erklärt bis wann Sie vor der Operation essen und trinken dürfen. Aber auch ab wann Sie beispielsweise ab einer bestimmten Schmerzstärke ein zusätzliches Medikament bekommen können. Weiterhin erhält diese Patientengruppe sowohl vor als auch nach der Operation Informationen von Seiten der Selbsthilfegruppen bzw. Patientenvertreter. Diese Information betreffen die Erkrankung an sich, vor allem aber auch die Möglichkeiten, nach der Krankenhausentlassung Unterstützung zu bekommen.
Die andere Hälfte der Patienten, die sog. Kontrollgruppe erhält nur die oben genannten Fragebögen zu Erstellung eines Risikoprofils.
Die Studie findet an der Charité – Universitätsmedizin Berlin, Klinik für Anästhesiologie und operative Intensivmedizin, Campus Virchow-Klinikum und Campus Charité Mitte unter der Leitung von Prof. Dr. Claudia D. Spies statt. Weitere Studienzentren sind das Universitätsklinikum Heidelberg/Mannheim und das Klinikum der Universität München.
Die Studie verfolgt keine kommerziellen Interessen.

Zweck der Studie
Es soll ein speziell auf ältere Patienten zugeschnittenes Risikoprofil erstellt werden.
Der Zweck der Datenerhebung soll sein, eine präoperative Risikoanalyse speziell für ältere Patienten zu erheben, um Möglichkeiten der Risikoreduktion finden zu können.
Weiterhin soll untersucht werden, ob eine vermehrte Information und aktive Einbindung der Patienten in ihre Therapie einen Einfluss auf den Krankheitsverlauf hat.
Der Zweck der Datenerhebung ist die Überprüfung der Hypothese, dass eine vermehrte Information und aktive Einbindung der Patienten in ihre Therapie einen positiven Einfluss auf den Krankheitsverlauf hat.

Freiwilligkeit der Teilnahme

Die Teilnahme an dieser Studie freiwillig und Sie können jederzeit ohne Angabe von Gründen von der Zusage zur Teilnahme zurücktreten, ohne dass Ihnen hieraus Nachteile für Ihre weitere Behandlung erwachsen. Sie erhalten dann in jedem Fall eine Therapie (Behandlung), die dem aktuellen Wissensstand entspricht.

Ablauf und Dauer der Teilnahme
Die von uns geplante Untersuchung findet als so genannte „klinische Studie“ statt.
Wir möchten Sie bitten, vor der Operation einige Fragebögen zu beantworten. Diese Fragebögen enthalten Fragen zu Ihrem Lebensstil, Ihrer Lebensqualität, zu Ihren Begleiterkrankungen und dabei auch Fragen bezüglich Stresses und riskanten Gesundheitsverhaltens. Einige sozioökonomische Fragen werden erhoben, um sie mit dem repräsentativen Gesundheitsumfrage des Robert-Koch-Instituts vergleichen zu können.
Die Befragung vor der Operation nimmt ca. 45 Minuten in Anspruch. An den ersten fünf Tagen nach der Operation werden Sie von Studienmitarbeitern bzw. Doktoranden besucht, die Ihnen Fragen nach Ihrem Befinden stellen. Der Zeitaufwand pro Tag beträgt ca. 10 Minuten. An Ihrem Entlassungstag wird ebenfalls eine kurze Befragung stattfinden, die höchstens 15 Minuten dauert. Außerdem erhalten Sie 3 und 12 Monate nach Ihrer Entlassung Fragebögen zu Ihrer Lebensqualität zugesandt. Die Beantwortung dieser Fragebögen dauert ca. 20 Minuten.

Diese Fragebögen erhalten alle teilnehmenden Patienten.

Weitere Daten, die aufgezeichnet werden, sind allgemeine Angaben wie Name, Anschrift, Telefonnummer, Alter, Geschlecht, Daten zu ihrer physischen Gesundheit wie medizinische Diagnosen und Routine-Laborparameter.
Die Patienten, die in der Studiengruppe sind, erhalten zusätzlich vor und nach der Operation Informationen von Selbsthilfe- und Patientenvertretern. In diesen Gesprächen werden Informationen über die Krankheit, den Aufenthalt im Krankenhaus, vor allem aber auch Möglichkeiten der Unterstützung nach der Entlassung aus dem Krankenhaus gegeben.
Weiterhin erhalten Patienten in dieser Gruppe ein Patiententagebuch, dessen tägliche Führung nicht mehr als 10 Minuten bedarf.
Am Anfang des Tagebuches finden sich allgemeine Informationen, die Sie bitte durchlesen. Anschließend finden sich Fragen, bei denen die entsprechende Antwort angekreuzt wird. Sollten Sie sich nicht ausreichend informiert fühlen, fragen Sie bitte Ihren behandelnden Arzt.
Im Anschluss zu den von Ihnen auszufüllenden Fragen finden sich Anweisungen. Es wird Ihnen zu Beispiel die Information gegeben, dass Sie ab einer bestimmten Schmerzstärke ein zusätzliches Medikament bekommen können. Bitte halten sie in diesem Fall Rücksprache mit Ihrem behandelnden Arzt. Füllen sie die Seiten den Tagen entsprechend nacheinander aus. Um den Umfang des Buches nicht zu groß werden zu lassen, endet es am fünften Tag nach der Operation. Falls Sie länger in unserem Haus sein sollten, füllen Sie bitte die letzten Seiten dann am Entlassungstag aus. Sollten Sie vor dem fünften Tag entlassen werden können Sie die Tage die Sie nicht betreffen überspringen. Beantworten Sie bitte dann an Ihrem Entlassungstag die entsprechende Seite.
Auch in dieser Gruppe wird 3 und 12 Monate nach Entlassung eine Befragung hinsichtlich der Lebensqualität durchgeführt, sofern Sie zustimmen. Auch bei diesen Anrufen bzw. Anschreiben können Sie selbstverständlich jederzeit die Beantwortung unserer Fragen ablehnen und/oder eine Beendigung ihrer Nachbefragung wünschen.

Mögliche Unannehmlichkeiten und Risiken
Für Sie bedeutet die Studie eine geringe zeitliche Mehrbelastung (Zeitangaben s. o.). Risiken ergeben sich für Sie keine.

Umstände, die zum Abbruch der Studienteilnahme führen
Widerruf der Teilnahme durch den Studienteilnehmer bzw. die Studienteilnehmerin.

Nutzen
Der Nutzen besteht für Sie in einer zum einen in einer grundlegenden Information über Ihr Risikoprofil. Weiterhin erhalten Sie zusätzliche grundlegende Informationen über den Verlauf Ihres Krankenhausaufenthaltes und die Möglichkeiten der Unterstützung nach der Krankenhausentlassung. Es wird Ihnen ermöglicht, aktiv an Ihrer Therapie mitzuwirken.
Sie können jederzeit mit Studienmitarbeitern Fragen zur Teilnahme und zu Ergebnissen Ihrer Untersuchungen klären. Sie können sich auch zu jedem späteren Zeitpunkt gerne mit Mitarbeitern der Studie in Verbindung setzen, wenn Sie eine weiter gehende Beratung bezüglich der bei dem Führen des Heftes auftretenden Probleme haben wollen oder eine weitergehende Beratung bezüglich Ihrer Lebensstilrisiken wünschen.

Alternativen zur Teilnahme

Wenn Sie sich nicht zur Teilnahme entschließen können, erhalten Sie in jedem Fall eine Behandlung, die dem aktuellen Wissensstand entspricht.

Pflichten des Teilnehmers:
Die Pflichten des Teilnehmers während der Studienphase liegen in der nach Möglichkeit vollständigen und wahrheitsgemäßen Beantwortung bei den Befragungen und bei der Protokollierung der einzelnen Prozessschritte in dem Patiententagebuch.

Neue Erkenntnisse
Sollten im Verlauf der Studie neue wissenschaftliche Erkenntnisse bekannt werden, die eine bessere Therapie/Behandlung bzw. eine Änderung in der Therapie/Behandlung nach sich ziehen, werden Sie über diesen Sachverhalt zeitnah informiert.

Datenschutz

Die Studie wird nach den geltenden datenschutzrechtlichen Bestimmungen durchgeführt.
Ihre sämtlichen personenbezogenen, während der Studie erhobenen Daten werden vor ihrer Übermittlung pseudonymisiert. Damist sind Ihre Daten für den Empfänger anonym, d.h. er kann keine Verbindung zwischen Ihren Daten und Ihrer Person herstellen.
Durch Ihre Unterschrift auf der Einwilligungserklärung erklären Sie sich damit einverstanden, dass die Studienärzte OÄ Dr. M. Schmidt und Dr. P. Breuer und ihre wissenschaftlichen Mitarbeiter Ihre personenbezogenen Daten (Name, Anschrift, Geschlecht, Geburtsdatum, Telefonnummern, Daten zu ihrer physischen und psychischen Gesundheit oder andere Daten, die während ihrer Teilnahme an der Studie oder bei Folgeuntersuchungen erfasst wurden) für Zwecke der o.g. Studie erheben und verarbeiten sowie für die Verwaltung und Durchführung der Studie sowie für Zwecke der Forschung und statistischen Auswertung verwenden dürfen.
Weiterhin erklären Sie sich damit einverstanden, dass im Rahmen der Routinediagnostik ermittelte Parameter im Blut sowie klinische Diagnosen vom Studienarzt Ihrer Krankenakte entnommen werden. Hierfür (und nur für diese Angaben) entbinden Sie Ihren behandelnden Arzt von seiner ärztlichen Schweigepflicht.

Der Studienleiter gibt während der Studie erhobene studienbezogene Daten an von ihm beauftragte Dienstleister (Prof. Wernecke, Biometrie) in pseudonymisierrter Form weitergeben, die Ihre studienbezogenen Daten ausschließlich für den Zweck (Auswertung) der o.g. Studie verwendet.
Die an den vorgenannten Empfänger weitergebenen studienbezogenen Daten enthalten nicht Ihren Namen oder Ihre Adresse. Stattdessen versieht der Studienarzt die Studiendaten mit einer Codenummer (Pseudonymisierung der Daten). Auf den Codeschlüssel, der es erlaubt, die studienbezogenen Daten mit Ihnen in Verbindung zu bringen, haben nur der Studienarzt und seine Mitarbeiter Zugriff.
Ihre Einwilligungserklärung wird getrennt vom anderen Datensatz so aufbewahrt, dass nur die wissenschaftlichen Mitarbeiter darauf zugreifen und eine personenbezogene Zuordnung vornehmen können.

Alle Ihre persönlichen Daten, die sich beim Studienarzt befinden, können vom Leiter der Studie und/oder seinen Vertretern und Auftragsunternehmen (z.B. Monitore oder

Auditoren), sowie der unabhängigen Ethikkommission eingesehen werden. Zweck dieser Prüfungen ist, sicherzustellen, dass die Studie ordnungsgemäß durchgeführt wird und die Qualität Ihrer studienbezogenen Daten gewährleistet ist.
Sie können jederzeit der Weiterverarbeitung Ihrer im Rahmen der o.g. Studie erhobenen Daten und/oder weiteren Untersuchung der Ihnen entnommen Probe widersprechen und ihre Vernichtung bzw. Löschung verlangen.

Die erhobenen Daten werden nach Abschluss der Studie nach Ablauf von 10 Jahren gelöscht, sofern nicht gesetzliche, satzungsmäßige oder vertragliche Aufbewahrungsfristen entgegenstehen. Die Ihnen entnommenen Routine-Blutproben werden an das Labor der Charité – Universitätsmedizin Berlin zur dortigen Untersuchung übermittelt. Die Proben werden über einen Zeitraum von höchstens einem Monat im Labor gelagert und danach vernichtet.

Sie haben das Recht auf Auskunft über alle beim Studienarzt vorhandenen personenbezogenen Daten über Sie. Sie haben auch ein Anrecht auf Korrektur eventueller Ungenauigkeiten in Ihren personenbezogenen Daten. Wenn Sie eine Anfrage machen wollen, wenden Sie sich bitte an Ihren Studienarzt. Dieser wird Kontakt mit dem Leiter der Studie aufnehmen und Ihnen die Information über Ihre gespeicherten Daten zur Verfügung stellen.
Bitte beachten Sie, dass die Ergebnisse der Studie in der medizinischen Fachliteratur veröffentlicht werden können, wobei die Daten so aufbereitet werden, dass keine Rückschlüsse auf personenbezogene Angaben bestehen. Die Verarbeitung der studienbezogenen Daten liegt beim Studienleiter.

Versicherungsschutz
Es wurde keine spezielle Patientenversicherung für diese Studie abgeschlossen.
Der Studienärzte und –mitarbeiter sind jedoch durch die Betriebshaftpflichtversicherung der Charité gegen Haftungsansprüche, welche aus ihrem schuldhaften Verhalten resultieren könnten, versichert.
Sollten Sie möglicherweise aufgrund schuldhaften Handelns eines Charité–Mitarbeiters einen Schaden erleiden, wenden Sie sich bitte unverzüglich an die Studienleiterin.

Freiwilligkeit der Teilnahme
Ihre Teilnahme an der Untersuchung ist absolut freiwillig. Sie hat keinen Einfluss auf den Verlauf Ihrer Behandlung. Sie haben natürlich jederzeit die Möglichkeit, Ihr Einverständnis zu dieser Untersuchung zu widerrufen, ohne dass Ihnen Nachteile entstehen.

Kosten
Durch die Teilnahme an der Studie werden Ihnen keine weiteren Kosten entstehen.

Bezahlung
Eine Bezahlung der Studienteilnehmer ist nicht geplant.

Fragerecht / Mitteilungspflicht

Sie haben jederzeit die Möglichkeit, den Studienärzten weitergehende Fragen über alle Angelegenheiten zu stellen, welche die Studie betreffen, insbesondere auch über Risiken.

Fragen können gerichtet werden an:

Dr. P. Breuer Tel.: 030-450 631093
Prof. Dr. med. C. Spies, OÄ Dr. M. Schmidt Tel.: 030-450-551001 und - 531012
Per E-Mail an: philipp.breuer@charite.de oder maren.schmidt@charite.de
Auf dem Postwege an die oben stehende Anschrift.

Herzlichen Dank für Ihre Teilnahme,
alles Gute wünscht Ihnen

____
Prof. Dr. med. Claudia D. Spies
Klinikdirektorin

CHARITÉ

Anlage 3 Einverständniserklärung

CharitéCentrum für Anästhesiologie, OP-Management und Intensivmedizin

Charité - Universitätsmedizin Berlin | D - 13344 Berli

Einwilligungserklärung

Version 1.0 vom 25.11.2008

zur Teilnahme an der Studie

„Patientenmitbestimmung und Risiko-gestützte Behandlung zur Verbesserung des Outcomes bei älteren Patienten nach gastrointestinaler, thorakaler und urogenitaler Onkochirurgie“

Kurztitel: PERATECS

Studienleiter: Univ.-Prof. Dr. C. Spies

Universitätsklinik für Anästhesiologie mit Schwerpunkt operative Intensivmedizin CCM / CVK

Klinikdirektorin:
Univ.-Prof. Dr. C. Spies

Zertifiziert nach DIN EN ISO 9001

Campus Virchow-Klinikum
Augustenburger Platz 1
13353 Berlin
Tel: +49 30 450 551-001/002/022
Fax: +49 30 450 551909
anaesthesie-virchow-klinikum@charite.de

Campus Charité Mitte
Charitéplatz 1
10117 Berlin
Tel: +49 30 450 531-012/52
Fax: +49 30 450 531911
anaesth@charite.de

http://www.charite.de/ch/anaest/

Bitte lesen Sie die Patienteninformation und die Einwilligungserklärung sorgfältig durch.
Bitte fragen Sie bei allen Unklarheiten oder wenn Sie weitere Informationen wünschen.

Patient:________________________________ Patienten - Pseudonym.: ____________________

(Name, Vorname)

Ich willige in die Teilnahme an der Studie ein.

Ich bin durch den Studienarzt anhand der Patienteninformation Version 1.0 vom 25.11.2008

- mündlich und schriftlich über Ziel, Dauer, Ablauf, Rechte und Pflichten, sowie Nutzen, Risiken und Nebenwirkungen der klinischen Studie aufgeklärt worden. Ich hatte ausreichend Gelegenheit Fragen zu stellen. Diese wurden mir vom aufklärenden Studienarzt verständlich beantwortet. Außerdem habe ich Kopien der schriftlichen Patienteninformation und der Patienteneinwilligung erhalten. Ich hatte genügend Zeit, um meine Entscheidung zur Teilnahme an dieser klinischen Studie frei zu treffen.

- Ich weiß, dass meine Teilnahme an der Studie völlig freiwillig ist und dass ich diese Einwilligung jederzeit und ohne Angabe von Gründen widerrufen kann, ohne dass mir daraus Nachteile für meine weitere Behandlung entstehen.

- Im Rahmen dieser klinischen Studie sind die Studienärzte durch die Betriebshaftpflichtversicherung der Charité gegen Haftungsansprüche, welche aus ihrem schuldhaften Verhalten resultieren könnte, versichert. Die Meldung der Studie gegenüber der Betriebshaftpflichtversicherung der Charité ist erfolgt. Die

Versicherungsbedingungen wurden mir vom Studienarzt erklärt und ich habe sie verstanden.

Datenschutzerklärung

Einwilligungserklärung zur Datenverarbeitung

Ich erkläre mich damit einverstanden, dass im Rahmen dieser Studie erhobene Daten über meine Gesundheit, in Papierform und auf elektronischen Datenträgern in der Klinik für Anästhesiologie und operative Intensivmedizin, Charité, Campus Virchow Klinikum, Berlin, in der in der Patienteninformation beschriebenen Form, aufgezeichnet und weitergegeben werden dürfen.
Weiterhin erklären ich mich damit einverstanden, dass für die Routine-Laborwerte, sowie klinische Diagnosen vom Studienarzt der Krankenakte entnommen werden. Hierfür (und nur für diese Angaben) entbinde ich meinen behandelnden Arzt von seiner ärztlichen Schweigepflicht.

Ich bin damit einverstanden, dass mich die Studienärzte schriftlich oder telefonisch unter Wahrung des Datenschutzes in den nächsten 12 Monaten mehrmals kontaktieren (nach 3 und 12 Monaten).

Ich bin mit einer schriftlichen ☐ und / oder telefonischen ☐ Kontaktaufnahme einverstanden:

Straße ____ Nr. ___________

PLZ __________ Ort ____

Telefon: __________________________ Mobiltelefon:________________________

Am besten tel. erreichbar: ❑ vormittags ❑ nachmittags ❑ abends ❑ sonstiges__________

Ich bin vom Studienarzt mündlich aufgeklärt worden und habe die schriftliche Patienteninformation gelesen und verstanden. Ich willige in die Teilnahme an der Studie ein.

_________________ ____

Ort, Datum Name (leserlich) und Unterschrift des Patienten

_________________ ____

Der Patient wurde von mir über Ziel, Dauer, Ablauf, Nutzen, Risiken und Nebenwirkungen der klinischen Prüfung mündlich und schriftlich aufgeklärt. Aufgetretene Fragen wurden von mir verständlich und ausreichend beantwortet. Der Patient hat ohne Zwang seine Einwilligung erteilt. Eine Kopie der schriftlichen Patienteninformation und dieser Patienteneinwilligung habe ich dem Patienten ausgehändigt.

Ort, Datum	Name (leserlich) und Unterschrift des Studienarztes

Anlage 4 Stellungsnahme zur Betriebshaftpflicht
Anlage 5 Votum des Datenschutzbeauftragten
