# Supplementary material for: Patient Empowerment Improved Perioperative Quality of Care in Cancer Patients Aged ≥ 65 Years – A Randomized Controlled Trial
Source: PLoS One. 2015 Sep 17;10(9):e0137824. doi: 10.1371/journal.pone.0137824 (PMC4574984; doi:10.1371/journal.pone.0137824)
Supplement: S2 Protocol — (PDF) [file pone.0137824.s004.pdf]

Antrag auf Beratung durch die Ethikkommission zur Durchführung eines medizinisch-wissenschaftlichen Vorhabens, welches nicht die klinische Prüfung eines Arzneimittels beinhaltet

1. Titel der Studie	Patienten Empowerment und risiko-adaptierte Behandlung zur Verbesserung des Outcomes älterer Patienten nach gastrointestinalen, thorakalen und urogenitalen Operationen bei malignen Erkrankungen (PERATECS)<br>Prospektive, randomisierte, kontrollierte Studie
2. Ethikkommissions -Antragsnummer	(wird von der Ethikkommission vergeben)
3. Entscheidungen anderer Ethikkommissionen in derselben Sache	Nicht vorhanden
4. Gegenstand der Studie und ihre Ziele (Hypothesen, getrennt in Haupt- und Sekun-därhypothesen)	Die Ziele dieser randomisiert, kontrollierten Studie sind, physische, psychologische und sozial benachteiligende postoperative Limitationen durch aktive Involvierung des Patienten (Patienten-Empowerment) zu vermeiden und damit den Rehabilitationsprozess zu beschleunigen sowie eine postoperativ verbesserte Lebensqualität für den älteren Patienten zu erreichen.<br><br>**<u>Haupthypothesen</u>**<br>Die Haupthypothesen dieser Studie sind, dass ein risiko-adaptiertes Patienten-Empowerment im Rahmen evidenzbasierter Medizin<br>(a) die Zeit bis zur Erfüllung der Krankenhausentlassungskriterien reduziert, sowie<br>(b) die Lebensqualität bei älteren Patienten im Vergleich zu Kontrollpatienten mit Standardtherapie verbessert.<br><br>**<u>Nebenhypothesen</u>**<br>Ein risiko-adaptiertes Patienten-Empowerment im Rahmen evidenzbasierter Medizin führt zu<br>(c) einer Reduktion der kurzfristigen postoperativen Komplikationen,<br>(d) einer reduzierten Wiederaufnahmerate,<br>(e) einer StressReduktion, d.h. weniger postoperative Schmerzen, schnellere Mobilisation, weniger postoperative Übelkeit und Erbrechen,<br>(f) einer reduzierten Mortalitätsrate ein Jahr nach Entlassung,<br>(g) einer Verbesserung der Geriatrischen Depressions Skala bei älteren Patienten im Vergleich zu Kontrollpatienten mit Standardtherapie.<br><br>Darüber hinaus soll aus dieser Studie das Risikoprofil älterer Patienten in Bezug auf mögliche postoperative Komplikationen mittels logistischer Regression (multivariat) ermittelt werden. Als Einflussgröße der Regression soll, gemeinsam mit anderen wichtigen klinischen Parametern, das risiko-adaptierte Patienten-Empowerment untersucht werden.<br><br>**Primärer (multipler) Endpunkt:**<br>1. Zeit bis zur Entlassung<br>2. Lebensqualität<br><br>**Sekundäre Endpunkte:**<br>1. Postoperative Komplikationen<br>2. Wiederaufnahmerate<br>3. Stress durch postoperative Schmerzen, mangelnde Mobilisation, Übelkeit oder Erbrechen<br>4. Mortalität nach einem Jahr<br>5. Depression, gemessen anhand der geriatrischen Depressionsskala

5. Erläuterung der Bedeutung der Studie	**Medizinischer Hintergrund**<br>Ältere Menschen sind die am schnellsten wachsende Bevölkerungsgruppe in der westlichen Welt [1]. Zwei Drittel aller soliden Tumoren treten bei Patienten ab dem 65. Lebensjahr auf [2]. Die Carcinom-Inzidenz ist bei Patienten über 65 Jahren 11mal höher als bei jüngeren Patienten [2]. Trotz Weiterentwicklung chirurgischer Techniken blieb die perioperative Morbidität in den letzten Jahren unverändert hoch [3]. Außerdem erhalten immer noch deutlich weniger ältere Patienten beispielsweise eine elektive Resektion bei kolorektalem Carcinom als die entsprechenden jüngeren Patienten [4, 5], obwohl das Outcome der verschiedenen Altersgruppen bei sorgfältiger Indikationsstellung und Abwägung der Risikofaktoren ähnlich ist. Dabei führt eine inadäquate Therapie gerade bei älteren Krebspatienten zu einer erhöhten Mortalität sowie postoperativer eingeschränkter Lebensqualität [6-9].<br>Die Gründe für die erhöhte postoperative Morbidität nach größeren chirurgischen Eingriffen sind unterschiedlich. Die chirurgische Stressantwort ist im Alter verändert mit einem Anstieg postoperativer Komplikationen [10] resultierend in einer verlängerten Krankenhausverweildauer und einer schlechteren Konvaleszenz [11-15].<br><br>Erst in den beiden letzten Jahrzehnten wurde die prognostische Bedeutung der erhöhten Komorbidität bei älteren Patienten erkannt und Risikofaktoren als prognostische Indikatoren realisiert [16]. Dennoch ist das präoperative Risikoprofil für diese Altersgruppe nur unzureichend validiert [17]. Dies führt einerseits zu einem Ausschluss relativ fitter älterer Patienten von erfolgsversprechenden Therapien andererseits aber auch zu schmerzvollen und nicht erfolgreichen Operationen bei Patienten mit einem hohen perioperativen Risiko [18].<br><br>**Patienten Empowerment**<br>Innerhalb der letzten 40 Jahre hat sich das Bild des Patienten grundlegend geändert. Während früher der Patient eine passive Rolle spielte, übernehmen die heutigen Patienten mehr und mehr Entscheidungen und fordern Autonomie ein. Die Einbindung gut informierter Patienten in Therapieentscheidungen führte in Studien zu einer deutlich verbesserten Compliance von Seiten der Patienten und einer höheren Zufriedenheit für die Patienten als auch die behandelnden Ärzte [19].<br>Die WHO definiert Empowerment als "Grundvoraussetzung für Gesundheit" („prerequisite for health") [20]. Empowerment zielt darauf ab, die Bedürfnisse von Betroffenen als zentrales Kriterium bei der Planung und Bereitstellung spezifischer Behandlungs- und Versorgungsangebote zu berücksichtigen.<br>Maßnahmen zum Empowerment von Patienten beinhalten Interventionen für verbesserte Informations- und Entscheidungshilfen angepasst an die speziellen Bedürfnisse der älteren Patienten, intra- and postoperative Stressprävention, sowie frühzeitige Rehabilitation und Kontrolle eines riskanten Lebensstils in einer multimodalen Vorgehensweise [11-15]. Derartige evidenzbasierte Interventionen können das postoperative Outcome sowie die postoperative Lebensqualität verbessern [21].<br><br>Diese Maßnahmen sind bei Erkrankungen wie Diabetes mellitus sowie anderen schweren chronischen Erkrankungen wie COPD, terminalem Nierenversagen und auch Krebserkrankungen erfolgreich durchgeführt worden [22-24]. Allerdings sind ältere Krebspatienten deutlich unterrepräsentiert [25]<br><br>**Information und soziale Unterstützung**<br>Dem Wunsch der (älteren) Patienten nach Informationen und partizipierter Entscheidungsfindung wird häufig nicht entsprochen [26]. Die perioperative Informationsübermittlung und –aufbereitung sollte speziell für ältere Menschen verbessert werden, um diese aktiv in den Heilungsprozess mit einzubinden. Eine dem Patienten angepasste Informationsweitergabe prä – und postoperativ verbessert die Compliance der Patienten während der Therapie [19, 27].<br><br>Patienten mit einer Krebs-Erkrankung mit einer guten sozialen Einbindung und Unterstützung leiden deutlich weniger unter schweren Stress-Reaktionen als Patienten

mit einem geringen sozialen Support [28]. Das Risiko von Wiederaufnahmen ins Krankenhaus ist bei Patienten ohne soziale Unterstützung ebenso erhöht wie das Risiko eines verschlechterten Outcomes [28]. Soziale Unterstützung und Einbindung können beispielsweise durch frühe Einbindung in bzw. Unterstützung durch Selbsthilfegruppen geleistet werden.

Lebensqualität (Quality of life QoL)

Fortschritte in den chirurgischen, anästhesiologischen und intensivmedizinischen Techniken erlauben die verbesserte Behandlung älterer Menschen. Über deren postoperative Lebensqualität (QoL) ist jedoch nur wenig bekannt.

Chirurgische Eingriffe haben einen unmittelbaren Einfluss auf verschiedene Bereiche der Lebensqualität: Patienten leiden unter Schmerzen, Übelkeit und Erbrechen. Die physikalische Leistungsfähigkeit kann eingeschränkt sein. Je nach Art und Ausmaß der Operation kann es zu langfristigen Veränderungen bzw. Einschränkungen kommen. So hat beispielsweise ein nach rektaler Chirurgie angelegtes Stoma Einflüsse auf die urogenitale und gastrointestinale Funktion des betroffenen Patienten.

Bei Patienten mit fortgeschrittenen Malignomen waren die globale Quality of Life (QoL) und die soziale Funktionen kontrolliert nach Alter, Leistung und Metastasenstatus prognostisch für das Überleben [29-34]. Ein präoperativ reduzierter QoL oder eine stark eingeschränkte funktionelle Leistungsfähigkeit korrelierten mit einer verringerten Lebensqualität nach Entlassung [35]. Zusätzlich entwickeln bis zu 30% der älteren Patienten Depressionen und Angst [36]

Verschiedene psychologische Schulungen können ebenso zu einer verbesserten Lebensqualität führen wie Übungen zur Stressreduktion [22, 37-39] [40, 41]. Studien mit sozialer Unterstützung durch Selbsthilfegruppen führten ebenfalls zu einer verbesserten Lebensqualität [42-44]. Allerdings sind diese Studien vor allem bei an brustkrebserkrankten Patientinnen im mittleren Lebensalter durchgeführt worden [39].

Methode:

Alle Patienten, die die Einschlusskriterien erfüllen und 65 Jahre alt oder älter sind, werden konsekutiv gescreent, wenn sie sich einer Operation aufgrund eines gastrointestinalen, urogenitalen oder pulmonalen Carcinoms unterziehen müssen,

Falls die Patienten in die Studie eingeschlossen werden können, werden sie randomisiert kontrolliert zwei Behandlungsarmen zugeordnet: Patienten-Empowerment oder Standardbehandlung. (s. Anhang Nr 1 Flow Chart)

Sie erhalten präoperativ verschiedene Fragebögen mit Fragen zu Komorbiditäten, Leistungsfähigkeit, Ernährungsstatus, Depression und Lebensqualität.

Postoperativ werden die Patienten bis einschließlich des fünften postoperativen Tages täglich besucht, um Schmerzen, Mobilisation, Ernährung und ggf. Delir zu dokumentieren. Am Entlassungstag werden die Patienten noch einmal besucht.

Die Intervention zum Patienten–Empowerment wird folgendermaßen aussehen: Diese Patienten erhalten vermehrte Information in Form eines Patiententagebuches sowie präoperative und postoperative Informationen zu sozialer Unterstützung. Es ist geplant, dass Selbsthilfegruppen die Patienten prä- und postoperativ aufsuchen und informieren bzw. unterstützen.

Drei Monate und ein Jahr nach der Krankenhausentlassung werden die Patienten erneut kontaktiert. Es werden Fragen zur Lebensqualität und Depression sowie erneuten Krankenhausaufenthalten, Komplikationen und neu aufgetretenen Erkrankungen gestellt.

Patientenanzahl:

Die Studie wird in der Charité- Universitätsmedizin Berlin, dem Klinikum der Universität München sowie dem Universitätsklinikum Heidelberg/Mannheim durchgeführt.

Leiterin der Studie ist Univ.-Prof. Dr. C. Spies, Universitätsklinik für Anästhesiologie mit Schwerpunkt operative Intensivmedizin, Charité – Universitätsmedizin Berlin, CCM/CVK.

Nach biometrischer Kalkulation sollen pro Zentrum circa 230 Patienten, insgesamt also 690 Patienten eingeschlossen werden.

	**Studiendauer:**<br>Insgesamt 2 Jahre, Studiendauer pro Patient: 1 Jahr
6. Welche der folgenden Bestimmungen finden Anwendung<br>a) Medizinproduktegesetz<br>b) Strahlenschutzverordnung<br>c) Röntgenverordnung<br>d) Gentechnikgesetz<br>e) Datenschutzgesetze	e) Datenschutzgesetz
7. Ggf.: Bezeichnung und Charakterisierung der Prüfprodukte	Entfällt
8. wesentliche Ergebnisse der vorklinischen Tests oder Gründe für die Nichtdurchführung derselben	Nicht anwendbar
9. Wesentlicher Inhalt und Ergebnisse der vorangegangenen Studien/Anwendungen der in der Studie zu prüfenden Produkte	In vorhergehenden Studien konnte der positive Einfluss von Patienten-Empowerment durch soziale und informative Unterstützung bei Erkrankungen wie Diabetes mellitus sowie anderen schweren chronischen Erkrankungen wie COPD, terminalem Nierenversagen und auch Krebserkrankungen gezeigt werden. In den bisherigen Studien sind aber die Patienten mit einem Alter über 65 Jahren unterrepräsentiert und Untersuchungen zur partizipativen Entscheidungsfindung nicht ausreichend untersucht.<br><br>Ebenso wurden bisher bei dieser Patientengruppe ≥ 65 Jahre keine spezifischen Risikoerhebungen durchgeführt. Es wurden jedoch durch unsere Klinik einige von der Ethikkommission genehmigte Studien in der Anästhesieambulanz durchgeführt, z.B.:<br>• „Prävention Nikotin-assoziierter Erkrankungen durch Raucherentwöhnung“ [45, 46]<br>• “Bedeutung von Lebensstil als Risiko für operative Eingriffe”[47-49]<br>• „PART (Patient Active Role Training)”
10. Beschreibung der vorgesehenen Maß-nahmen/Untersuchungsmethoden und eventuelle Abweichungen von den in der med. Praxis üblichen Maßnah-	Die perioperative Behandlung erfolgt für alle Patienten nach den publizierten und zertifizierten (nach DIN EN ISO 9001) Standards der beteiligten Kliniken. Es erfolgt keine studienbedingte Anpassung der Maßnahmen oder der Untersuchungen inklusive Routine-Blutentnahmen. Das beinhaltet die Narkosevorbereitung, die Induktion und Aufrechterhaltung sowie die postoperative Überwachung im Aufwachraum bzw. auf den Intensivpflegestationen und das indizierte invasive Monitoring der Patienten. Die Untersuchungen des Blutes des Patienten entsprechen ebenfalls der Routine-Diagnostik der beteiligten Kliniken. Die Entlassung aus dem Aufwachraum bzw. den Intensivpflegestationen wird aufgrund der Erfüllung der Entlassungskriterien nach dem Aldrete Score festgelegt.

men/Untersuchungen	Für die Durchführung der Studie werden von den Patienten zusätzlich Fragebögen ausgefüllt:<br>Das präoperative und postoperative Screening in der Kontrollgruppe sowie in der Interventionsgruppe beinhaltet das Ausfüllen von mehreren Fragebögen: präoperativ werden diverse Fragebögen hinsichtlich körperlicher Leistungsfähigkeit, Komorbiditäten, Ernährungsstatus, Lebensqualität, Depression und Lebensstilrisiken (Nikotin, Alkoholkrankheit) ausgefüllt. Der gesamte Zeitaufwand beträgt höchstens 40 Minuten. Postoperativ werden die Patienten bis einschließlich des fünften postoperativen Tages täglich besucht, um Schmerzen, Mobilisation, Ernährung und ggf. Delir zu dokumentieren. Diese Besuche nehmen ca. 15 Minuten in Anspruch. Am Entlassungstag werden die Patienten noch einmal besucht (ca. 15 Min).<br>Drei und zwölf Monate später werden die Patienten noch einmal zu Lebensqualität und Depression befragt (ca. 20 Minuten). Die Patienten in der Interventionsgruppe werden sowohl zusätzlich präoperativ als auch postoperativ von geschulten Mitgliedern der entsprechenden Selbsthilfegruppen aufgesucht und informiert. Außerdem erhalten diese Patienten eine Art Patiententagebuch. Das Ausfüllen des Tagebuches sollte nicht mehr als 15 Minuten täglich in Anspruch nehmen.
11. Bewertung und Abwägung der vorhersehbaren Risiken und Nachteile der Studienteilnahme gegenüber dem erwarteten Nutzen für die Studienteilnehmer und zukünftig erkrankte Personen	Patientensicherheit wird gewährleistet durch die behandelnden Ärzte der beteiligten Kliniken im Rahmen der klinischen Versorgung entsprechend den gültigen Standard Operating Procedures (SOPs), durch die ein hohes Maß an evidenz-basierten Medizin umgesetzt wird.
a. Voraussehbarer therapeutischer Nutzen für die Studienteilnehmer	Der voraussehbare Nutzen der Studienteilnehmer der Interventionsgruppe besteht zum einen in einer präoperativen Risikoanalyse und eines Risikomanagements durch aktive Involvierung des Patienten (Patient Empowerment*)* zur eventuellen Erzielung einer Reduktion kurzfristiger Komplikationen und Stressprävention. Dieser therapeutische Nutzen ist zwar nicht ausreichend an Patienten mit einem Alter über 65 Jahren gezeigt, es ist aber zu erwarten, dass entsprechend den bisherigen Studien auch in dieser Studie dieser Nutzen nachzuweisen ist.<br>Die Studienteilnehmer ohne Intervention erhalten im Verhältnis zu einer Standardversorgung keine Nachteile und werden entsprechend den Standards der beteiligten Kliniken behandelt, ohne darüber hinaus einen zusätzlichen Nutzen zu haben.<br>-
b. Voraussehbarer medizinischer Nutzen für zukünftig erkrankte Personen	Die Implementierung von informativer und sozialer Unterstützung zur partizipativen Entscheidungsfindung in multimodalen, interdisziplinären Behandlungsmodulen wäre auch in Zukunft von Profit für ältere Patienten mit Tumorerkrankungen.
c. Risiken und Belastungen für die Studienteilnehmer (alle im Einzelnen auflisten)	Das präoperative und postoperative Screening in der Kontrollgruppe beinhaltet das Ausfüllen von mehreren Fragebögen: präoperativ werden diverse Fragebögen hinsichtlich körperlicher Leistungsfähigkeit, Komorbiditäten, Ernährungsstatus, Lebensqualität, Depression und Lebensstilrisiken (Nikotin, Alkoholkrankheit) ausgefüllt. Der gesamte Zeitaufwand beträgt höchstens 40 Minuten. Postoperativ werden die Patienten bis einschließlich des fünften postoperativen Tages täglich besucht, um Schmerzen, Mobilisation, Ernährung und ggf. Delir zu dokumentieren. Diese Besuche nehmen ca. 15 Minuten in Anspruch. Am Entlassungstag werden die Patienten noch einmal besucht (ca. 15 Min).<br>Drei und zwölf Monate später werden die Patienten noch einmal zu Lebensqualität und Depression befragt (ca. 20 Minuten).<br>Die Patienten in der Interventionsgruppe werden sowohl präoperativ als auch postoperativ

	von geschulten Mitgliedern der entsprechenden Selbsthilfegruppen aufgesucht und informiert. Außerdem erhalten diese Patienten eine Art Patiententagebuch. Das Ausfüllen des Tagebuches sollte nicht mehr als 15 Minuten täglich in Anspruch nehmen.<br>Risiken ergeben sich unseres Erachtens aus der Studie für die Patienten nicht.
12. Maßnahmen zur Risikobeherrschung	Die Patienten werden nach Studienaufnahme bis zu Ihrer Entlassung durch die behandelnden Ärzte der beteiligten Kliniken überwacht und durch das Studienteam täglich bis zum einschließlich fünften Tag nach der Operation und am Tag vor der Entlassung visitiert.
13. Abbruchkriterien	Unter den nachfolgenden Bedingungen tritt ein vorzeitiges Ausscheiden eines Patienten aus der Studie gemäß der Abbruchkriterien ein:<br>Die folgenden Ereignisse können als Begründung angesehen werden:<br>• persönlicher Wunsch des Patienten<br>• jede andere Situation, bei der nach Ansicht des Studienarztes eine weitere Teilnahme an der klinischen Prüfung nicht im besten Interesse des Patienten sein würde<br>• signifikante Protokollverletzungen<br>• Nachträgliches Auftreten eines Ausschlusskriteriums<br><br>Ein vorzeitiges Ende der Studie bzw. ein Abbruch der gesamten Studie kann aufgrund folgender Umstände in Frage kommen:<br>• Entscheidung des Studienleiters bei unvertretbaren Risiken unter Nutzen-Risiko-Abwägung<br>• neue (wissenschaftliche) Erkenntnisse während der Laufzeit der klinischen Prüfung, die die Sicherheit der Studienteilnehmer gefährden können (positive Nutzen-Risiko-Abwägung nicht mehr gegeben)
14. Anzahl, Alter und Geschlecht der betroffenen Personen	Die Studie wird parallel an 3 großen Zentren (Berlin, München, Heidelberg) durchgeführt unter Leitung von Frau Prof. Dr. C. Spies, Klinik für Anästhesiologie und operative Intensivmedizin, Charité, Universitätsmedizin – Berlin.<br>Es ist geplant, dass pro Zentrum circa 230 Patienten, insgesamt also 690 Patienten eingeschlossen werden. Mit einem ca. 30%igen Verlust während des Studienzeitraums wäre mit insgesamt 480 Patienten, dementsprechend 240 Patienten pro Gruppe zu rechnen.<br>Die teilnehmenden Patienten sind beiderlei Geschlechts und älter als 65 Jahre.
15. Statistische Planung und Angabe sowie biometrische Begründung der Fallzahl und Unterschrift des/der Statistikers/Statistikerin	Exploratorische Datenanalyse der primären und sekundären Endpunkte: Häufigkeitsverteilungen, Mediane, 25% -75% Perzentile, arithmetische Mittelwerte, Standardabweichungen und zugehörige 95%-Konfidenzintervalle.<br>Wilcoxon (Mann-Whitney) U-Test bzw. Mantel-Haenszel-Test für die beiden primären Endpunkte (Fehler 1. Art Bonferroni-adjustiert). Lebensqualität: Mantel-Haenszel-Tests und Mann-Whitney U-Tests.<br>Für die sekundären Endpunkte: Kaplan-Meier Schätzungen der Überlebenskurven, Log-Rank-Test auf Unterschiede zwischen Intervention und Kontrolle, Chi-Quadrat-Tests für Kontingenztafeln. Zusätzliche logistische Regressionen, um ein multivariates Risikoprofil älterer Patienten in Bezug auf postoperative Komplikationen zu erstellen.<br>Statistische Tests für sekundäre Endpunkte verstehen sich lediglich als exploratorisch, dafür werden keine alpha-Adjustierungen vorgenommen.<br><br>**Fallzahlberechnung**<br>Worst-case-Szenario in Bezug auf den zweiten primären Endpunkt:<br>Erhöhung des globalen Wertes der Lebensqualität im EORTCQLQ C-30 von 68,82 (±15,51) auf 73,12 (±13,73) nach 12 Monaten.<br>α = 0.025 (Bonferroni-adjustiert), Power 80%.<br>Verwendeter Test: nichtparametrischer Wilcoxon (Mann-Whitney) –Rangsummentest<br>Ergebnis: Es werden 235 Patienten pro Gruppe benötigt.<br>Bezugnahme auf den ersten primären Endpunkt:<br>Endpunkt: 12 versus 10 Tage Krankenhausaufenthalt bei Effektgröße von 0,5 und einer

	Power von 80% (Fehler 1. Art Bonferroni-adjustiert auf α = 0,025):<br>Verwendeter Test: nichtparametrischer Wilcoxon (Mann-Whitney) –Rangsummentest<br>Ergebnis: 83 Patienten pro Gruppe werden benötigt<br><br>**Charité**<br>Präoperative Anästhesieambulanz: Patienten / Jahr = 40.000<br>Patienten mit einer geplanten onkochirurgischen Operation n = 776<br>Patienten, die in die Studie eingeschlossen werden n = 230 (115 pro Gruppe)<br><br>Kooperierende Zentren: 230 Patienten/Jahr werden eingeschlossen.<br><br>Bei drei teilnehmenden Zentren sollten also insgesamt 690 Patienten (230 pro Zentrum) eingeschlossen werden. Bei einem kalkuliertem Verlust von 30% der Patienten während des Beobachtungszeitraumes, gehen wir von **n = 480** Patienten nach 2 Jahren aus, d.h. **n1 = n2 = 240** Patienten pro Gruppe.<br><br>Kalkulation mittels nQuery Advisor® Release 7.0, Stat. Solutions Ltd. & South Bank, Crosse's Green, Cork, Irland.<br><br>Durchgeführt von Prof. Wernecke Juli 2008
16.<br>a. Darlegung und ggf. Erläuterung der **Ein- und Ausschlusskriterien**	**Einschlusskriterien**<br>• Durchgeführte Patientenaufklärung und schriftliche Einwilligung<br>• Patienten ≥ 65 Jahre, die sich aufgrund einer malignen Erkrankung einer Operation unterziehen werden (gastrointestinale, pulmonale oder urogenitale Lokalisation des Tumors)<br>• Mini Mental State > 23<br>• Lebenserwartung > 2 Monate<br>• Keine Teilnahme an anderen Studien während der Studiendauer<br><br>**Ausschlußkriterien:**<br>• Fehlende schriftliche Einwilligungserklärung<br>• Fehlende Einwilligungsfähigkeit<br>• Notfalleingriffe<br>• Ambulante Patienten<br>• Alter < 65 Jahre<br>• Teilnahme an einer anderen klinischen Studie innerhalb der letzten Woche vor dem Einschluss und die geplante Studienteilnahme innerhalb der ersten Woche nach der Operation<br>• 2 oder mehr Carcinome<br>• Mangelndes deutsches Sprachverständnis<br>• Unterbringung in einer Anstalt auf gerichtliche oder behördliche Anordnung<br>• fehlende Bereitschaft zur Speicherung und Weitergabe pseudonymisierter Studiendaten<br>• Mitarbeiter der Charité
b. **Teilnehmerinformation** (wer diese mündlich erteilt und Angabe, wie viel Zeit zwischen Aufklärung und Einwilligung verbleibt, ansonsten Verweis auf deren Inhalt als Anlage möglich)	Siehe Anhang Nr. 2

c. **Einwilligungserklärung** (Verweis auf deren Inhalt als Anlage möglich)	S. Anhang Nr. 3
d. Ggf. **Information und Einwilligung des gesetzlichen Vertreters** (ggf. auch Beschreibung des Verfahrens zur Einrichtung einer gerichtlichen Betreuung)	Entfällt
17. Maßnahmen zur Gewinnung von Studienteilnehmern (Aushang ?, Zeitungsannoncen? Etc.)	Studienteilnehmer können nur Patientinnen und Patienten sein, die in der Charité bzw. den anderen teilnehmenden Universitätskliniken operiert werden. Diese Patienten werden präoperativ um die Teilnahme an der Studie gebeten.<br>Es werden keine Studienteilnehmer aktiv oder durch Rekrutierungsanzeigen angeworben.
18. Ggf.: **Grund für die Einbeziehung und Darlegung des therapeutischen Nutzen für Personen, die minderjährig und/oder nicht einwilligungsfähig sind.**	Entfällt
19. Beziehung zwischen Studienteilnehmer und Studienarzt/-ärztin	Der/die Studienarzt/ärztin ist in der Regel nicht zugleich auch behandelnder Arzt/Ärztin, jedoch in der gleichen Klinik beschäftigt.<br>Die Patienten können frei über die Teilnahme an der Studie entscheiden, ohne dass ihre Entscheidung zur oder gegen die Teilnahme Einfluss auf ihre klinische Versorgung hätte.
20. Ggf. Erklärung zur Einbeziehung möglicherweise vom Sponsor oder Studienarzt abhängiger Personen	Entfällt
21. Maßnahmen, die eine Feststellung zulassen, ob ein Studienteilnehmer an mehreren Studien zugleich oder vor Ablauf einer in der vorangegangenen Studie festgelegten Frist teilnimmt.	Im Rahmen der Einschlussuntersuchung wird der Patient zu paralleler Teilnahme an einer anderen Studie befragt. Wenn er Teilnehmer einer anderen Studie ist oder die Studie erst innerhalb der letzten Woche vor dem eventuellen Einschluss beendet worden ist, liegt ein Ausschlusskriterium für die Teilnahme an der Studie vor.
22. Ggf.: Honorierung bzw. Kostenerstattung der Studienteilnehmer (Höhe, wofür soll	Entfällt

gezahlt werden ?)	
23. Ggf.: Plan für die Weiterbehandlung und medizinische Betreuung der betroffenen Personen nach dem Ende der Studie	Die Studiendauer ist begrenzt auf den Krankenhausaufenthalt des Patienten. Weiterhin wird eine Befragung nach drei und zwölf Monaten hinsichtlich der Lebensqualität durchgeführt. Da der Patient nur nach Erfüllung definierter Entlassungskriterien aus dem Krankenhaus entlassen wird, ist damit die Nachsorge während der Studie gewährleistet. Eine Betreuung über das Studienende hinaus ist nicht vorgesehen.
24. Ggf.: Versicherung der Studienteilnehmer (Versicherer, Versicherungsumfang, Versicherungsdauer)	Die Patienten sind durch die behandelnde Institution im Rahmen ihrer klinischen Behandlung versichert.<br>Der Studienarzt ist durch die Betriebshaftpflichtversicherung der Charité – Universitätsmedizin Berlin gegen Haftungsansprüche, welche aus seinem schuldhaften Verhalten resultieren könnten, versichert.<br>Siehe Anhang Nr. 5 E-Mail an Frau E. Eckert zur Bestätigung der Betriebshaftpflicht des Krankenhauses für die Studie
25. Ggf.: Dokumentationsverfahren (Verweis auf CRF-Bögen möglich)	Die Datenerhebung wird mittels elektronischer CRF durchgeführt. Hierzu wird die Studiensoftware in Zusammenarbeit mit der KKS der Charité in SecuTrial erstellt. Gemäß GCP wird die Datenbank mit einem Log-Buch programmiert, so dass sämtliche Änderungen der Daten dokumentiert sind und die Software Plausibilitäts-, Konsistenz- und Rangecheckssowie einen Audit-Trail ermöglicht.<br><br>Die Studiendaten werden online erfasst und direkt in die Datenbank des Studienservers übertragen. Die Datenübertragung zwischen dem Arbeitsplatzrechner (im jeweiligen Studienzentrum) und dem Studienserver erfolgt über eine gesicherte Verbindung (SSL-Verschlüsselung), so dass die übertragenen Studiendaten nicht manipuliert werden können.<br><br>Die Patientendaten werden nur pseudonymisiert abgespeichert. Die Studiensoftware generiert für jeden neu anzulegenden Patient automatisch ein Pseudonym. Die eindeutige Zuordnung zum Patienten erfolgt über einen Papier-Ausdruck, welcher sicher aufbewahrt werden muss.
26. Ggf.: Beschreibung, wie der Gesundheitszustand gesunder betroffener Personen dokumentiert werden soll	Es werden keine gesunden Personen eingeschlossen. Alle Patienten sind ≥ 65 Jahre und an einem Malignom erkrankt. Es wurde im Vorfeld, unabhängig von der Studie, die Indikation für eine onkologisch-chirurgische Therapie gestellt.
27. Ggf.: Methoden, unerwünschte Ereignisse festzustellen, zu dokumentieren und mitzuteilen (wann, von wem und wie ??)	Entfällt<br>.
28. Vorgehen zum Schutz der Geheimhaltung der gespeicherten	Die Datensätze im eCRF werden ausschließlich pseudonymisiert gespeichert. Die Patientenidentifikationsliste, die eine Verbindungsherstellung zwischen einem Patienten und einem Datensatz erlaubt, ist nur autorisierten Personen zugänglich und wird verschlossen aufbewahrt. Das Codierungsschema für die Pseudonymisierung wird vom

Daten, Dokumente und ggf. Proben, Darlegung der Verschlüsselung der Daten von Studienteilnehmern	elektronischen System Secutrial automatisch generiert.
29. Erklärung zur Einhaltung des Datenschutzes	Die Vorschriften des Datenschutzrechtes werden eingehalten.<br>Siehe Name und Unterschrift des/der Antragstellers.<br>Siehe Anhang Nr. 6 schriftliche Meldung zur Kenntnisnahme beim Datenschutzbeauftrag<br><br>Die Studienärzte werden die personenbezogenen Daten für Zwecke der Verwaltung und Durchführung der Studie sowie für Zwecke der Forschung und statistischen Auswertung verwenden.<br><br>Für jegliche Weitergabe von Daten an Beteiligte an der Studie werden die Daten mit einer Codenummer versehen (Pseudonymisierung der Daten). Auf den Codeschlüssel, der es erlaubt, die studienbezogenen Daten mit dem Patienten in Verbindung zu bringen, haben nur der Studienarzt und seine Mitarbeiter Zugriff. Sämtliche Aufzeichnungen, anhand derer die Patienten identifiziert werden können, werden streng vertraulich behandelt.<br><br>Alle an der Studie beteiligten Personen sind bezüglich Vertrautheit, ärztlicher Schweigepflicht und über die Richtlinien des GCP aufgeklärt.
30. Namen und Anschriften der Einrichtungen, die als Studienzentrum oder Studienlabor in die Studie eingebunden sind, sowie der Studienleiter und die Studienärzte	Leiter der klinischen Studie:<br>Prof. Dr. Claudia Spies<br>Universitätsklinik für Anästhesiologie mit Schwerpunkt operative Intensivmedizin,<br>Charité – Universitätsmedizin Berlin,<br>Charité Campus Mitte<br>Charitéplatz 1, 10117 Berlin<br>Charité Campus Virchow Klinikum<br>Augustenburger Platz 1, 13353 Berlin<br>Tel. +49 30 450 551001/02<br>Fax: +49 30 450 551909<br>anaesthesie-virchow-klinikum@charite.de<br><br>**<u>Studienärzte:</u>**<br>OÄ Dr. Maren Schmidt<br>Klinik für Anästhesiologie und operative Intensivmedizin,<br>Charité – Universitätsmedizin Berlin,<br>Charité Campus Virchow Klinikum<br>Augustenburger Platz 1, 13353 Berlin<br>Tel: +49 (0) 30 450 651197<br>Fax: +49 (0) 30 450 551909<br>E-Mail: maren.schmidt@charite.de<br><br>Dr. Jan Philip Breuer<br>Klinik für Anästhesiologie und operative Intensivmedizin,<br>Charité – Universitätsmedizin Berlin,<br>Charité Campus Mitte<br>Charitéplatz 1, 10117 Berlin<br>Charité Campus Virchow Klinikum<br>Augustenburger Platz 1, 13353 Berlin<br>Tel.: +49 (0) 30 450 631093<br>Fax: +49 (0) 30 450 531 911<br>E-Mail: philip.breuer@charite.de

	Zentrale Auswertungsstelle:<br>**Statistiker**<br>Prof. Dr. Klaus-Dieter Wernecke<br>Biometrie und Statistik<br>Wildensteiner Str 27<br>10318 Berlin<br>Tel: +49 30 50158763<br>Fax: +49 30 50158906<br>E-Mail: kdwernecke@sostana.com<br><br>**Beteiligte Studienzentren:**<br><br>**Ruprecht-Karls-Universität Heidelberg**<br>Universitätsklinikum Heidelberg<br>Universitätsklinik für Anästhesiologie<br>Prof. Dr. med. Eike O. Martin, (geschäftsführender Direktor)<br>Im Neuenheimer Feld 110<br>69120 Heidelberg<br>Tel.: 06221 56 6350<br>Fax.: 06221 56 5345<br><br>Universitätsklinik Mannheim<br>Klinik für Anästhesiologie und operative Intensivmedizin<br>Prof. Dr. med. Dr. h.c. Klaus van Ackern (Klinikdirektor)<br>Prof. Dr. med. Grietje Beck (Stellvertreterin des Direktors)<br>Theodor-Kutzer-Ufer 1-3<br>68167 Mannheim<br>Telefon 0621/383-2614<br>Telefax 0621/383-3806<br><br>**Ludwig-Maximilians - Universität München**<br>Universitätsklinik München<br>Klinik für Anaesthesiologie der Universität München<br>Prof. Dr. Bernhard Zwissler<br>OA Prof. Dr. M. Thiel<br>Marchioninistr. 15<br>81377 München<br>Telefon ++49 - (0) 89 - 7095 - 4551<br>Telefax ++49 - (0) 89 - 7095 – 8885<br>.
31. Angaben zur Eignung der Prüfstelle, insbesondere zur Angemessenheit der dort vorhandenen Mittel und Einrichtungen sowie des zur Durchführung der klinischen Prüfung zur Verfügung stehenden Personals und zu Erfahrungen in der Durchführung ähnlicher Studien	Die Charité sowie die anderen teilnehmenden Universitätskliniken sind Krankenhäuser der Maximalversorgung mit einer 24-stündigen Bereitschaft zur Patientenversorgung. Die teilnehmenden Abteilungen besitzen weit reichende Erfahrungen mit der Durchführung klinischer Studien.

32. Vereinbarung über den Zugang des Prüfers/Hauptprüfers/Leiter der klinischen Prüfung, zu den Daten und den Grundsätzen über die Publikation	Die Aufzeichnungen der Patientendaten werden streng vertraulich behandelt und die Weitergabe der Daten wird nur in pseudonymisierter Form erfolgen.<br>Die Studienärzte haben nur Zugang zu den personenbezogenen Daten, wie es zur Durchführung der Studie notwendig ist.<br>Die Leiterin der klinischen Studie hat Zugang zu sämtlichen Daten.<br>Die Publikationen werden unabhängig vom Ergebnis der Studie in einem Peer-Reviewed Journal angestrebt.
33. Angaben zur Finanzierung der Studie	Die Studie wird von der Deutschen Krebshilfe gefördert.<br>Laufzeit : 2 Jahre
a. Finanzierungsquelle (Name und Sitz)	Deutsche Krebshilfe e. V.; Buschstr. 32; 53113 Bonn
b. Höhe der kalkulierten Kosten pro Teilnehmer und insgesamt	Die Höhe der Kosten wurde durch die Deutsche Krebshilfe im Review Verfahren festgelegt. Drittmittelkonto ist eingerichtet.
c. Höhe der Kostenerstattung pro Teilnehmer und insgesamt	Entfällt

Literatur:

1. http://www.destatis.de/basis/d/gesu/DRG *Statistik.php*.
2. Coebergh JW, *Epidemiology in Europe*. Eur J Cancer, 2001. **37**(Suppl 7): p. 175 - 227.
3. Janssens JP, K.K., *Pneumonia in the very old*. Lancet Infect Dis, 2004. **4**: p. 112 - 124.
4. Iscoe N., T.P., Gort E, Wu K,, *Cancer surgery in the elderly*. Ann Oncol, 1998. **9**(suppl 3): p. absr 109.
5. Irvin TT, *Prognosis of colorectal cancer in the elderly*. Br J Surg, 1988. **75**: p. 419-421.
6. Goodwin J.S., S.J.M., Hunt W.C., *Determinants of Survival in Older Cancer Patients*. J Natl Cancer Inst, 1996. **88**(15): p. 1031- 1038.
7. Mangano DT, *Perioperative Cardiac Morbidity*. Anesthesiology, 1990. **72**: p. 155-184.
8. Seymour DG, P.R., *Postoperative complications in the elderly surgical patient*. Gerontology, 1983. **29**: p. 262- 270.
9. Audisio RA, *The surgical risk of elderly patients with cancer*. Surg Oncol, 2004. **13**(4): p. 169 - 173.
10. Ono S, A.S., Tsujimoto H, Ueno C, Mochizuki H. , *Increased monocyte activation in elderly patients after surgical stress*. Eur Surg Res, 2001. **33**: p. 33 - 38.
11. Spies CD, E.V., Szabo G, Lau A, von Dossow V, Schoenfeld H, Althoff H, Hegenscheid K, Bohm B, Schroeder T, Pfeiffer S, Ziemer S, Paschen C, Klein M, Marks C, Miller P, Sander M, Wernecke KD, Achterberg ER, Kaisers U, lenzenhuber E, Volk HD, *Intervention of the Neuroendocrine-Immune Axis and Postoperative Pneumonia Rate in Alcoholic Patients*. . Am J Resp Crit Care 2006. **174**: p. 408 - 414.
12. Spies CD, S.C., Weiß-Gerlach E, Neuner B, Neumann T, von Dossow V, Schenk M, Wernecke KD, Elwyn G. , *Preferences for shared decision making in chronic pain*

patients compared to patients in premedication visit. . Acta Anaesthesiol Scand, 2006. **50**: p. 1019 - 1026.

13. Otter H, M.J., Bäsell K, Heymann von C, Vargas Hein O, Böllert P, Pattariya J, Behnisch I, Wernecke KD, Konertz W, Loening S, Blohmer J, Spies *Validity and reliability of the DDS for severity of delirium in the ICU*. Neurocrit Care, 2005. **2**(150 - 158).
14. Heymann C von, S.H., Sander M, Ziemer S, Grubitzsch H, Spies C, *Clopidogrel-Related Refractory Bleeding after Coronary Artery Bypass Graft Surgery: A Rationale for the Use of Coagulation Factor Concentrates?* heart Surgery Forum, 2005. **8**: p. 39 - 41.
15. Martin J, P.A., Franck M, Wernecke KD, Fischer M, Spies C, *Practice of sedation and analgesia in German intensive care units: result of a national survey*. Crit Care, 2005. **9**: p. 117 - 123.
16. Ponzetto M, M.B., Maina P, Rosato R, Ciccone G, Merletti F, Rubenstein LZ, Fabris F, *Risk factors for early and late mortality in hospitalized older patients: the continuing importance of functional status*. J Gerontol A Biol Sci Med Sci, 2003. **43**: p. M59 - M63.
17. Pope D, R.H., Gennari R, Corsini G, Maffezini M, Hoekstra HJ, Mobarak D, Sunouchi K, Stotter A, West C, Audisio RA, *Pre-operative assessment of cancer in the elderly (PACE): A comprehensive assessment of underlying characteristics of elderly cancer patients prior to elective surgery*. Surgical Oncology 2007. **15**: p. 189 - 197.
18. Audisio RA, B.F., Gennari R, Jaklitsch MT, Koperna T, Longo WE, Wiggers T ,Zbar AP,, *The surgical management of elderly cancer patients: recommendations of the SIOG surgical task force*. Eur J Cancer, 2004. **40**: p. 926 - 938.
19. Gaston CM, M.G., *Information giving and decision-making in patients with advanced cancer: A systematic review*. Social Science & Medicine, 2005. **61**: p. 2252 - 2264.
20. Neuhauser D., *The coming third healthcare revolution: personal empowerment*. Qual Manag Health Care, 2003. **12**: p. 171 - 184.
21. Peterson MG, C.-M.J., Finerty E., Graziano S., King S., Sculco TP, *Effectiveness of best practice implementation in reducing hip arthroplasty length of stay*. J Arthroplasty, 20088. **23**(1): p. 69 - 73.
22. Golant M., A.T., Martin C., , *Managing cancer side effects to improve quality of life: a cancer psychoeducation program*. Cancer Nurs, 2003. **26**(1): p. 37 - 44.
23. Mishra SI et al., *Improving breast cancer control among Latinas: evaluation of a theory-based educational program*. Health Education& Behavior, 1998. **25**(5): p. 653 - 670.
24. Davison, B.J., Degner LF, *Empowerment of men newly diagnosed with prostate cancer*. Cancer Nurs, 1997. **20**(3): p. 187 - 196.
25. Wetzels R., H.M., Van Weel C., Grol R., Wensing M., *Interventions for improving older patients' involvement in primary care episodes*. Cochrane Data Base Syst Rev, 2008(4).
26. Mazur DJ., H.D., *The effect of physicians's explanation on patient's treatment preferences: five -year survival data*. Med Decis Making, 1994. **14**: p. 255 - 258.
27. Warburton DE, N.C., Bredin SS, *Health benefits of physical activity: the evidence*. CMAJ, 2006. **14**(174): p. 801 - 809.
28. Ringdal GI, R.K., Jordhoy MS, Kaasa S, *Does social support from family and friends work as a buffer against reactions to stressful life events such as terminal cancer ?* Palliat Support Care, 2007. **5**: p. 61 - 69.
29. Dancey J, Z.B., Osoba D, , *Quality of life scores: an independent prognostic variable in a general population of cancer patients receiving chemotherapy. The National Cancer Institute of Canada Clinical Trials Group*. Qual LifeRes, 1997. **6**: p. 151 - 158.

30. Kaasa S., M.A., Lund E., , *Prognostic factors for patients with inoperable non-small cell lung cancer, limited disease. The importance of patients' subjective experience of disease and psychosocial well-being*. Radiother Oncol, 1989. **15**: p. 235-242.
31. Efficace F., B.A., Smit E.F., Lianes P., Legrand C., Debruyne C., Schramel F., Smit H.J., Gaafar R., Biesma B., Manegold C., Coens C., Giaccone G., Van Meerbeck J.,, *Is a patient's self-reported health-related quality of life a prognostic factor for survival in non-small-cell lung cancer patients? A multivariate analysis of prognostic factors of EORTC study 08975*. Ann Oncol, 2006. **17**: p. 1698-1704.
32. Maisey N.R., N.A., Watson M., Allen M.J., Hill M.E., Cunningham D., *Baseline quality of life predicts survival in patients with advanced colorectal cancer*. Eur J Cancer, 2002. **38**: p. 1351-1357.
33. Camilleri-Brennan J., S.R., *Prospective analysis of quality of life and survival following mesorectal excision for rectal cancer*. Br J Surg, 2001. **88**(12): p. 1617-1622.
34. Coates A, P.F., Osoba D, *Quality of life in oncology practice: prognostic value of EORTC QLQ-C30 scores in patients with advanced malignancy*. Eur J Cancer, 1997. **33**: p. 1025 - 1030.
35. Merlani P, C.C., Mariotti N, Ricou B, *Long-term outcome of elderly patients requiring intensive care admission for abdominal pathologies: survival and quality of life*. Acta Anaesthesiol Scand, 2007. **51**: p. 530 - 537.
36. Depla MF, P.A., de Lange J, *New residents of residential care homes for the elderly. The problems of elders with mental disorders*. Tijdschr Gerontol Geriatr, 1999. **30**: p. 121 - 128.
37. Daugherty C., e.a., *Perceptions of cancer patients and their physicians involved in phase I trials*. J clin Oncol, 1995. **13**(5): p. 1062 - 72.
38. Houts PS., e.a., *The Prepared Family Caregiver: A Problem-solving Approach to Family Caregiver Education*. Patient Education & Counseling, 1996. **27**(1): p. 63 - 73.
39. Penedo FJ., D.J., Molton I., Gonzalez JS., Kinsinger D., Roos BA., Carver CS., Schneiderman N., Antoni MH., , *Cognitive-Behavioral Stress Management Improves Stress-Management Skills and Quality of Life in Men Recovering from Treatment of Prostate Carcinoma*. Cancer, 2003. **100**(192 - 200).
40. Carlson L.E., S.M., Patel K.D., Goodey E., , *Mindfulness-based stress reduction in relation to quality of life, mood, symptoms of stress and levels of cortisol, dehydroepiandrosterone sulfate (DHEAS) and melatonin in breast and prostate cancer outpatients*. Psychoneuroendocrinology, 2004. **29**(4): p. 448 - 74.
41. Antoni MH., L.J., Kilbourn KM., Boyers AE., Culver JL, Alferi SM., Yount SE., Mc Gregor BA., Arena PL., Harris SD., Price AA., Carver CS.,, *Cognitive-behavioral stress management intervention decreases the prevalence of depression and enhances benefit finding among women under treatment for early-stage breast cancer*. Health Psychol, 2001. **20**: p. 20-32.
42. Goodwin J.S., E.M., Bordelau LJ, Pritchard KI, Trudeau ME, Koo J., Hood N.,, *Health-related quality of life and psychosocial status in breast cancer prognosis: analysis of multiple variables*. J clin Oncol, 2004. **22**(20): p. 4184 - 4192.
43. Kassane DW., L.A., Hatton A., Bloch S., Smith G., Clarke DM., Miach P, Ikin J. , Ranieri N. Snyder RD., , *Effect of cognitive - existentioal group therapy on survival in early stage breast cancer*. J clin Oncol, 2004. **22**(21): p. 4255 - 60.

44. Liebermann M., G.M., Altman T.,, *Therapeutic Norms and Patient benefit: Cancer Patients in Professionally Directed Support Groups*. Group Dynamic, Theory, Research and Practice. **8**(4): p. 265-276.
45. Weiss-Gerlach, E., et al., *Motivation of trauma patients to stop smoking after admission to the emergency department*. Addictive Behaviors, 2008. **33**(7): p. 906-918.
46. Neuner, B., et al., *Socioeconomic factors, hazardous alcohol consumption, and smoking in patients with minor trauma in an inner-city emergency department*. Journal of Emergency Medicine. **In Press, Corrected Proof**.
47. Neuner, B., et al., *Hazardous alcohol consumption and sense of coherence in emergency department patients with minor trauma*. Drug and Alcohol Dependence, 2006. **82**(2): p. 143-150.
48. Schoenfeld, H., et al., *The Effect of Stress-Reducing, Low-Dose Ethanol Infusion on Frequency of Bleeding Complications in Long-Term Alcoholic Patients Undergoing Major Surgery*. The American Surgeon, 2007. **73**: p. 192-198.
49. Spies, C., Neuner B., Neumann T., et al., *Intercurrent complications in chronic alcoholic men admitted to the intensive care unit following trauma*. Intensive Care Med, 1996. **22**(4): p. 286 - 293.

Name und Unterschrift des/der Antragstellers:

Ich versichere hiermit, dass die in diesem Antrag gegebenen Informationen richtig sind. Ich bin der Auffassung, dass es möglich ist, die o.g. Studie in Übereinstimmung mit dem Protokoll, den nationalen Rechtsvorschriften durchzuführen.

Mir ist bekannt, dass ich gemäß §19 Berliner Datenschutzgesetz (BlnDSG) verpflichtet bin, für automatisierte Verarbeitungen personenbezogener und personenbeziehbarer Daten eine Datei- und Verfahrensbeschreibung zu erstellen und diese gemäß §19a dem behördlichen Datenschutzbeauftragten der Charité zur Verfügung stellen muss. Ich bin darüber informiert, dass wenn es sich um ein Verfahren handelt, mit dem Daten verarbeitet werden, die einem Berufsgeheimnis (z.B. ärztliche Schweigepflicht) unterliegen, ich gemäß §5 BlnDSG vor dem Einsatz dieses Verfahrens eine Vorabkontrolle durch den behördlichen Datenschutzbeauftragten der Charité veranlassen muss und ich das Verfahren erst bei positivem Prüfergebnis anwenden darf.

Name: Prof. Dr. Spies

Vorname: Claudia

Adresse: Augustenburger Platz 1
13353 Berlin

Position: Klinikdirektorin der Universitätsklinik für Anästhesiologie mit Schwerpunkt operative Intensivmedizin der Charité - Universitätsmedizin Berlin CCM/CVK

Datum:

Unterschrift:

Anhang:

1. Flow Chart zum Studienablauf
2. Teilnehmerinformation
3. Einwilligung zur Studienteilnahme
4. Stellungsnahme der Betriebshaftpflicht
5. Meldung an den Datenschutzbeauftragten

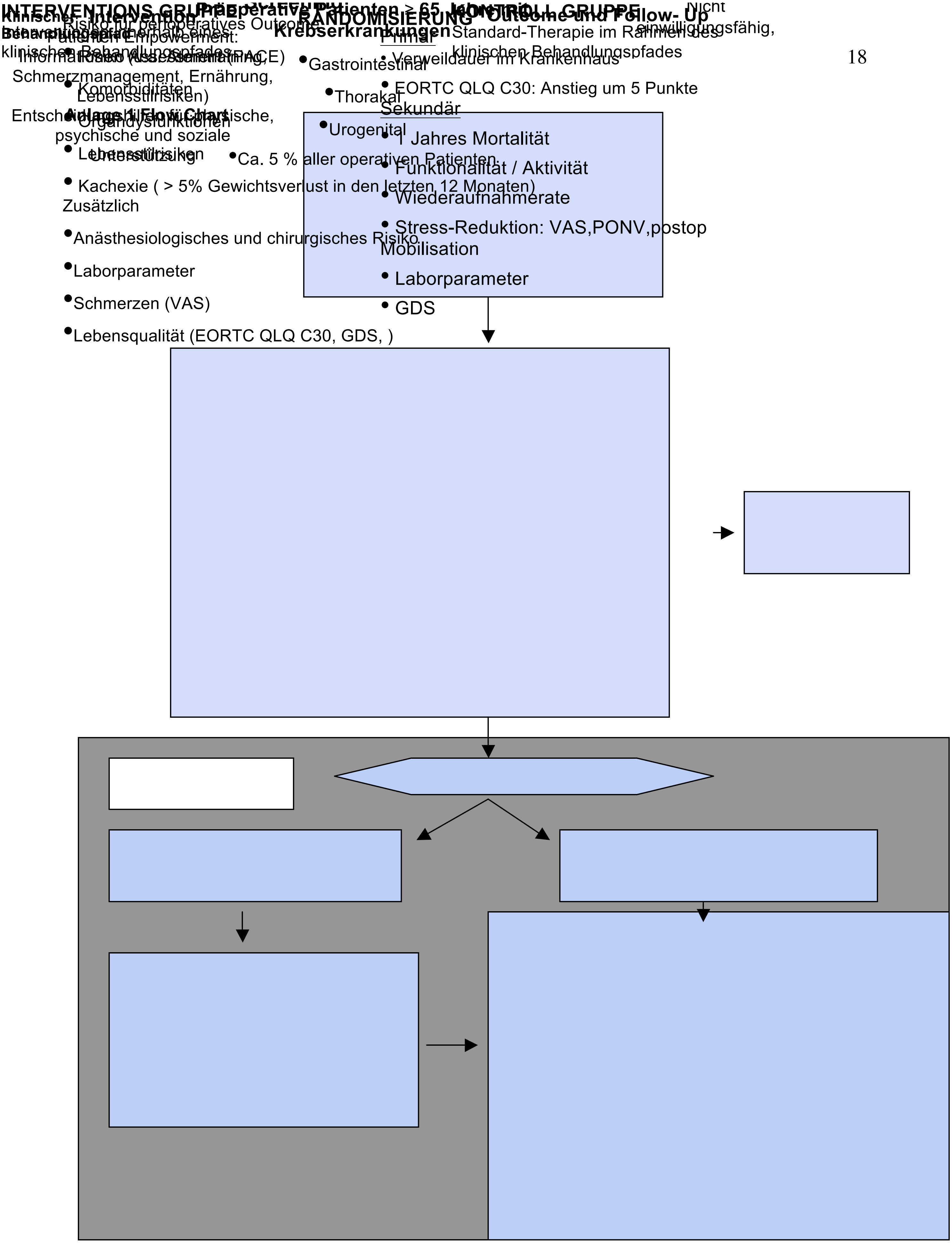
Anlage 1 Flow Chart
Präoperative Patienten > 65 Jahre mit Krebserkrankungen
Gastrointestinal
Thorakal
Urogenital
Ca. 5 % aller operativen Patienten
Nicht einwilligungsfähig,
Risiko für perioperatives Outcome
Komorbiditäten
Organdysfunktionen
Lebensstilrisiken
Kachexie (> 5% Gewichtsverlust in den letzten 12 Monaten)
Zusätzlich
Anästhesiologisches und chirurgisches Risiko
Laborparameter
Schmerzen (VAS)
Lebensqualität (EORTC QLQ C30, GDS,)
RANDOMISIERUNG
INTERVENTIONS GRUPPE
KONTROLL GRUPPE
Interventionen innerhalb eines klinischen Behandlungspfades
Patienten Empowerment:
Schmerzmanagement, Ernährung, Lebensstilrisiken)
psychische und soziale
Unterstützung
Standard-Therapie im Rahmen des klinischen Behandlungspfades
Outcome und Follow- Up
Primär
Verweildauer im Krankenhaus
EORTC QLQ C30: Anstieg um 5 Punkte
Sekundär
1 Jahres Mortalität
Funktionalität / Aktivität
Wiederaufnahmerate
Stress-Reduktion: VAS,PONV,postop Mobilisation
Laborparameter
GDS

Anlage 2 Teilnehmerinformation

ChartéCentrum für Anästhesiologie, OP-Management und Intensivmedizin

Charité - Universitätsmedizin Berlin | D - 13344 Berli

Patientenkleber

Universitätsklinik für Anästhesiologie
mit Schwerpunkt operative Intensivmedizin
CCM / CVK

Klinikdirektorin:
Univ.-Prof. Dr. C. Spies

Zertifiziert nach DIN EN ISO 9001

Campus Virchow-Klinikum
Augustenburger Platz 1
13353 Berlin
Tel: +49 30 450 551-001
Fax: +49 30 450 551909
anaesthesie-virchow-klinikum@charite.de

Campus Charité Mitte
Charitéplatz 1
10117 Berlin
Tel: +49 30 450 531-012
Fax: +49 30 450 531911
anaesth@charite.de

http://www.charite.de/ch/anaest/

Patienteninformation

Version 1.0 vom 25.11.2008

zur Teilnahme an der Studie

„Patientenmitbestimmung und Risiko-gestützte Behandlung zur Verbesserung des Outcomes bei älteren Patienten nach gastrointestinaler, thorakaler und urogenitaler Onkochirurgie“
Kurztitel: PERATECS

Studienleiter: Univ.-Prof. Dr. C. Spies

Sehr geehrte Patientin, sehr geehrter Patient,

Ihnen ist vorgeschlagen worden, an der oben genannten Studie teilzunehmen. Ältere Menschen sind die am schnellsten wachsende Bevölkerungsgruppe in der westlichen Welt. Ebenfalls nimmt die Zahl der Operationen bei älteren Menschen zu. Als Folgen von operativen Eingriffen können bei den genannten Patienten vermehrt Komplikationen wie zum Beispiel Entzündungen im Wundgebiet oder Lungenentzündungen, Herz-Kreislauf-Komplikationen auftreten. Wichtig ist speziell bei älteren Patienten die Bedeutung der Begleiterkrankungen zu erfassen, um spezielle altersbedingte Risikofaktoren festlegen zu können.
Um ein speziell auf ältere Patienten zugeschnittenes Risikoprofil erstellen zu können, werden Ihnen verschiedene Fragebögen ausgehändigt, die zum einen nach Ihren Nebenerkrankungen, Ihrer körperlichen Leistungsfähigkeit sowie Ihrem Ernährungszustand fragen. Aber auch Ihre Lebensqualität vor und nach der Operation wird erfasst.
Eine Teil der Patienten (Studiengruppe) erhält außerdem zusätzlich ein Tagebuch, in dem alle relevanten Informationen zum Verlauf des Krankenhausaufenthaltes verzeichnet sind. Es wird

Ihnen zum Beispiel genau erklärt bis wann Sie vor der Operation essen und trinken dürfen. Aber auch ab wann Sie beispielsweise ab einer bestimmten Schmerzstärke ein zusätzliches Medikament bekommen können. Weiterhin erhält diese Patientengruppe sowohl vor als auch nach der Operation Informationen von Seiten der Selbsthilfegruppen bzw. Patientenvertreter. Diese Information betreffen die Erkrankung an sich, vor allem aber auch die Möglichkeiten, nach der Krankenhausentlassung Unterstützung zu bekommen.
Die andere Hälfte der Patienten, die sog. Kontrollgruppe erhält nur die oben genannten Fragebögen zu Erstellung eines Risikoprofils.
Die Studie findet an der Charité – Universitätsmedizin Berlin, Klinik für Anästhesiologie und operative Intensivmedizin, Campus Virchow-Klinikum und Campus Charité Mitte unter der Leitung von Prof. Dr. Claudia D. Spies statt. Weitere Studienzentren sind das Universitätsklinikum Heidelberg/Mannheim und das Klinikum der Universität München.
Die Studie verfolgt keine kommerziellen Interessen.

Zweck der Studie
Es soll ein speziell auf ältere Patienten zugeschnittenes Risikoprofil erstellt werden.
Der Zweck der Datenerhebung soll sein, eine präoperative Risikoanalyse speziell für ältere Patienten zu erheben, um Möglichkeiten der Risikoreduktion finden zu können.
Weiterhin soll untersucht werden, ob eine vermehrte Information und aktive Einbindung der Patienten in ihre Therapie einen Einfluss auf den Krankheitsverlauf hat.
Der Zweck der Datenerhebung ist die Überprüfung der Hypothese, dass eine vermehrte Information und aktive Einbindung der Patienten in ihre Therapie einen positiven Einfluss auf den Krankheitsverlauf hat.

Freiwilligkeit der Teilnahme

Die Teilnahme an dieser Studie freiwillig und Sie können jederzeit ohne Angabe von Gründen von der Zusage zur Teilnahme zurücktreten, ohne dass Ihnen hieraus Nachteile für Ihre weitere Behandlung erwachsen. Sie erhalten dann in jedem Fall eine Therapie (Behandlung), die dem aktuellen Wissensstand entspricht.

Ablauf und Dauer der Teilnahme
Die von uns geplante Untersuchung findet als so genannte „klinische Studie" statt.
Wir möchten Sie bitten, vor der Operation einige Fragebögen zu beantworten. Diese Fragebögen enthalten Fragen zu Ihrem Lebensstil, Ihrer Lebensqualität, zu Ihren Begleiterkrankungen und dabei auch Fragen bezüglich Stresses und riskanten Gesundheitsverhaltens. Einige sozioökonomische Fragen werden erhoben, um sie mit dem repräsentativen Gesundheitsumfrage des Robert-Koch-Instituts vergleichen zu können.
Die Befragung vor der Operation nimmt ca. 45 Minuten in Anspruch. An den ersten fünf Tagen nach der Operation werden Sie von Studienmitarbeitern bzw. Doktoranden besucht, die Ihnen Fragen nach Ihrem Befinden stellen. Der Zeitaufwand pro Tag beträgt ca. 10 Minuten. An Ihrem Entlassungstag wird ebenfalls eine kurze Befragung stattfinden, die höchstens 15 Minuten dauert. Außerdem erhalten Sie 3 und 12 Monate nach Ihrer Entlassung Fragebögen zu Ihrer Lebensqualität zugesandt. Die Beantwortung dieser Fragebögen dauert ca. 20 Minuten.

Diese Fragebögen erhalten alle teilnehmenden Patienten.

Weitere Daten, die aufgezeichnet werden, sind allgemeine Angaben wie Name, Anschrift, Telefonnummer, Alter, Geschlecht, Daten zu ihrer physischen Gesundheit wie medizinische Diagnosen und Routine-Laborparameter.
Die Patienten, die in der Studiengruppe sind, erhalten zusätzlich vor und nach der Operation Informationen von Selbsthilfe- und Patientenvertretern. In diesen Gesprächen werden Informationen über die Krankheit, den Aufenthalt im Krankenhaus, vor allem aber auch Möglichkeiten der Unterstützung nach der Entlassung aus dem Krankenhaus gegeben.
Weiterhin erhalten Patienten in dieser Gruppe ein Patiententagebuch, dessen tägliche Führung nicht mehr als 10 Minuten bedarf.
Am Anfang des Tagebuches finden sich allgemeine Informationen, die Sie bitte durchlesen. Anschließend finden sich Fragen, bei denen die entsprechende Antwort angekreuzt wird. Sollten Sie sich nicht ausreichend informiert fühlen, fragen Sie bitte Ihren behandelnden Arzt.
Im Anschluss zu den von Ihnen auszufüllenden Fragen finden sich Anweisungen. Es wird Ihnen zu Beispiel die Information gegeben, dass Sie ab einer bestimmten Schmerzstärke ein zusätzliches Medikament bekommen können. Bitte halten sie in diesem Fall Rücksprache mit Ihrem behandelnden Arzt. Füllen sie die Seiten den Tagen entsprechend nacheinander aus. Um den Umfang des Buches nicht zu groß werden zu lassen, endet es am fünften Tag nach der Operation. Falls Sie länger in unserem Haus sein sollten, füllen Sie bitte die letzten Seiten dann am Entlassungstag aus. Sollten Sie vor dem fünften Tag entlassen werden können Sie die Tage die Sie nicht betreffen überspringen. Beantworten Sie bitte dann an Ihrem Entlassungstag die entsprechende Seite.
Auch in dieser Gruppe wird 3 und 12 Monate nach Entlassung eine Befragung hinsichtlich der Lebensqualität durchgeführt, sofern Sie zustimmen. Auch bei diesen Anrufen bzw. Anschreiben können Sie selbstverständlich jederzeit die Beantwortung unserer Fragen ablehnen und/oder eine Beendigung ihrer Nachbefragung wünschen.

Mögliche Unannehmlichkeiten und Risiken
Für Sie bedeutet die Studie eine geringe zeitliche Mehrbelastung (Zeitangaben s. o.). Risiken ergeben sich für Sie keine.

Umstände, die zum Abbruch der Studienteilnahme führen
Widerruf der Teilnahme durch den Studienteilnehmer bzw. die Studienteilnehmerin.

Nutzen
Der Nutzen besteht für Sie in einer zum einen in einer grundlegenden Information über Ihr Risikoprofil. Weiterhin erhalten Sie zusätzliche grundlegende Informationen über den Verlauf Ihres Krankenhausaufenthaltes und die Möglichkeiten der Unterstützung nach der Krankenhausentlassung. Es wird Ihnen ermöglicht, aktiv an Ihrer Therapie mitzuwirken.
Sie können jederzeit mit Studienmitarbeitern Fragen zur Teilnahme und zu Ergebnissen Ihrer Untersuchungen klären. Sie können sich auch zu jedem späteren Zeitpunkt gerne mit Mitarbeitern der Studie in Verbindung setzen, wenn Sie eine weiter gehende Beratung bezüglich der bei dem Führen des Heftes auftretenden Probleme haben wollen oder eine weitergehende Beratung bezüglich Ihrer Lebensstilrisiken wünschen.

Alternativen zur Teilnahme

Wenn Sie sich nicht zur Teilnahme entschließen können, erhalten Sie in jedem Fall eine Behandlung, die dem aktuellen Wissensstand entspricht.

Pflichten des Teilnehmers:
Die Pflichten des Teilnehmers während der Studienphase liegen in der nach Möglichkeit vollständigen und wahrheitsgemäßen Beantwortung bei den Befragungen und bei der Protokollierung der einzelnen Prozessschritte in dem Patiententagebuch.

Neue Erkenntnisse
Sollten im Verlauf der Studie neue wissenschaftliche Erkenntnisse bekannt werden, die eine bessere Therapie/Behandlung bzw. eine Änderung in der Therapie/Behandlung nach sich ziehen, werden Sie über diesen Sachverhalt zeitnah informiert.

Datenschutz

Die Studie wird nach den geltenden datenschutzrechtlichen Bestimmungen durchgeführt.
Ihre sämtlichen personenbezogenen, während der Studie erhobenen Daten werden vor ihrer Übermittlung pseudonymisiert. Damist sind Ihre Daten für den Empfänger anonym, d.h. er kann keine Verbindung zwischen Ihren Daten und Ihrer Person herstellen.
Durch Ihre Unterschrift auf der Einwilligungserklärung erklären Sie sich damit einverstanden, dass die Studienärzte OÄ Dr. M. Schmidt und Dr. P. Breuer und ihre wissenschaftlichen Mitarbeiter Ihre personenbezogenen Daten (Name, Anschrift, Geschlecht, Geburtsdatum, Telefonnummern, Daten zu ihrer physischen und psychischen Gesundheit oder andere Daten, die während ihrer Teilnahme an der Studie oder bei Folgeuntersuchungen erfasst wurden) für Zwecke der o.g. Studie erheben und verarbeiten sowie für die Verwaltung und Durchführung der Studie sowie für Zwecke der Forschung und statistischen Auswertung verwenden dürfen.
Weiterhin erklären Sie sich damit einverstanden, dass im Rahmen der Routinediagnostik ermittelte Parameter im Blut sowie klinische Diagnosen vom Studienarzt Ihrer Krankenakte entnommen werden. Hierfür (und nur für diese Angaben) entbinden Sie Ihren behandelnden Arzt von seiner ärztlichen Schweigepflicht.

Der Studienleiter gibt während der Studie erhobene studienbezogene Daten an von ihm beauftragte Dienstleister (Prof. Wernecke, Biometrie) in pseudonymisierrter Form weitergeben, die Ihre studienbezogenen Daten ausschließlich für den Zweck (Auswertung) der o.g. Studie verwendet.
Die an den vorgenannten Empfänger weitergebenen studienbezogenen Daten enthalten nicht Ihren Namen oder Ihre Adresse. Stattdessen versieht der Studienarzt die Studiendaten mit einer Codenummer (Pseudonymisierung der Daten). Auf den Codeschlüssel, der es erlaubt, die studienbezogenen Daten mit Ihnen in Verbindung zu bringen, haben nur der Studienarzt und seine Mitarbeiter Zugriff.
Ihre Einwilligungserklärung wird getrennt vom anderen Datensatz so aufbewahrt, dass nur die wissenschaftlichen Mitarbeiter darauf zugreifen und eine personenbezogene Zuordnung vornehmen können.

Alle Ihre persönlichen Daten, die sich beim Studienarzt befinden, können vom Leiter der Studie und/oder seinen Vertretern und Auftragsunternehmen (z.B. Monitore oder

Auditoren), sowie der unabhängigen Ethikkommission eingesehen werden. Zweck dieser Prüfungen ist, sicherzustellen, dass die Studie ordnungsgemäß durchgeführt wird und die Qualität Ihrer studienbezogenen Daten gewährleistet ist.
Sie können jederzeit der Weiterverarbeitung Ihrer im Rahmen der o.g. Studie erhobenen Daten und/oder weiteren Untersuchung der Ihnen entnommen Probe widersprechen und ihre Vernichtung bzw. Löschung verlangen.

Die erhobenen Daten werden nach Abschluss der Studie nach Ablauf von 10 Jahren gelöscht, sofern nicht gesetzliche, satzungsmäßige oder vertragliche Aufbewahrungsfristen entgegenstehen. Die Ihnen entnommenen Routine-Blutproben werden an das Labor der Charité – Universitätsmedizin Berlin zur dortigen Untersuchung übermittelt. Die Proben werden über einen Zeitraum von höchstens einem Monat im Labor gelagert und danach vernichtet.

Sie haben das Recht auf Auskunft über alle beim Studienarzt vorhandenen personenbezogenen Daten über Sie. Sie haben auch ein Anrecht auf Korrektur eventueller Ungenauigkeiten in Ihren personenbezogenen Daten. Wenn Sie eine Anfrage machen wollen, wenden Sie sich bitte an Ihren Studienarzt. Dieser wird Kontakt mit dem Leiter der Studie aufnehmen und Ihnen die Information über Ihre gespeicherten Daten zur Verfügung stellen.
Bitte beachten Sie, dass die Ergebnisse der Studie in der medizinischen Fachliteratur veröffentlicht werden können, wobei die Daten so aufbereitet werden, dass keine Rückschlüsse auf personenbezogene Angaben bestehen. Die Verarbeitung der studienbezogenen Daten liegt beim Studienleiter.

Versicherungsschutz
Es wurde keine spezielle Patientenversicherung für diese Studie abgeschlossen.
Der Studienärzte und –mitarbeiter sind jedoch durch die Betriebshaftpflichtversicherung der Charité gegen Haftungsansprüche, welche aus ihrem schuldhaften Verhalten resultieren könnten, versichert.
Sollten Sie möglicherweise aufgrund schuldhaften Handelns eines Charité–Mitarbeiters einen Schaden erleiden, wenden Sie sich bitte unverzüglich an die Studienleiterin.

Freiwilligkeit der Teilnahme
Ihre Teilnahme an der Untersuchung ist absolut freiwillig. Sie hat keinen Einfluss auf den Verlauf Ihrer Behandlung. Sie haben natürlich jederzeit die Möglichkeit, Ihr Einverständnis zu dieser Untersuchung zu widerrufen, ohne dass Ihnen Nachteile entstehen.

Kosten
Durch die Teilnahme an der Studie werden Ihnen keine weiteren Kosten entstehen.

Bezahlung
Eine Bezahlung der Studienteilnehmer ist nicht geplant.

Fragerecht / Mitteilungspflicht

Sie haben jederzeit die Möglichkeit, den Studienärzten weitergehende Fragen über alle Angelegenheiten zu stellen, welche die Studie betreffen, insbesondere auch über Risiken.

Fragen können gerichtet werden an:

Dr. P. Breuer Tel.: 030-450 631093
Prof. Dr. med. C. Spies, OÄ Dr. M. Schmidt Tel.: 030-450-551001 und - 531012
Per E-Mail an: philipp.breuer@charite.de oder maren.schmidt@charite.de
Auf dem Postwege an die oben stehende Anschrift.

Herzlichen Dank für Ihre Teilnahme,
alles Gute wünscht Ihnen

Prof. Dr. med. Claudia D. Spies
Klinikdirektorin

CHARITÉ

Anlage 3 Einverständniserklärung

CharitéCentrum für Anästhesiologie, OP-Management und Intensivmedizin

Charité - Universitätsmedizin Berlin | D - 13344 Berli

Einwilligungserklärung

Version 1.0 vom 25.11.2008

zur Teilnahme an der Studie

„Patientenmitbestimmung und Risiko-gestützte Behandlung zur Verbesserung des Outcomes bei älteren Patienten nach gastrointestinaler, thorakaler und urogenitaler Onkochirurgie“

Kurztitel: PERATECS

Studienleiter: Univ.-Prof. Dr. C. Spies

Universitätsklinik für Anästhesiologie mit Schwerpunkt operative Intensivmedizin CCM / CVK

Klinikdirektorin:
Univ.-Prof. Dr. C. Spies

Zertifiziert nach DIN EN ISO 9001

Campus Virchow-Klinikum
Augustenburger Platz 1
13353 Berlin
Tel: +49 30 450 551-001/002/022
Fax: +49 30 450 551909
anaesthesie-virchow-klinikum@charite.de

Campus Charité Mitte
Charitéplatz 1
10117 Berlin
Tel: +49 30 450 531-012/52
Fax: +49 30 450 531911
anaesth@charite.de

http://www.charite.de/ch/anaest/

Bitte lesen Sie die Patienteninformation und die Einwilligungserklärung sorgfältig durch.
Bitte fragen Sie bei allen Unklarheiten oder wenn Sie weitere Informationen wünschen.

Patient:________________________________ Patienten - Pseudonym.: ____________________

(Name, Vorname)

Ich willige in die Teilnahme an der Studie ein.

Ich bin durch den Studienarzt anhand der Patienteninformation Version 1.0 vom 25.11.2008

- mündlich und schriftlich über Ziel, Dauer, Ablauf, Rechte und Pflichten, sowie Nutzen, Risiken und Nebenwirkungen der klinischen Studie aufgeklärt worden. Ich hatte ausreichend Gelegenheit Fragen zu stellen. Diese wurden mir vom aufklärenden Studienarzt verständlich beantwortet. Außerdem habe ich Kopien der schriftlichen Patienteninformation und der Patienteneinwilligung erhalten. Ich hatte genügend Zeit, um meine Entscheidung zur Teilnahme an dieser klinischen Studie frei zu treffen.

- Ich weiß, dass meine Teilnahme an der Studie völlig freiwillig ist und dass ich diese Einwilligung jederzeit und ohne Angabe von Gründen widerrufen kann, ohne dass mir daraus Nachteile für meine weitere Behandlung entstehen.

- Im Rahmen dieser klinischen Studie sind die Studienärzte durch die Betriebshaftpflichtversicherung der Charité gegen Haftungsansprüche, welche aus ihrem schuldhaften Verhalten resultieren könnte, versichert. Die Meldung der Studie gegenüber der Betriebshaftpflichtversicherung der Charité ist erfolgt. Die

Versicherungsbedingungen wurden mir vom Studienarzt erklärt und ich habe sie verstanden.

Datenschutzerklärung

Einwilligungserklärung zur Datenverarbeitung

Ich erkläre mich damit einverstanden, dass im Rahmen dieser Studie erhobene Daten über meine Gesundheit, in Papierform und auf elektronischen Datenträgern in der Klinik für Anästhesiologie und operative Intensivmedizin, Charité, Campus Virchow Klinikum, Berlin, in der in der Patienteninformation beschriebenen Form, aufgezeichnet und weitergegeben werden dürfen.
Weiterhin erklären ich mich damit einverstanden, dass für die Routine-Laborwerte, sowie klinische Diagnosen vom Studienarzt der Krankenakte entnommen werden. Hierfür (und nur für diese Angaben) entbinde ich meinen behandelnden Arzt von seiner ärztlichen Schweigepflicht.

Ich bin damit einverstanden, dass mich die Studienärzte schriftlich oder telefonisch unter Wahrung des Datenschutzes in den nächsten 12 Monaten mehrmals kontaktieren (nach 3 und 12 Monaten).

Ich bin mit einer schriftlichen ☐ und / oder telefonischen ☐ Kontaktaufnahme einverstanden:

Straße ____ Nr. ____________

PLZ ___________ Ort ____

Telefon: _____________________________ Mobiltelefon:___________________________

Am besten tel. erreichbar: ❑ vormittags ❑ nachmittags ❑ abends ❑ sonstiges__________

Ich bin vom Studienarzt mündlich aufgeklärt worden und habe die schriftliche Patienteninformation gelesen und verstanden. Ich willige in die Teilnahme an der Studie ein.

___________________ ____

Ort, Datum Name (leserlich) und Unterschrift des Patienten

___________________ ____

Der Patient wurde von mir über Ziel, Dauer, Ablauf, Nutzen, Risiken und Nebenwirkungen der klinischen Prüfung mündlich und schriftlich aufgeklärt. Aufgetretene Fragen wurden von mir verständlich und ausreichend beantwortet. Der Patient hat ohne Zwang seine Einwilligung erteilt. Eine Kopie der schriftlichen Patienteninformation und dieser Patienteneinwilligung habe ich dem Patienten ausgehändigt.

Ort, Datum	Name (leserlich) und Unterschrift des Studienarztes

Anlage 4 Stellungsnahme zur Betriebshaftpflicht
Anlage 5 Votum des Datenschutzbeauftragten
